# Supplementary figures and images for: Zebrafish mylipb attenuates antiviral innate immunity through two synergistic mechanisms targeting transcription factor irf3
Source: PLoS Pathog. 2024 May 13;20(5):e1012227. doi: 10.1371/journal.ppat.1012227 (PMC11115282; doi:10.1371/journal.ppat.1012227)

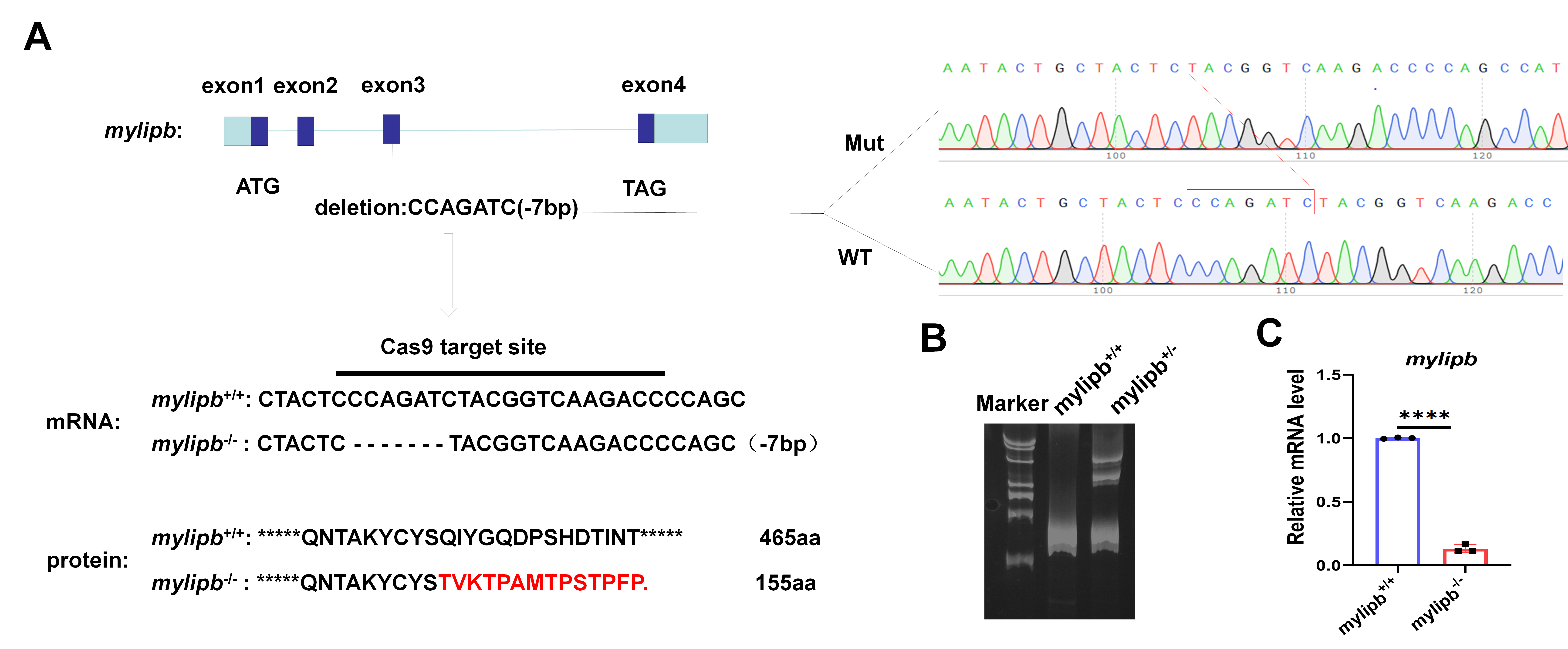

Supplement: S1 Fig — (A) The schematic of the targeting site in mylipb and the resulting nucleotide sequence in the mutant (MT, mylipbihbmb73/ ihbmb73).The predicted protein product of maoc1 in the mutant and wild-type (WT) sibling. (B) Verification of the efficiency of CRISPR/Cas9-mediated zebrafish mylipb disruption by heteroduplex mobility assay (HMA). (C) The relative mRNA levels of mylipb in the WT and homozygous mutant. All data are presented as mean values based on three repeated experiments, and error bars indicate the ± SD. *,P < 0.05, **, P<0.01; ***, P < 0.001; ****, P< 0.0001 (TIF) [file ppat.1012227.s001.tif]

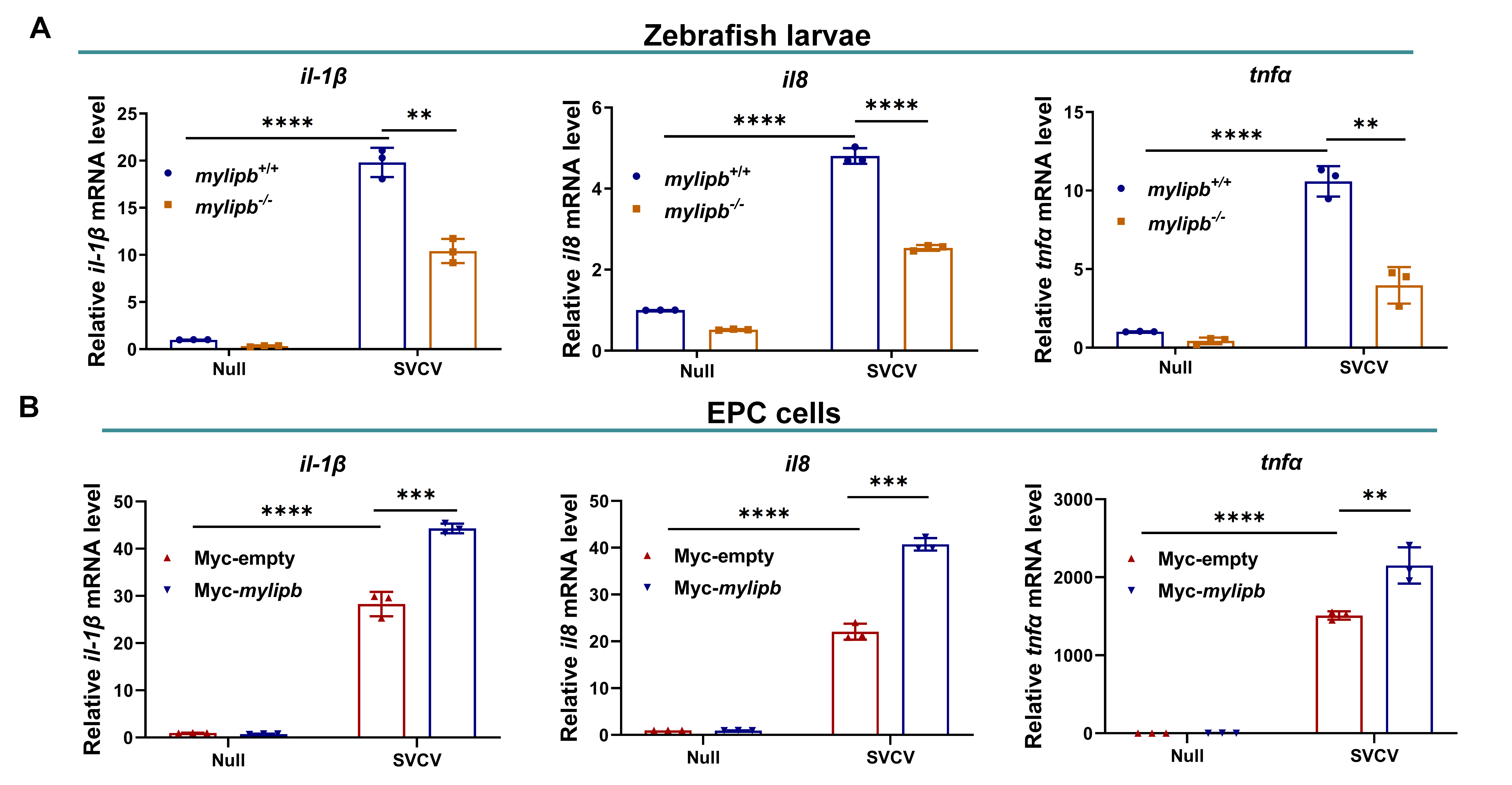

Supplement: S2 Fig — (A) Induction of genes downstream of NF-kB, including il-1β, il8, tnfα, was decreased in mylipb-/- larvae compared with WT larvae (mylipb+/+) after SVCV infection. 30 larvae (3 dpf) were pooled in a disposable 60-mm cell culture dish filled with 5 mL of egg water and 0.5 mL of SVCV (∼2.5×10^7^TCID50/ml) solution at 28°C for 24h. (B) Overexpression of mylipb suppressed expression of il-1β, il8 and tnfα induced by SVCV infection in EPC cells. EPC cells were transfected with Myc-mylipb or empty vector for 24 h and infected with SVCV (MOI: 1). After 24 h, total RNAs were extracted for examining the mRNA levels of il-1β, il8 and tnfα by qRT-PCR analysis. (TIF) [file ppat.1012227.s002.tif]

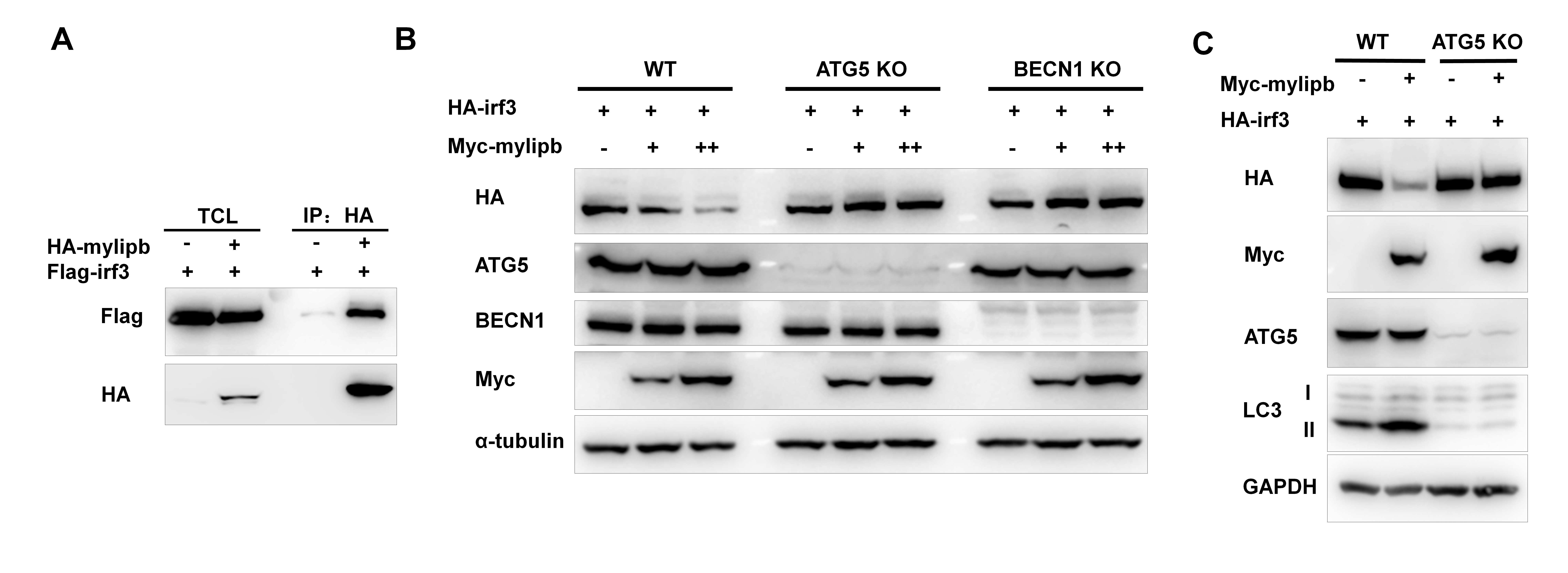

Supplement: S3 Fig — Related to Fig 4.(A) Mylipb associated with irf3. HEK293T cells seeded in 100-mm dishes were transfected with the indicated plasmids (4 μg each). After 24 h, total cell lysates were immunoprecipitated (IP) with anti-HA antibody conjugated agarose beads. Then, the immunoprecipitates and cell lysates were detected with anti-HA or anti-Flag Ab, respectively. (B, C) Wild-type (WT), ATG5 and BECN1 knockout (KO) 293T cells were co-transfected with Myc-mylipb and HA-irf3 for 24 h. The cell lysates were subjected to western blotting with the indicated antibodies. (TIF) [file ppat.1012227.s003.tif]

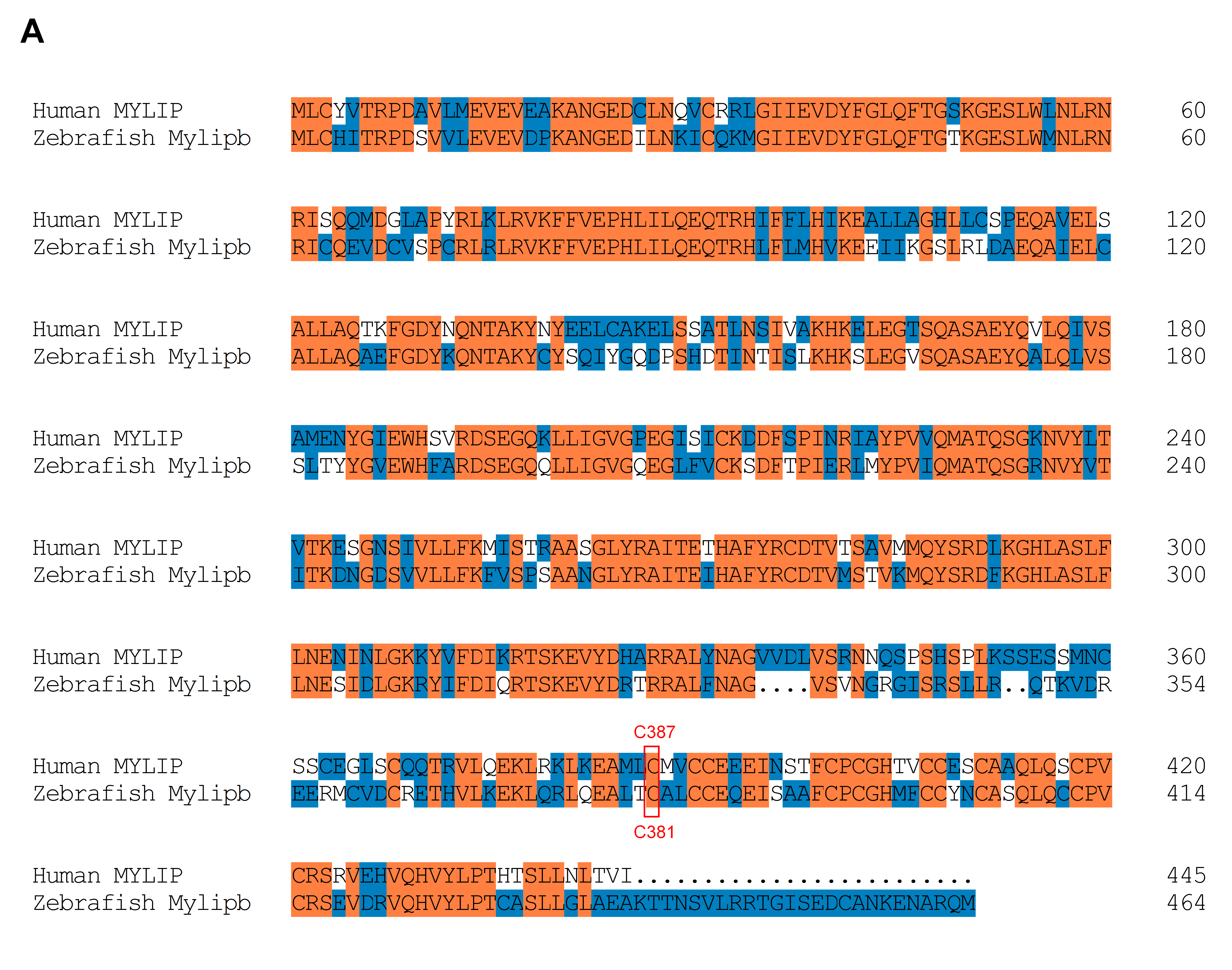

Supplement: S4 Fig — (A) Amino acid sequence alignment of human MYLIP and zebrafish mylipb. (TIF) [file ppat.1012227.s004.tif]

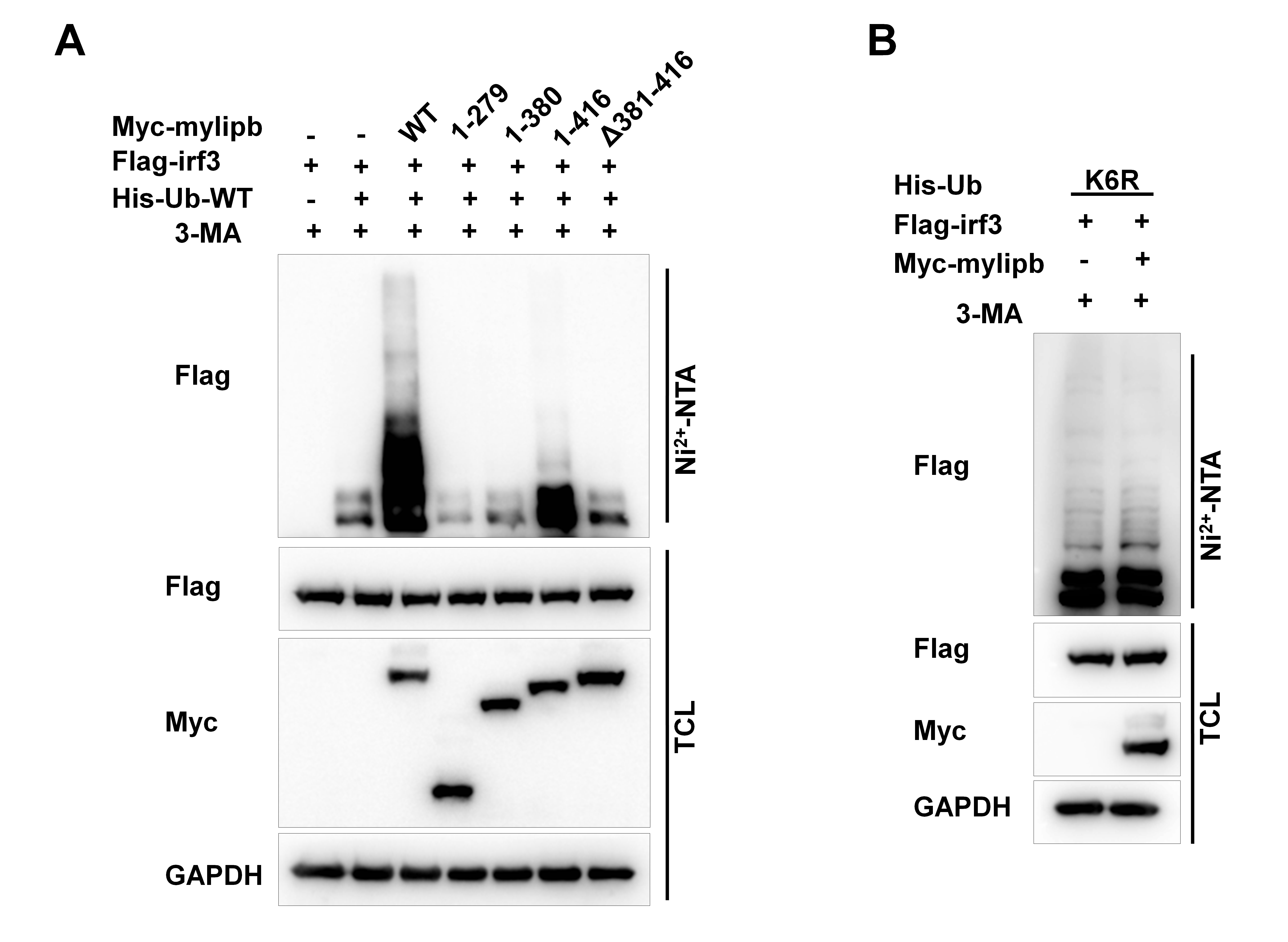

Supplement: S5 Fig — Related to Fig 5. (A) Mylipb promoted irf3 ubiquitination depended on ZF domain and ubiquitin ligase activity of mylipb. HEK 293T cells were transfected with Flag-irf3, Myc-mylipb-WT, Myc-mylipb-1-279, Myc-mylipb-1-380, Myc-mylipb-1-416, Myc-mylipb-Δ381–416, or empty vector, together with His-ubiquitin. At 24 h posttransfection, the cells were treated with 3-MA for 8 h. The cells were lysed using guanidinium chloride, and purifified with Ni2+-NTA agarose. (B) Mylipb promoted irf3 K6-linked ubiquitination. HEK293T cells were transfected with Flag-irf3, Myc-mylipb, or empty vector, together with His-K6R ubiquitin. At 24 h posttransfection, the cells were treated with 3-MA for 8 h. The cells were lysed using guanidinium chloride, and purifified with Ni2+-NTA agarose. (TIF) [file ppat.1012227.s005.tif]

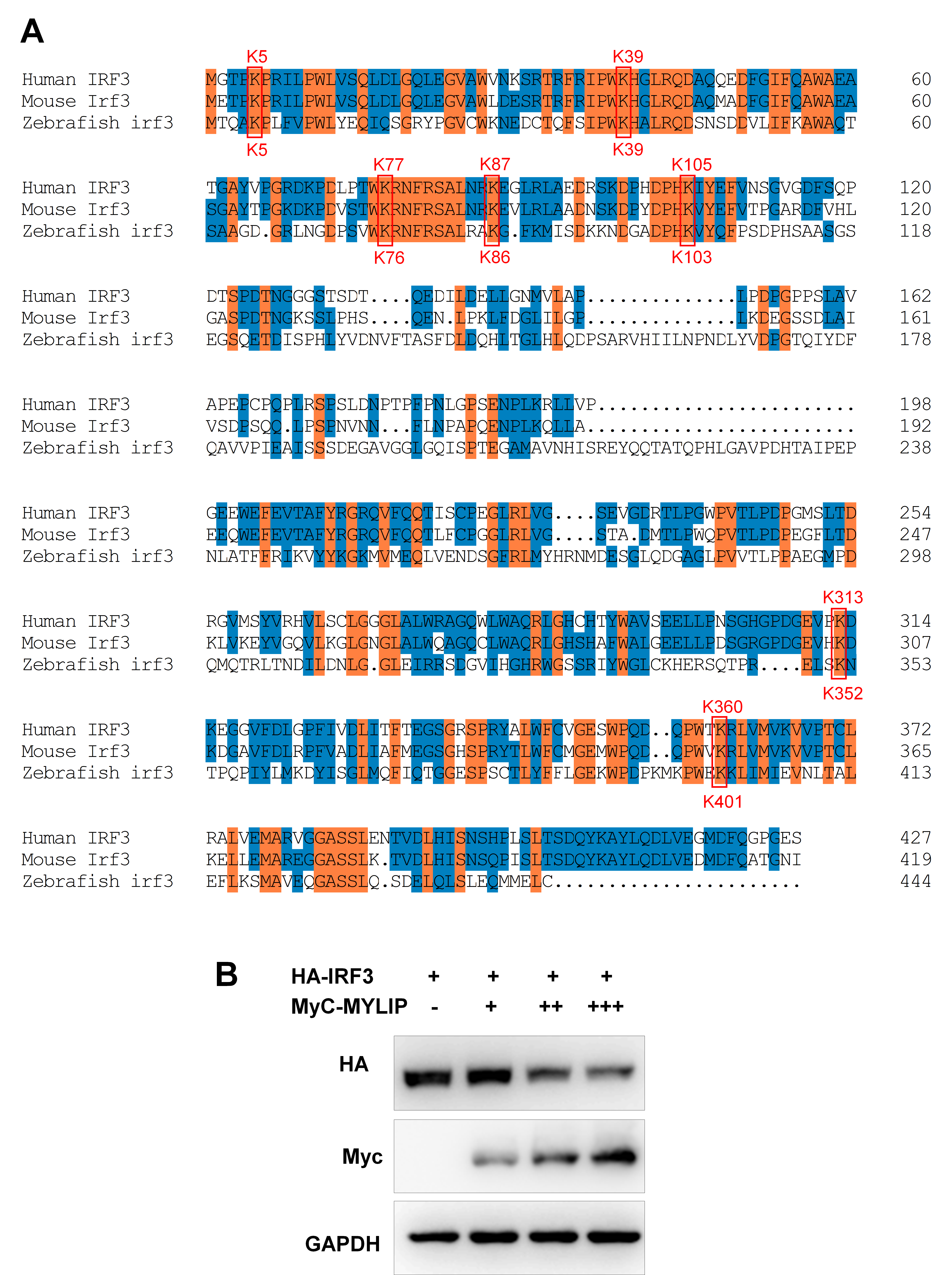

Supplement: S6 Fig — (A) Amino acid sequence alignment of human IRF3, mouse Irf3, zebrafish irf3. (B) MYLIP induced degradation of IRF3. HEK293T cells co-transfected with Myc-IRF3 and empty vector together with HA-IRF3 for 24 h. The lysates were then subjected to IB with the indicated Abs. (TIF) [file ppat.1012227.s006.tif]

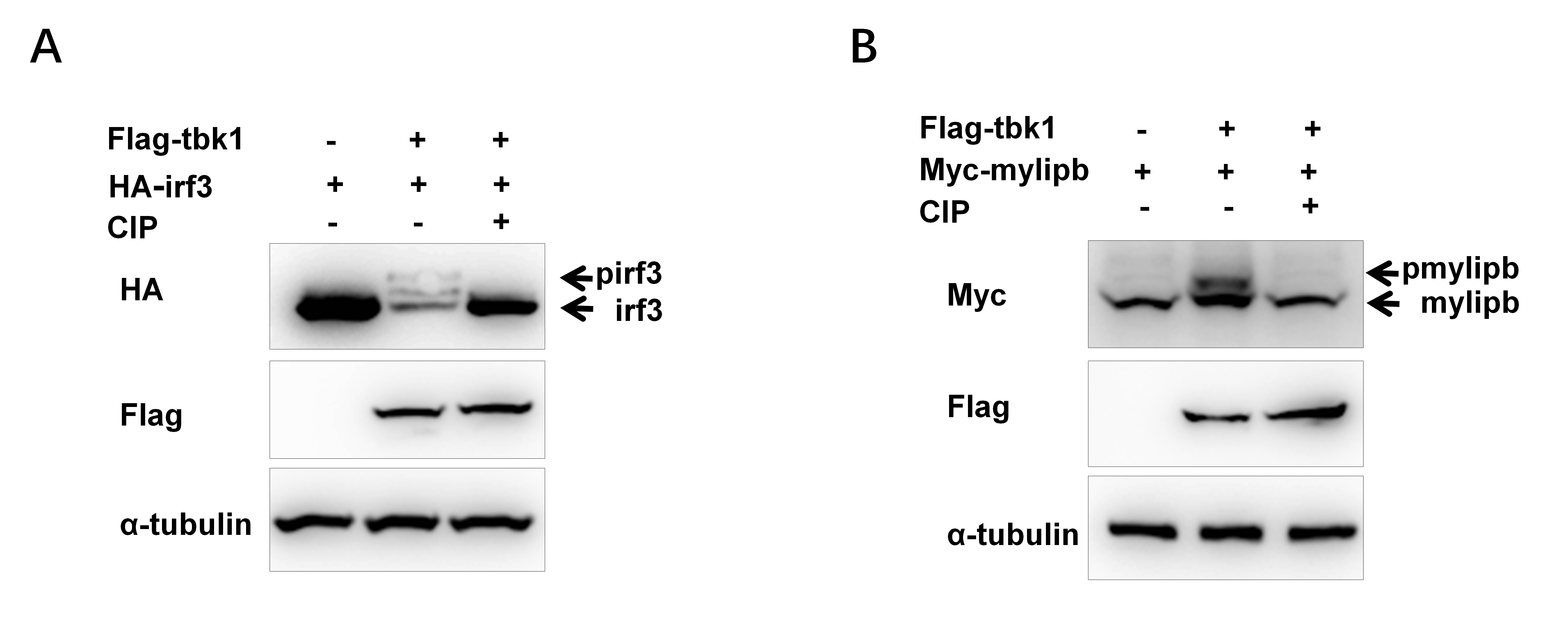

Supplement: S7 Fig — Related to Fig 7. (A-B) The amount of tbk1-phosphorylated irf3(A) and mylipb(B) were reduced by CIP treatment. Cells were transfected with the indicated plasmids (1 μg each) for 24 h. Then the cell lysates (100 μL) were treated with or without CIP (10 U) for 30 min at 37°C. Then the lysates were detected by IB with the indicated Abs. (TIF) [file ppat.1012227.s007.tif]

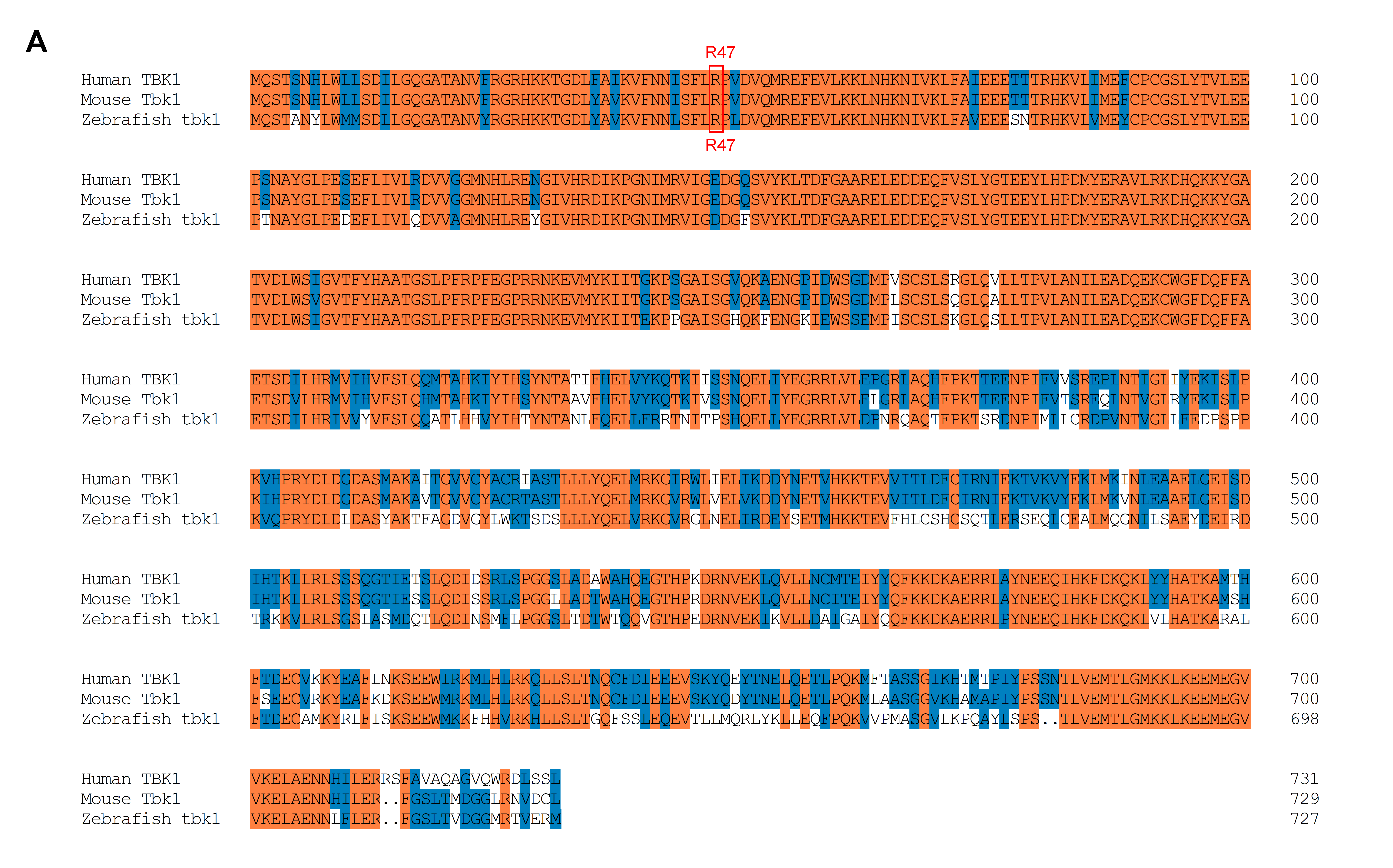

Supplement: S8 Fig — (A) Homology alignment analysis show that the corresponding zebrafish tbk1 for R47 of human TBK1 is R47. (TIF) [file ppat.1012227.s008.tif]

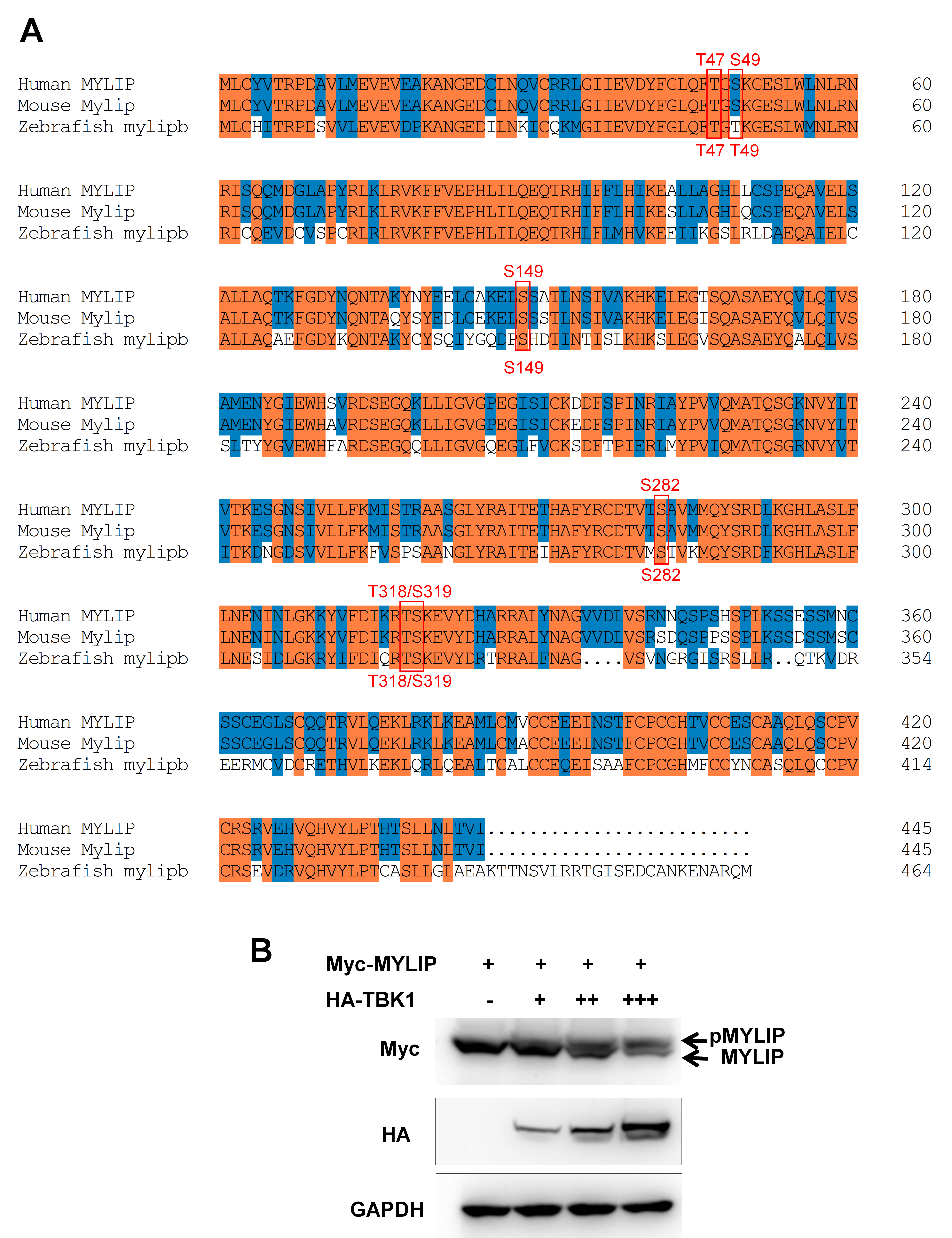

Supplement: S9 Fig — (A) Amino acid sequence alignment of human MYLIP, mouse Mylip, zebrafish mylipb. (B) TBK1 phosphorylates MYLIP. HEK293T cells co-transfected with HA-TBK1 and empty vector together with Myc-MYLIP for 24 h. The lysates were then subjected to IB with the indicated Abs. (TIF) [file ppat.1012227.s009.tif]

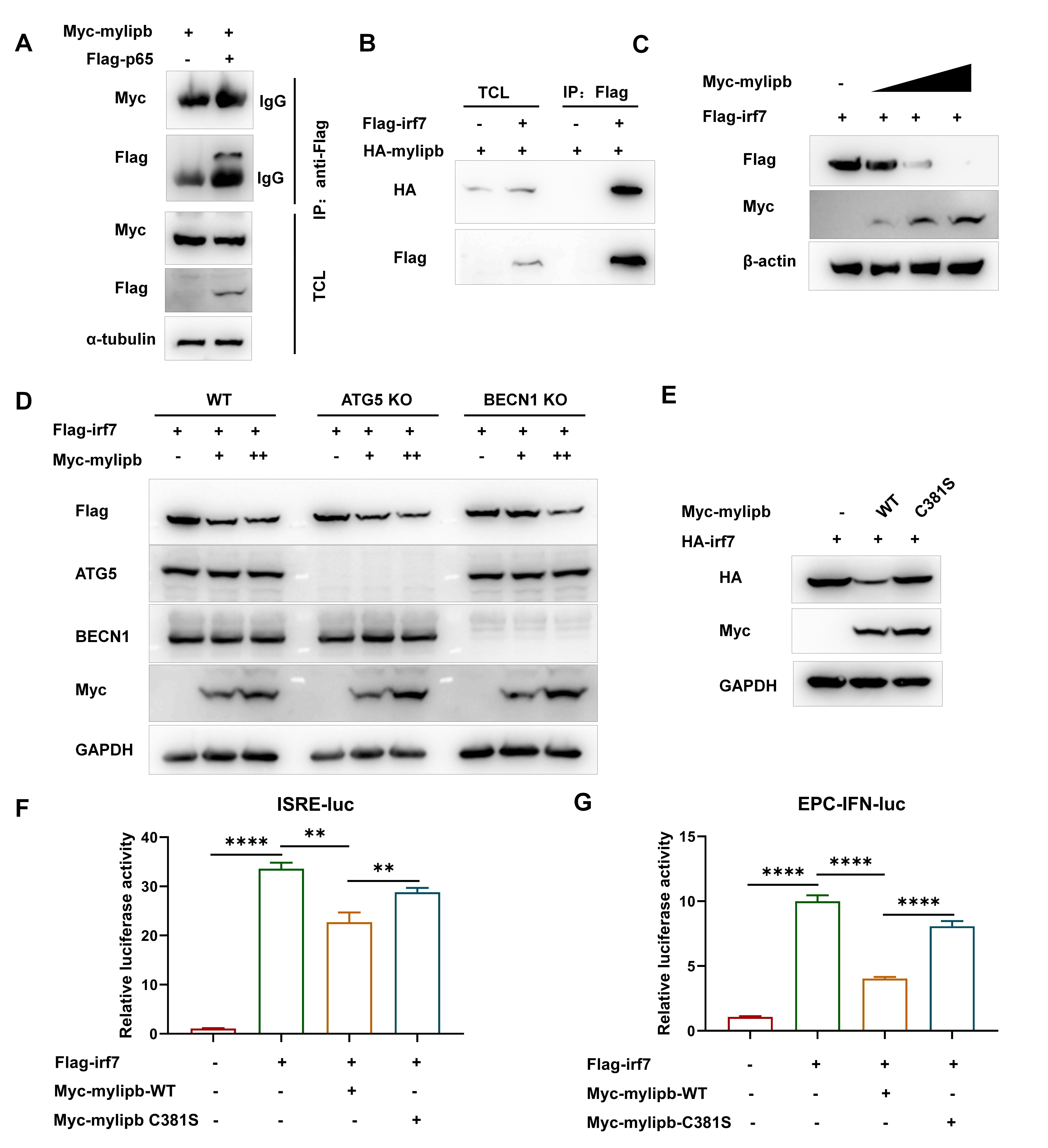

Supplement: S10 Fig — (A) Mylipb don’t associated with p65. HEK293T cells seeded in 100-mm dishes were transfected with the indicated plasmids (4 μg each). After 24 h, total cell lysates were immunoprecipitated (IP) with anti-Flag antibody conjugated agarose beads. Then, the immunoprecipitates and cell lysates were detected with anti-Myc or anti-Flag Ab, respectively. (B) Mylipb associated with irf7. HEK293T cells seeded in 100-mm dishes were transfected with the indicated plasmids (4 μg each). After 24 h, total cell lysates were immunoprecipitated (IP) with anti-Flag antibody conjugated agarose beads. Then, the immunoprecipitates and cell lysates were detected with anti-HA or anti-Flag Ab, respectively. (C) Mylipb induced the degradation of irf7 in a dose-dependent manner. HEK293T cells were transfected with Myc-empty, Myc-mylipb, and Flag-irf7 for 24 h, and then the cells were harvested to perform immunoblotting. (D) Wild-type (WT), ATG5 and BECN1 knockout (KO) 293T cells were co-transfected with Myc-mylipb and Flag-irf7 for 24 h. The cell lysates were subjected to western blotting with the indicated antibodies. (E) Mylipb induced the degradation of irf7 dependent of its ubiquitin ligase activity. HEK293T cells were transfected with Myc-mylipb-WT or Myc-mylipb-C381S, together with HA-irf7 for 24 h. The lysates were then subjected to IB with the indicated Abs. (F-G) Overexpression of mylipb-WT, but not mylipb-C381S suppressed the activity of ISRE reporter (F), EPC IFN reporter (G), induced by irf7. (TIF) [file ppat.1012227.s010.tif]
