# Supplementary material for: Zebrafish mylipb attenuates antiviral innate immunity through two synergistic mechanisms targeting transcription factor irf3
Source: PLoS Pathog. 2024 May 13;20(5):e1012227. doi: 10.1371/journal.ppat.1012227 (PMC11115282; doi:10.1371/journal.ppat.1012227)
Supplement: S1 Table — (DOCX) [file ppat.1012227.s011.docx]

**Table S1. The primer sequences**

| Primer | Sequence (5'-3') |
| --- | --- |
| zf-*mylipa*-RT-F | TGACGCCGCCTTCTGCCCCT |
| zf-*mylipa*-RT-R | GTCGTTAGTAGTGCAGGTAT |
| zf-*mylipb*-RT-F | CGCTCTGTGCTGCGAACAGG |
| zf-*mylipb*-RT-R | ATGCCAGTCCTTCTGAGCAC |
| zf-*ifn1*-RT-F | GAGCACATGAACTCGGTGAA |
| zf-*ifn1*-RT-R | TGCGTATCTTGCCACACATT |
| zf-*mxc*-RT-F | GAGGCTTCACTTGGCAACTC |
| zf-*mxc*-RT-R | TTGTTCCAATAAGGCCAAGC |
| zf*-lta*-RT-F | AAGCCAAACGAAGGTCA |
| zf-*lta*-RT-R | AACCCATTTCAGCGATTGTC |
| zf-*tnfα*-RT-F | GCTGGATCTTCAAAGTCGGGTGTA |
| zf-*tnfα*-RT-R | TGTGAGTCTCAGCACACTTCCATC |
| zf-*il-1β*-RT-F | TGGACTTCGCAGCACAAAATG |
| zf-*il-1β*-RT-R | GTTCACTTCACGCTCTTGGATG |
| zf-*il8*-RT-F | GAATGAGCTTGAGAGGTCTGG |
| zf-*il8*-RT-R | GATCTTCTTAACCCATGGAGCA |
| zf-*β-actin* -RT-F | TACAATGAGCTCCGTGTTGC |
| zf-*β-actin* -RT-R | ACATACATGGCAGGGGTGTT |
| EPC-*ifn*-RT-F | ATGAAAACTCAAATGTGGACGTA |
| EPC-*ifn*-RT-R | GATAGTTTCCACCCATTTCCTTAA |
| EPC-*isg15*-RT-F | CAGCCTTGAGGATGATTCCAG |
| EPC-*isg15*-RT-R | TGCCGTTGTAAATCAGTCG |
| EPC-*viperin*-RT-F | AGCGAGGCTTACGACTTCTG |
| EPC-*viperin*-RT-R | GCACCAACTCTCCCAGAAAA |
| EPC-*tnfα*-RT-F | GCTTCACGCTCAACAAGTCTCA |
| EPC-*tnfα*-RT-R | GAAAGCTTGGTCCTGGTTCAC |
| EPC-*il-1β*-RT-F | CGGTGAAGTGCAACGTTTGTGA |
| EPC-*il-1β*-RT-R | TTGACGCATACAGCGACAGACT |
| EPC-*il8*-RT-F | GGAATGAGTCTTAGAGGTGTGG |
| EPC-*il8*-RT-R | CTTTGATCCCTTTAGGGTGGC |
| EPC-*β-actin*-RT-F | CACTGTGCCCATCTACGAG |
| EPC-*β-actin*-RT-R | CCATCTCCTGCTCGAAGTC |
| SVCV-N-protein-RT-F | TGAGGTGAGTGCTGAGGATG |
| SVCV-N-protein-RT-R | CCATCAGCAAAGTCCGGTAT |
| SVCV-P-protein-RT-F | TTGGACCTGGGATAGTGA |
| SVCV-P-protein-RT-R | CTTGCTTGGTTTGTGGG |
| SVCV-G-protein-RT-F | CGACCTGGATTAGACTTG |
| SVCV-G-protein-RT-R | AATGTTCCGTTTCTCACT |
| *mylipb*-null identification-F | CTGATGCATGTGAAGGAGGA |
| *mylipb*-null identification-R | GTAACGCAACTGATGCTAGG |
