# Supplementary material for: Zebrafish mylipb attenuates antiviral innate immunity through two synergistic mechanisms targeting transcription factor irf3
Source: PLoS Pathog. 2024 May 13;20(5):e1012227. doi: 10.1371/journal.ppat.1012227 (PMC11115282; doi:10.1371/journal.ppat.1012227)

Figure 2K

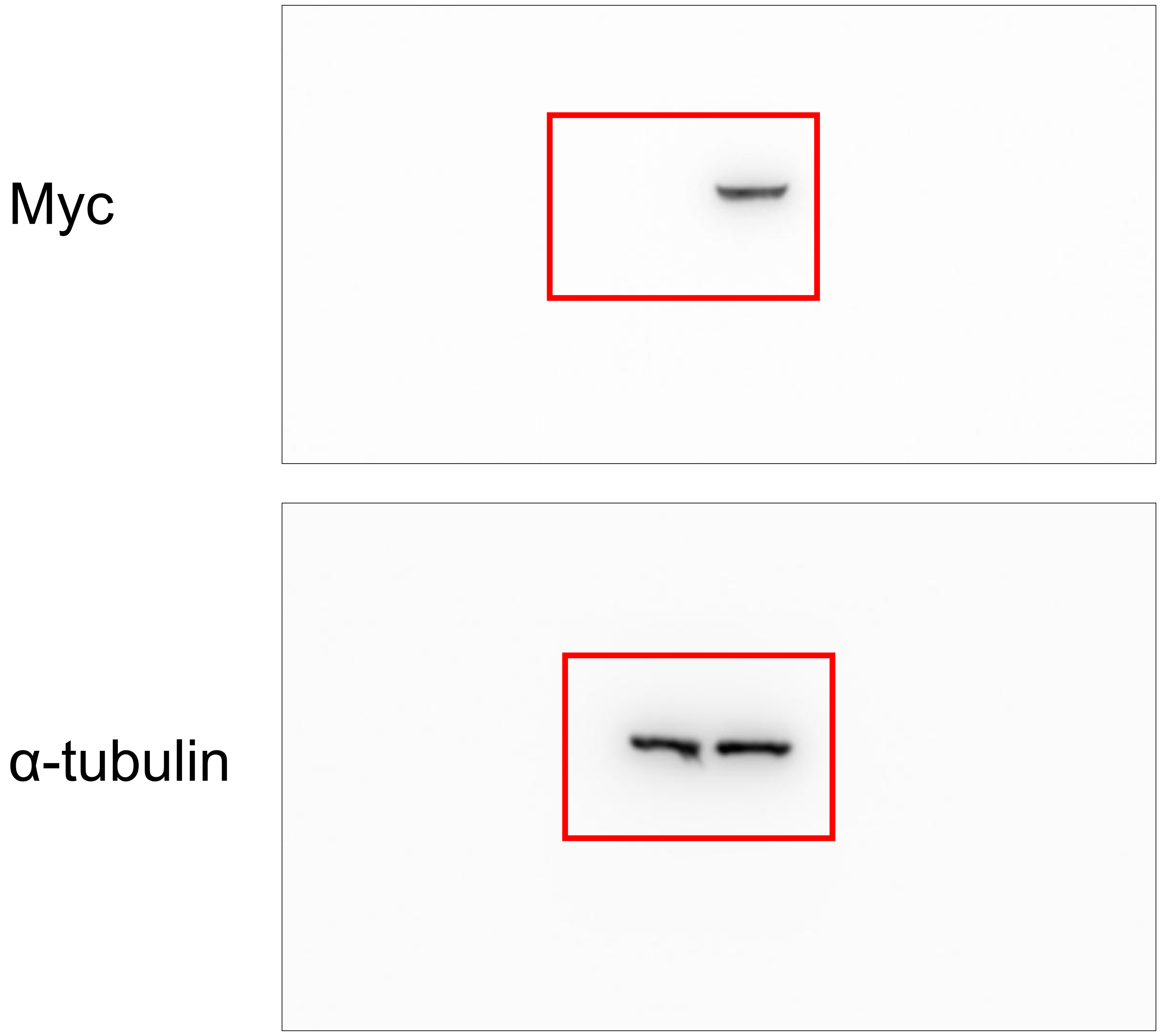

Figure 3A

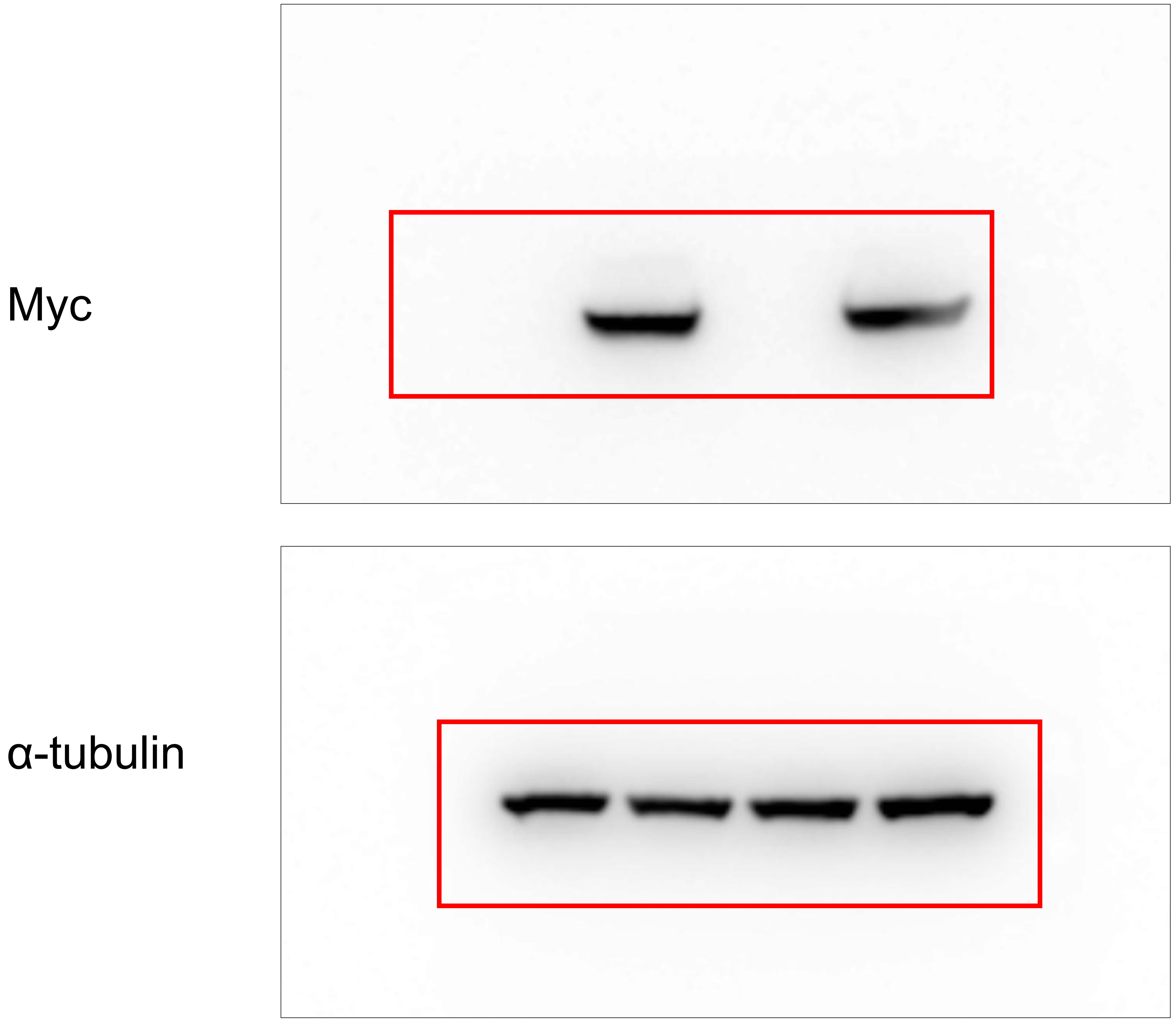

Figure 3B

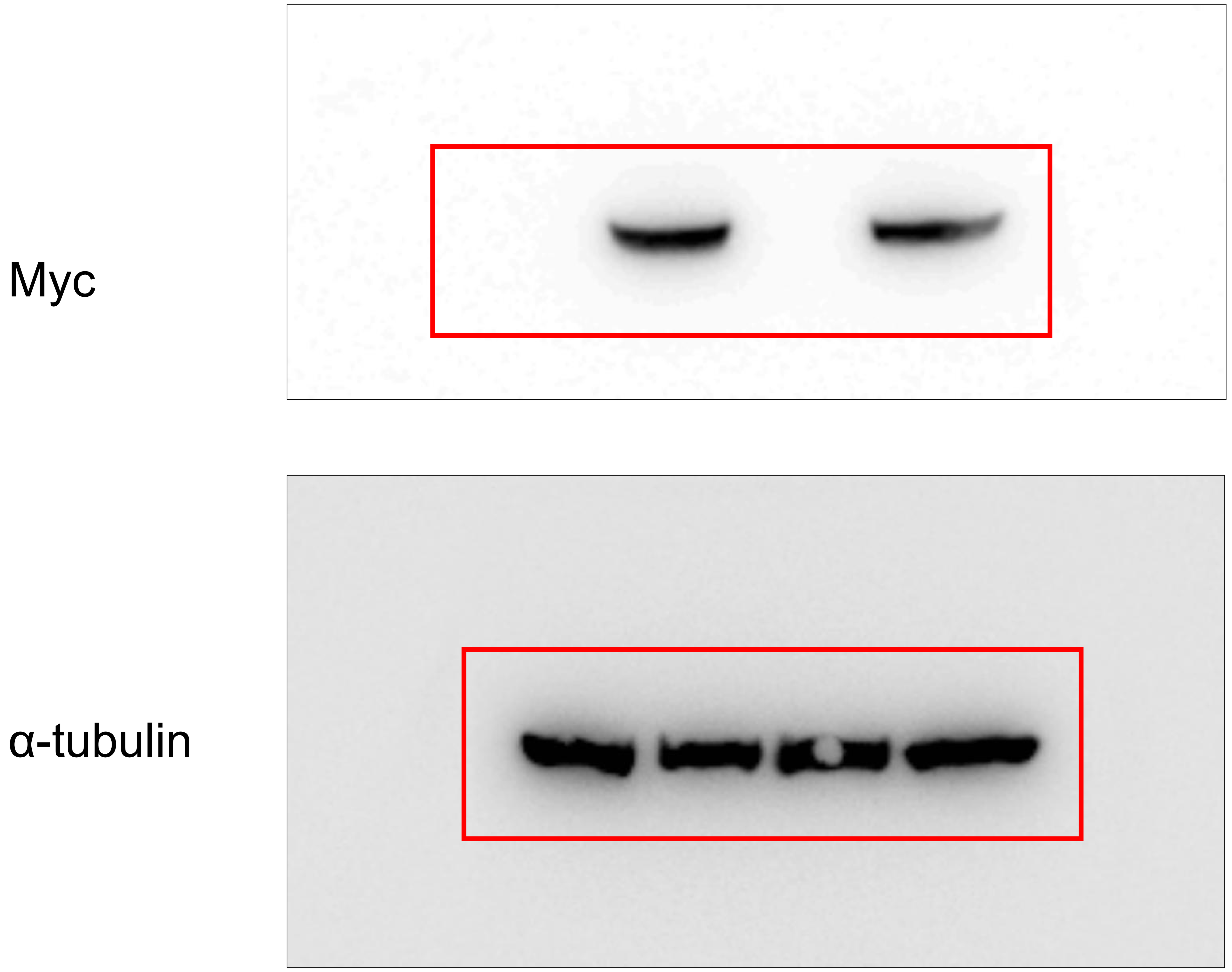

Figure 3C

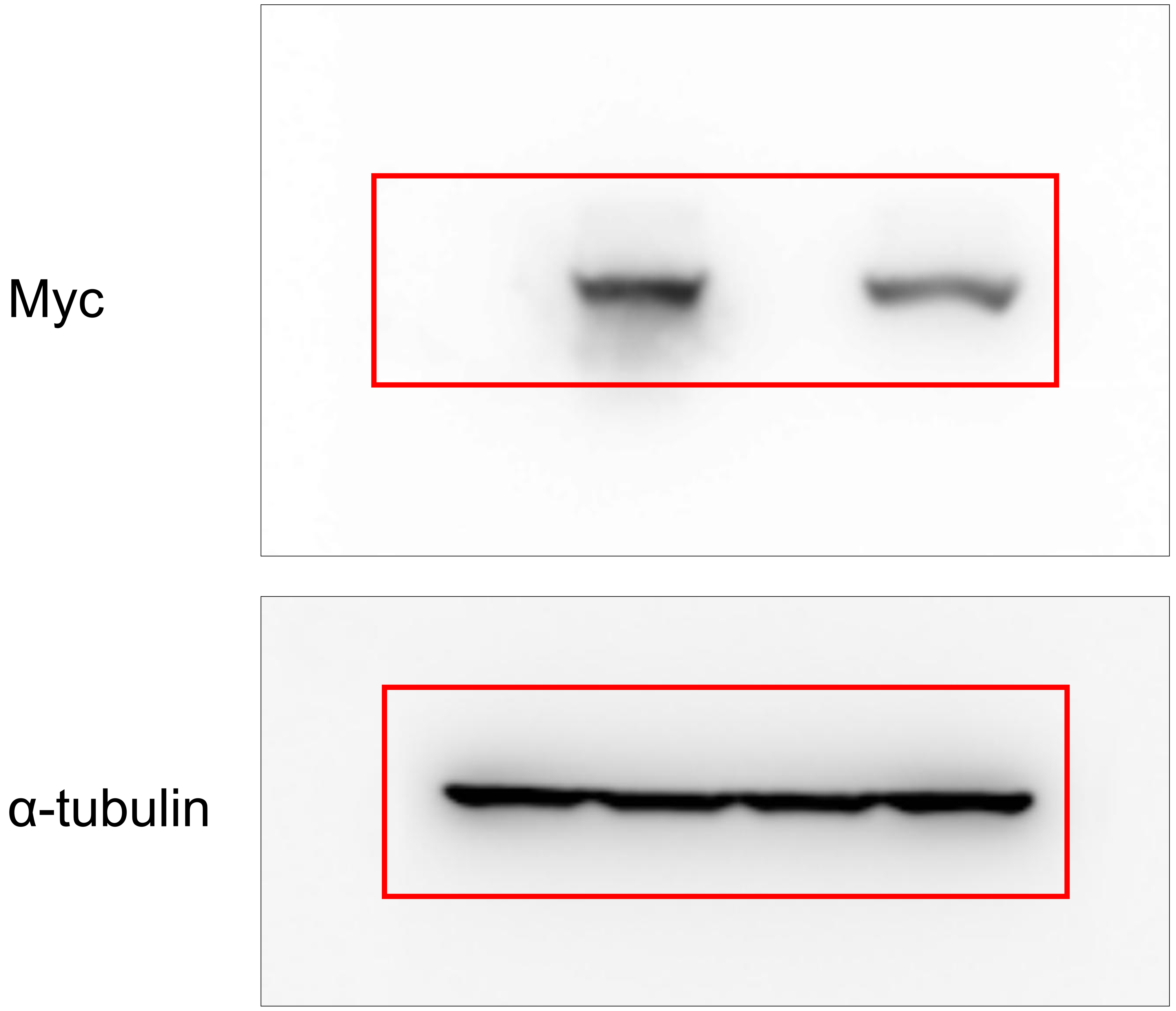

Figure 3D

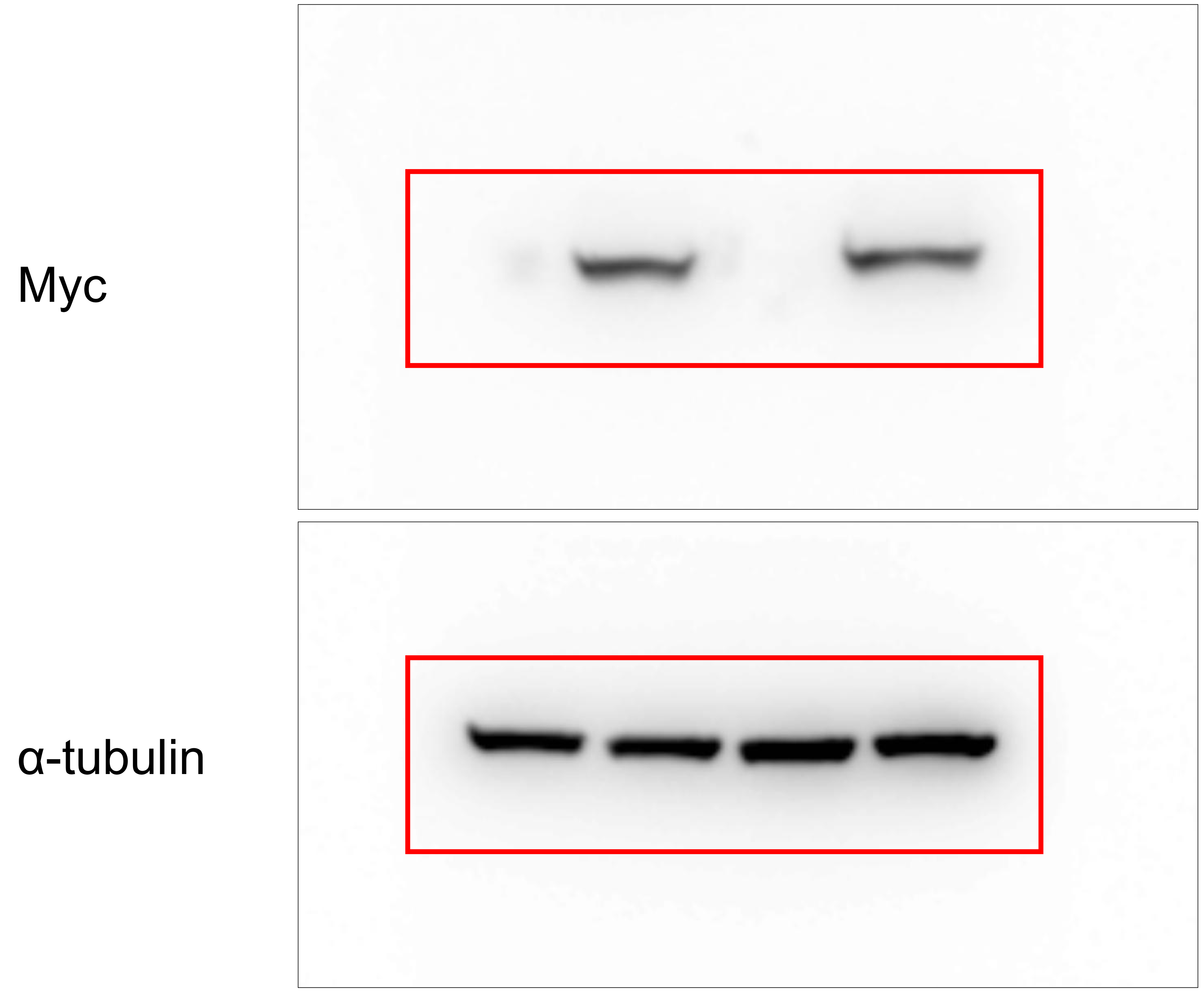

Figure 3E

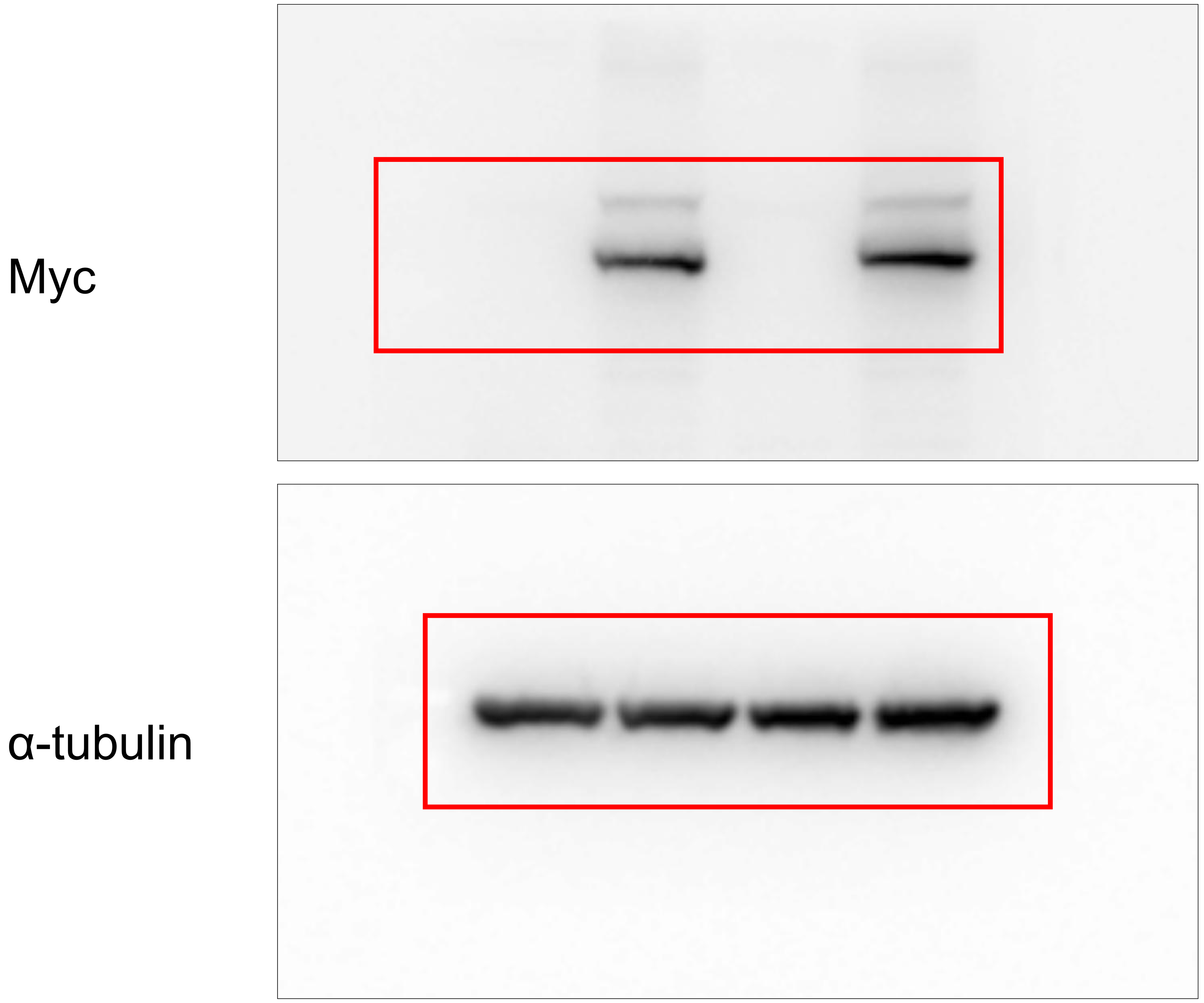

Figure 3F

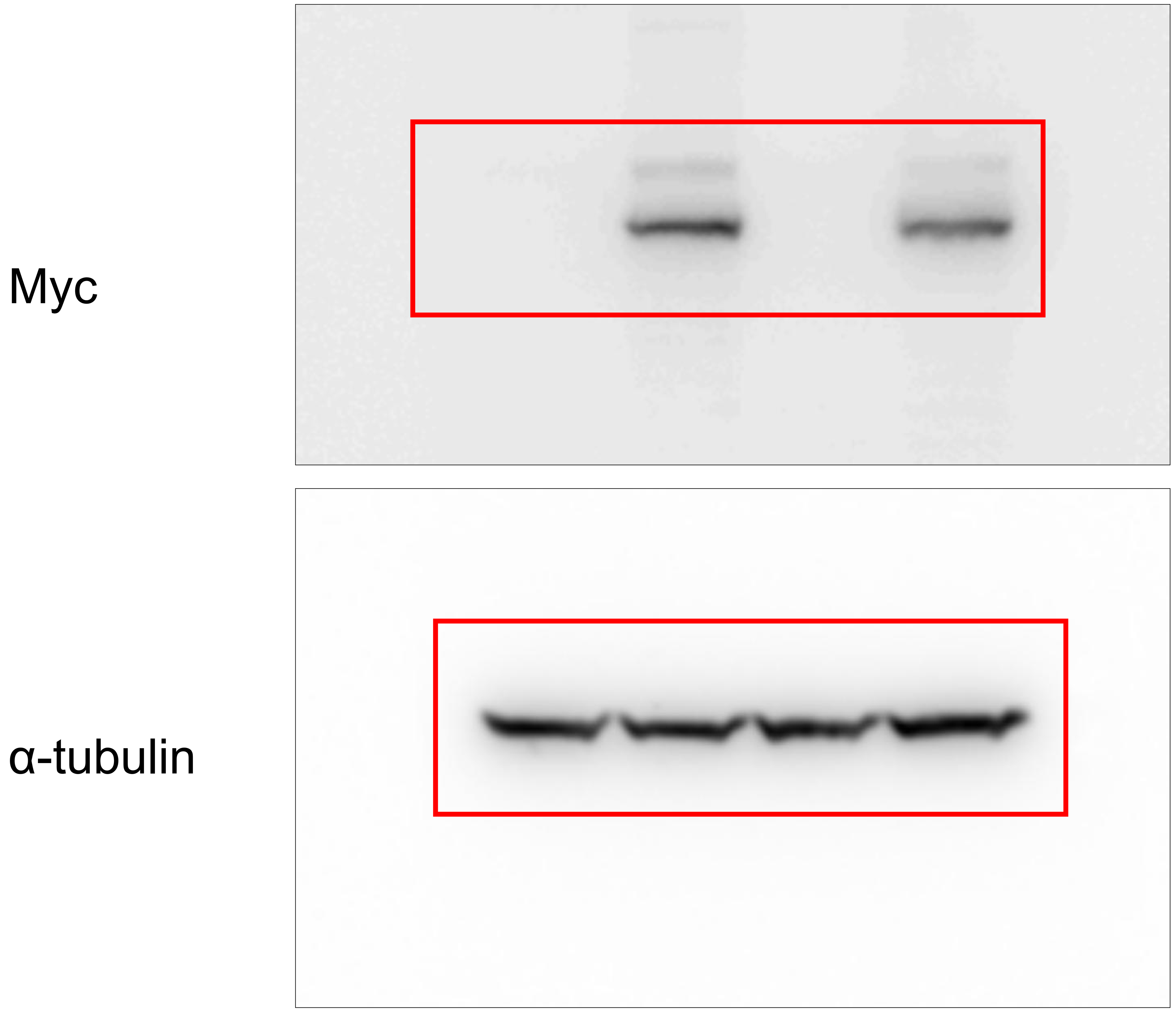

Figure 4D

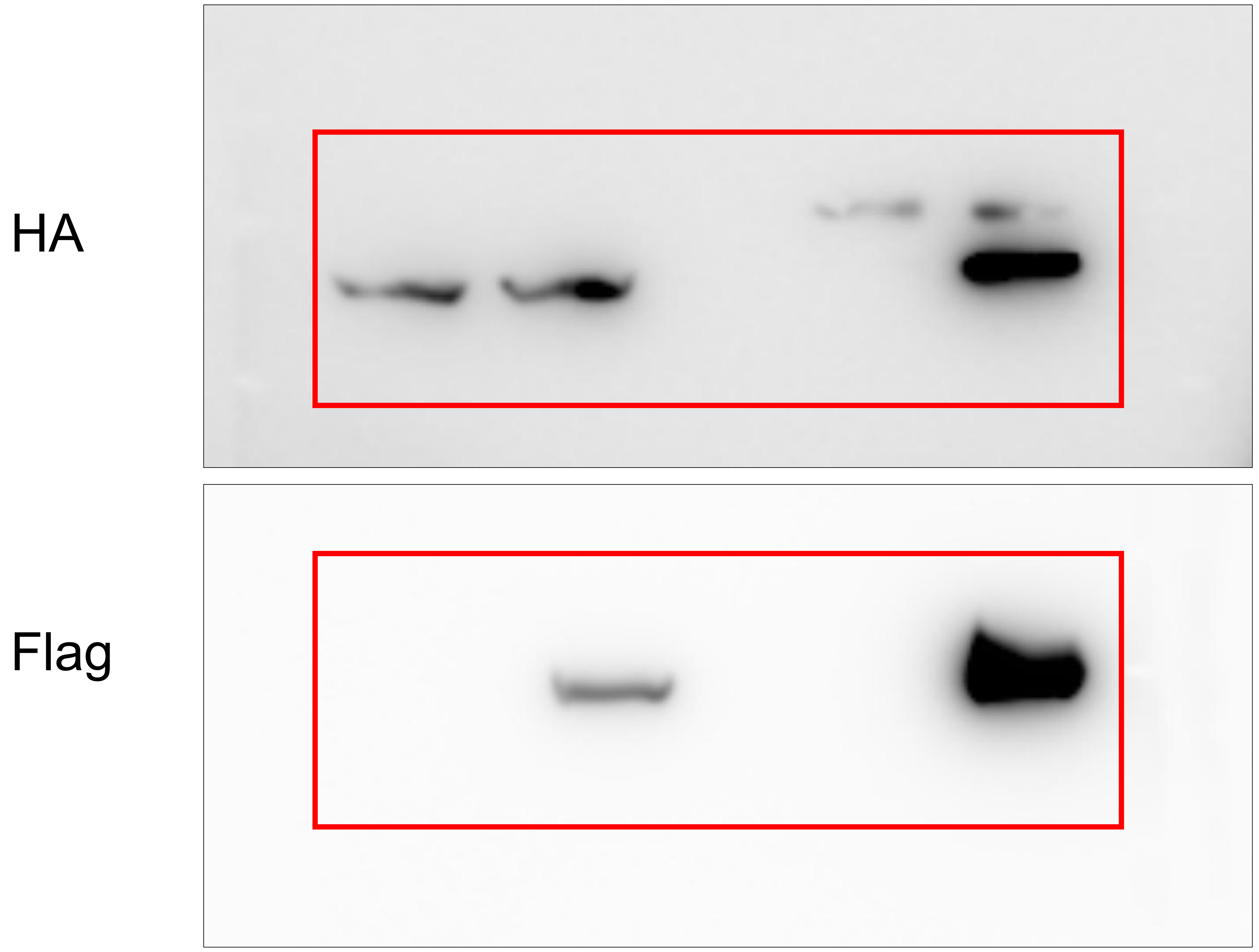

Figure 4E

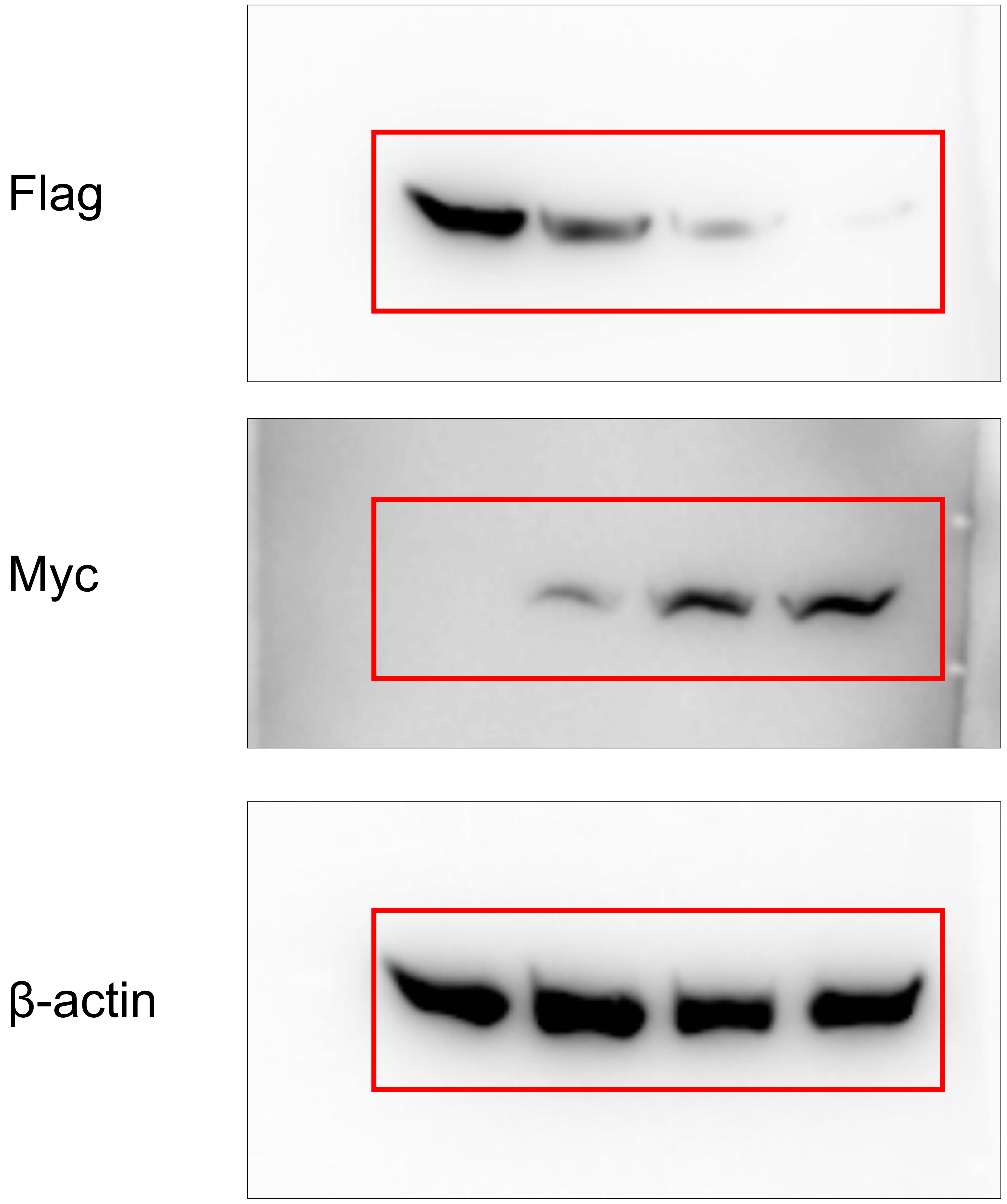

Figure 4F

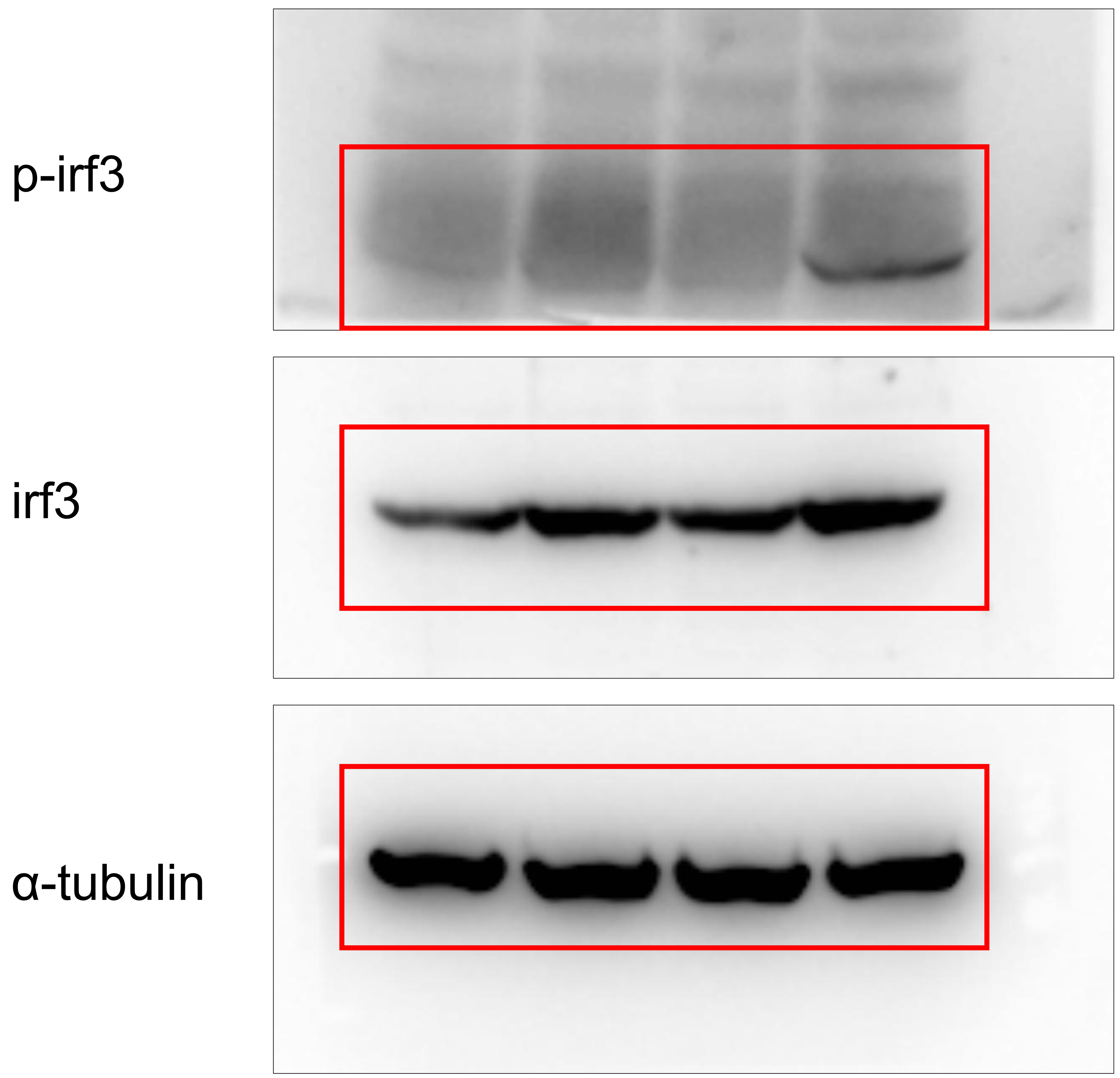

Figure 4G

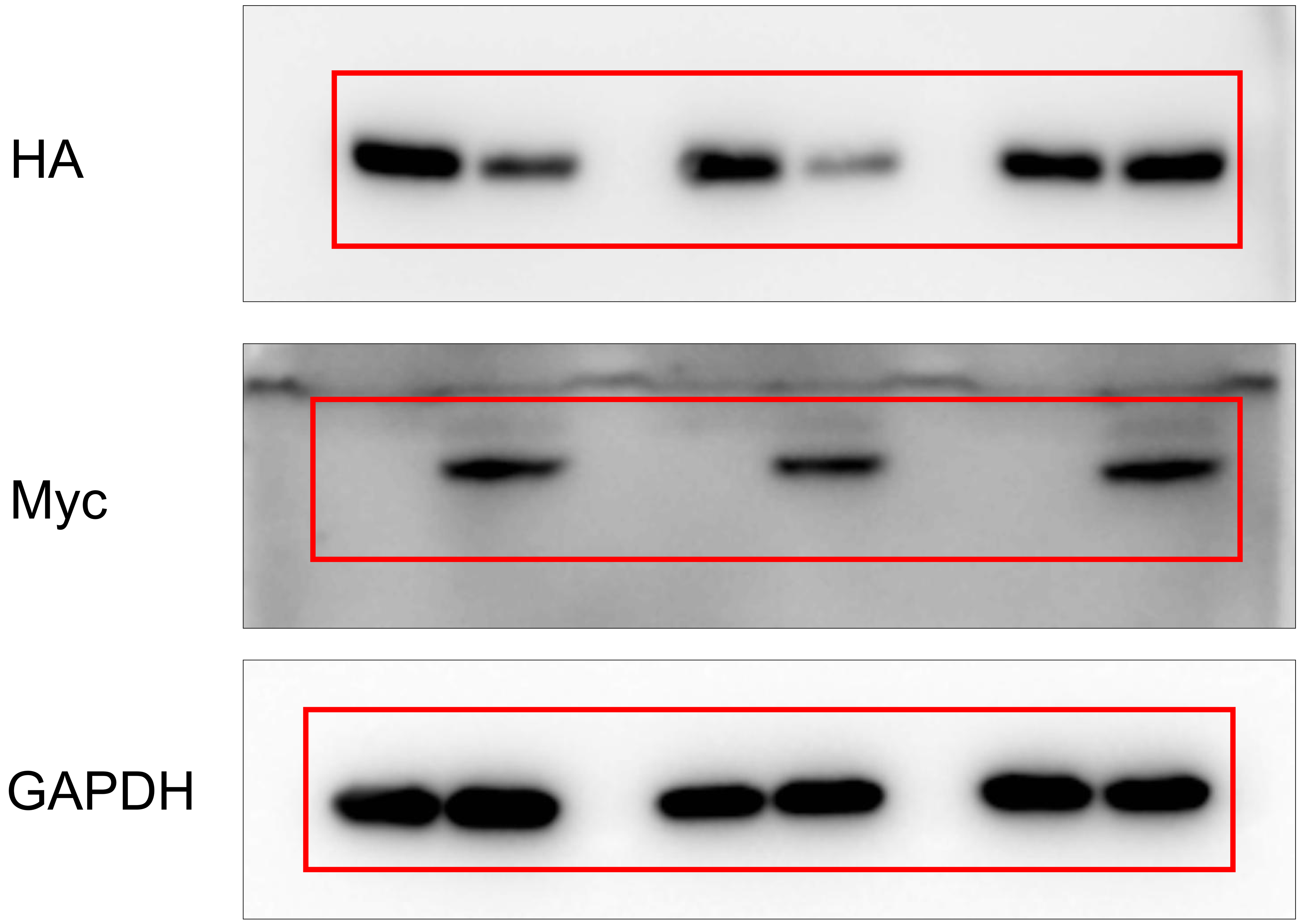

Figure 4H

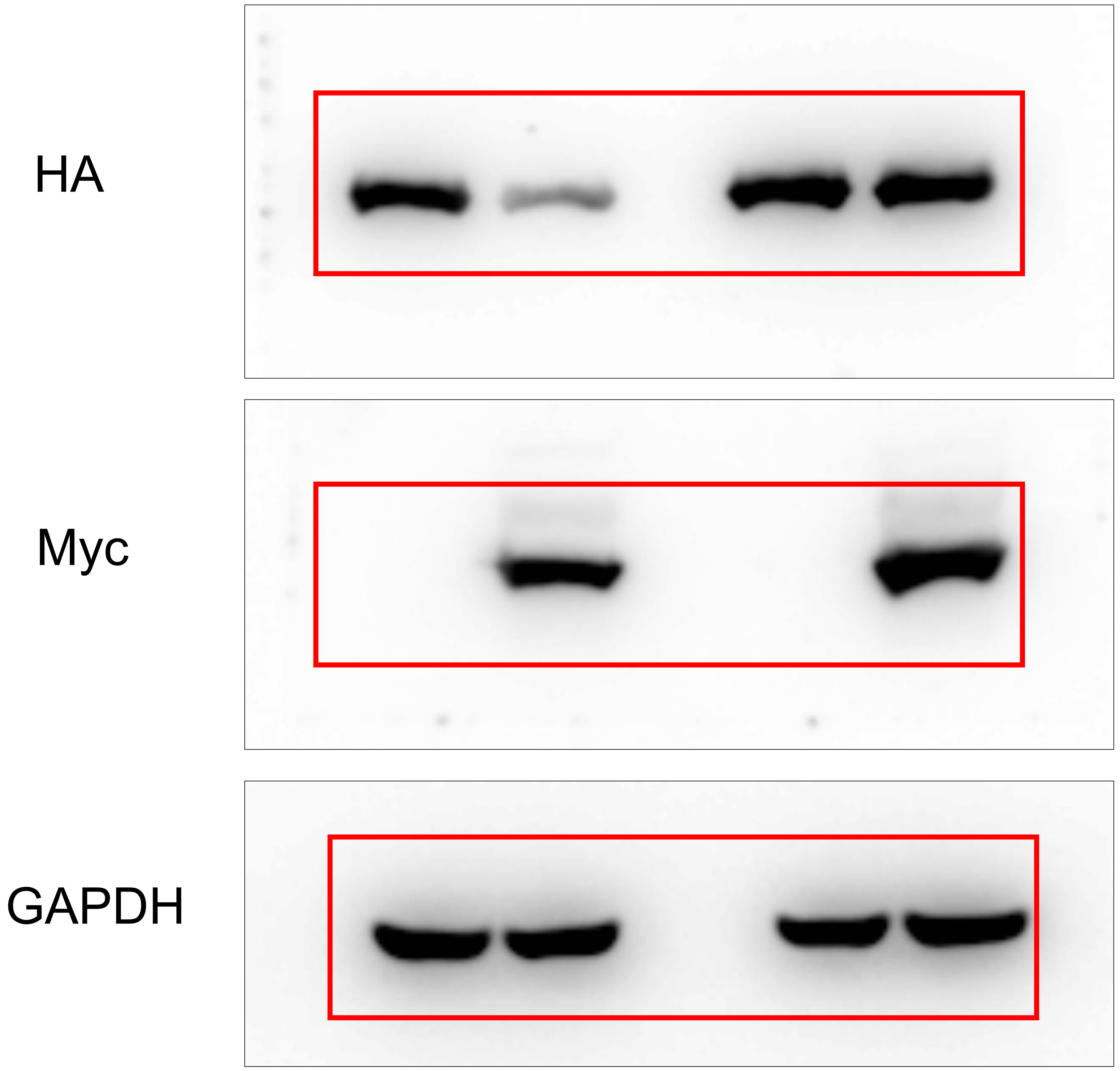

Figure 4I

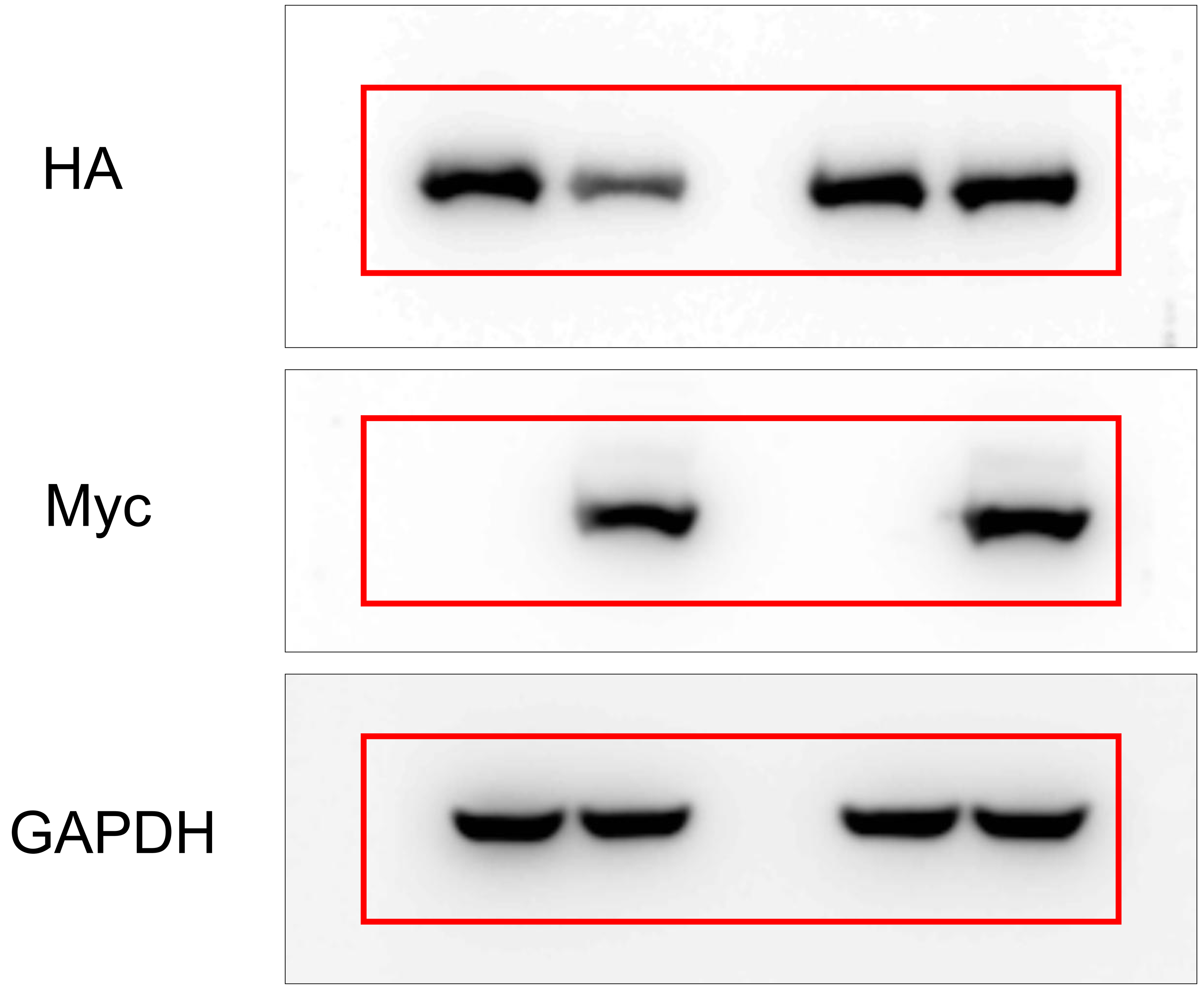

Figure 4J

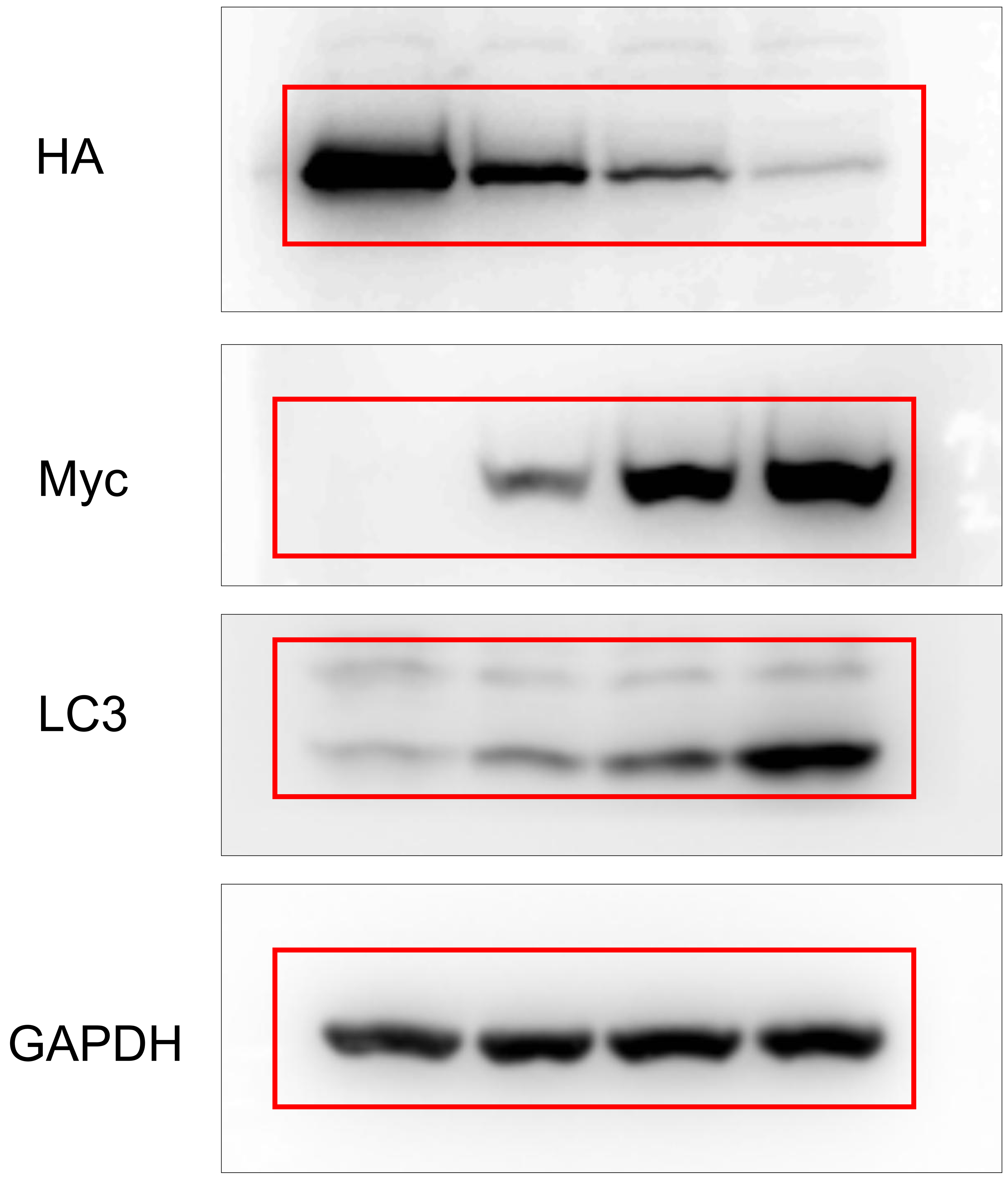

Figure 5B

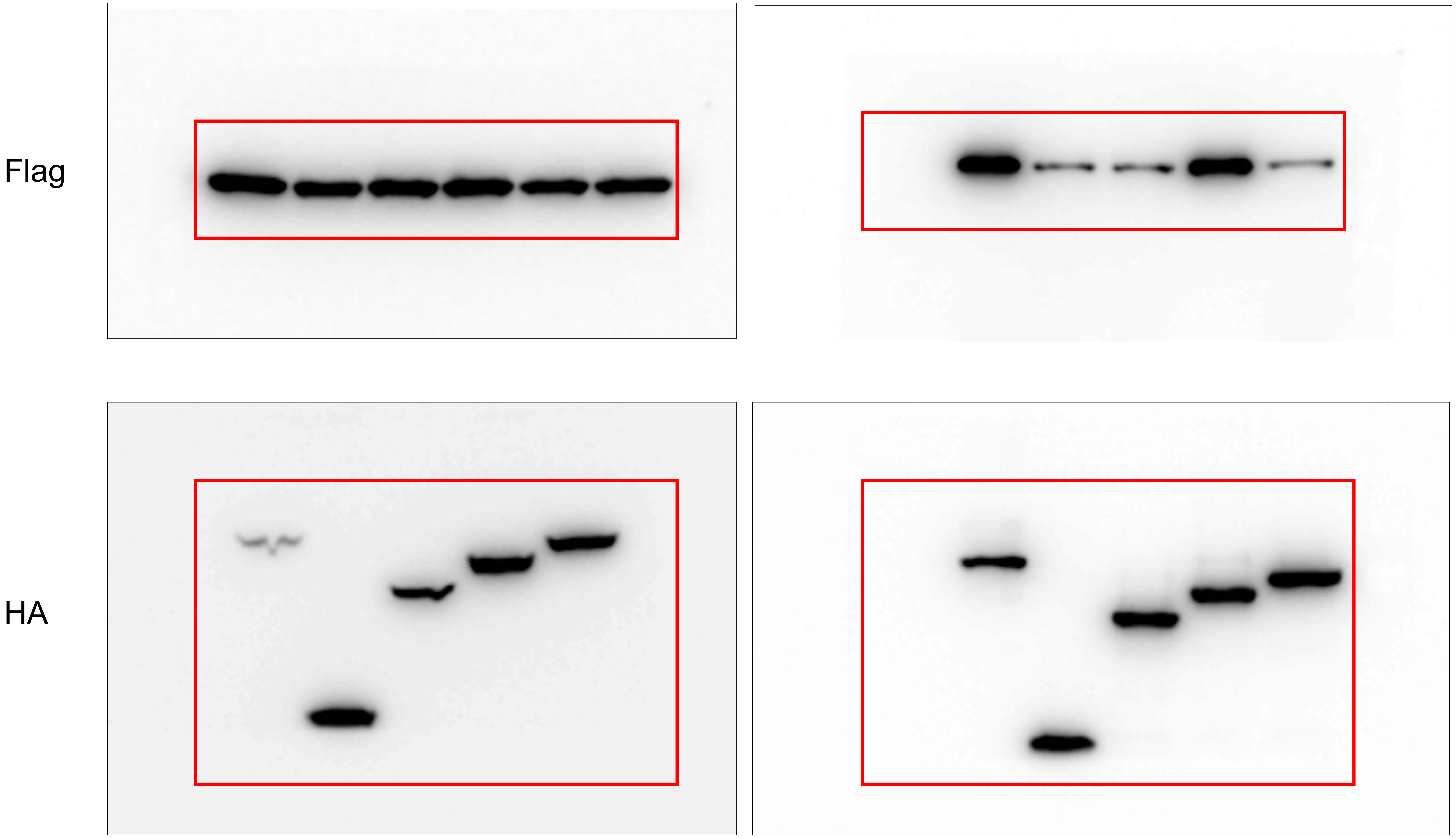

Figure 5C

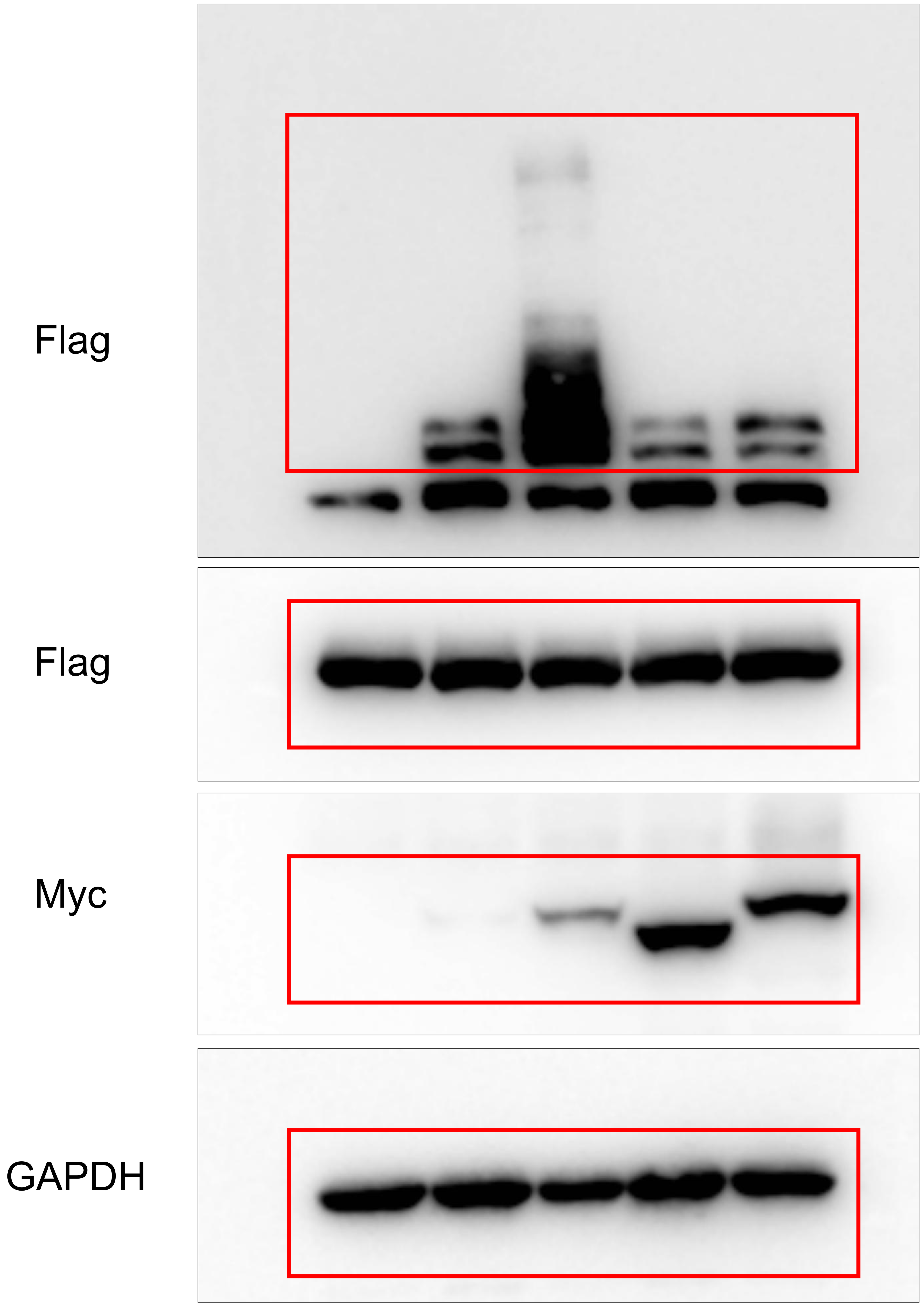

Figure 5D

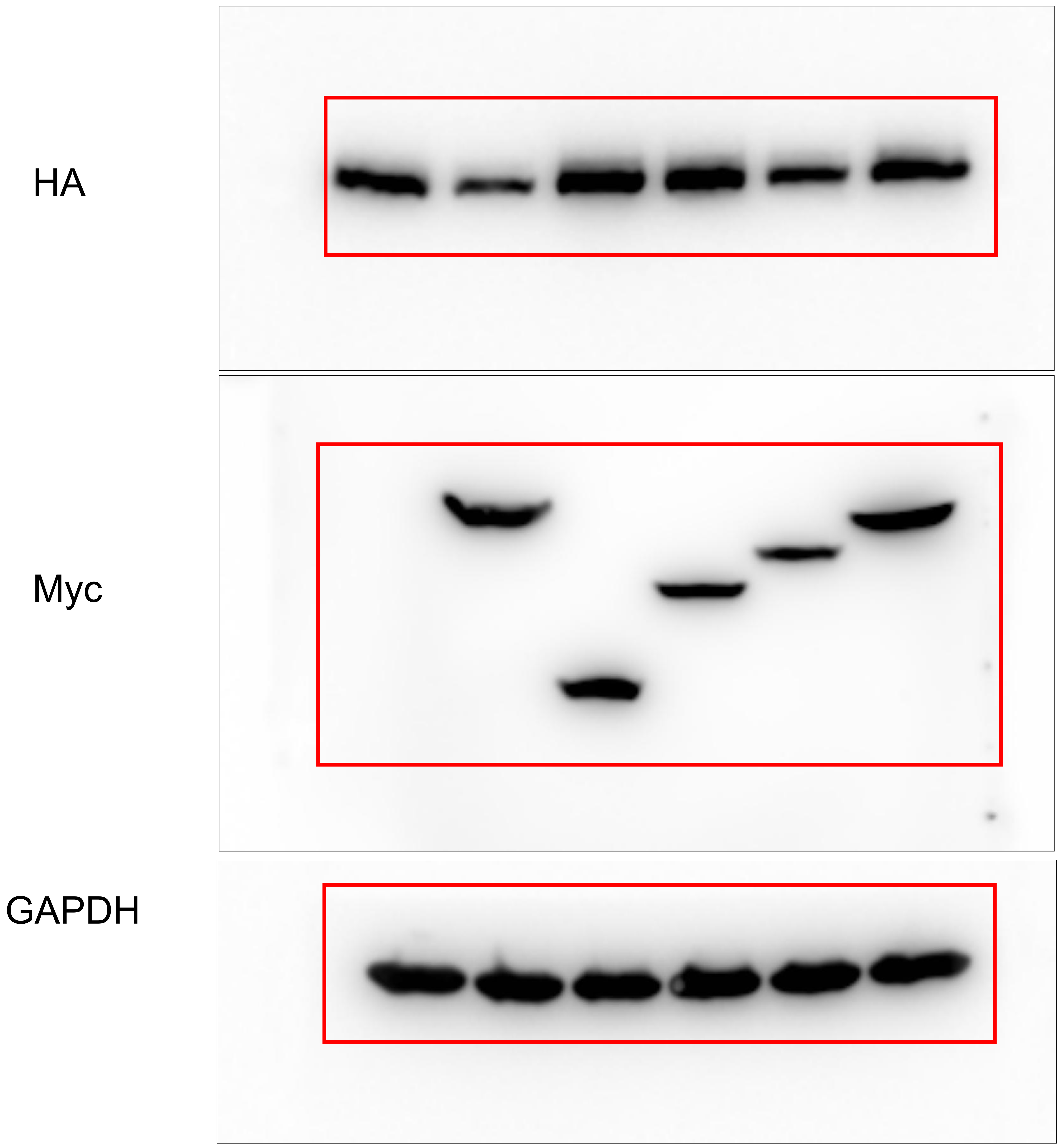

Figure 5E

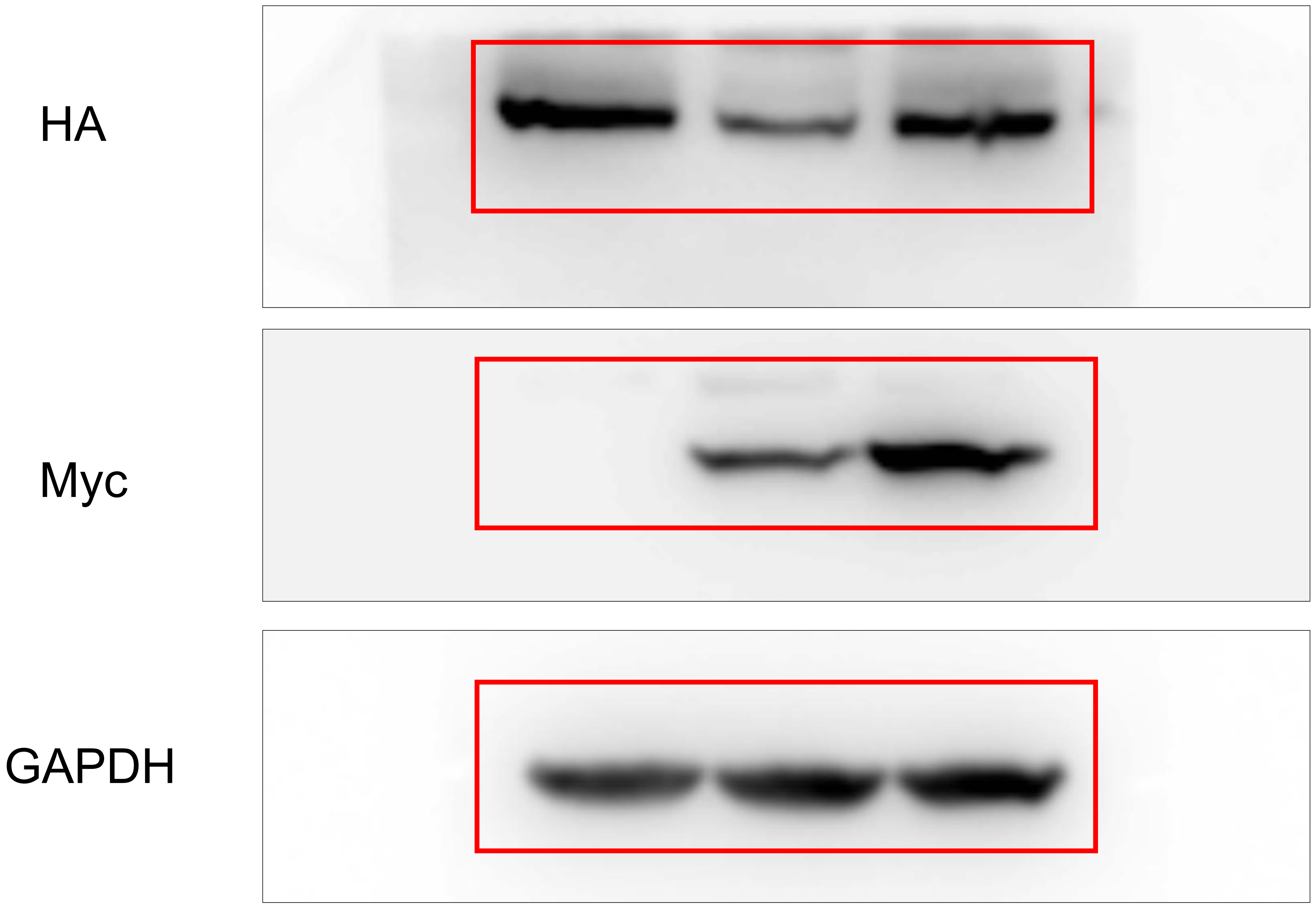

Figure 5F

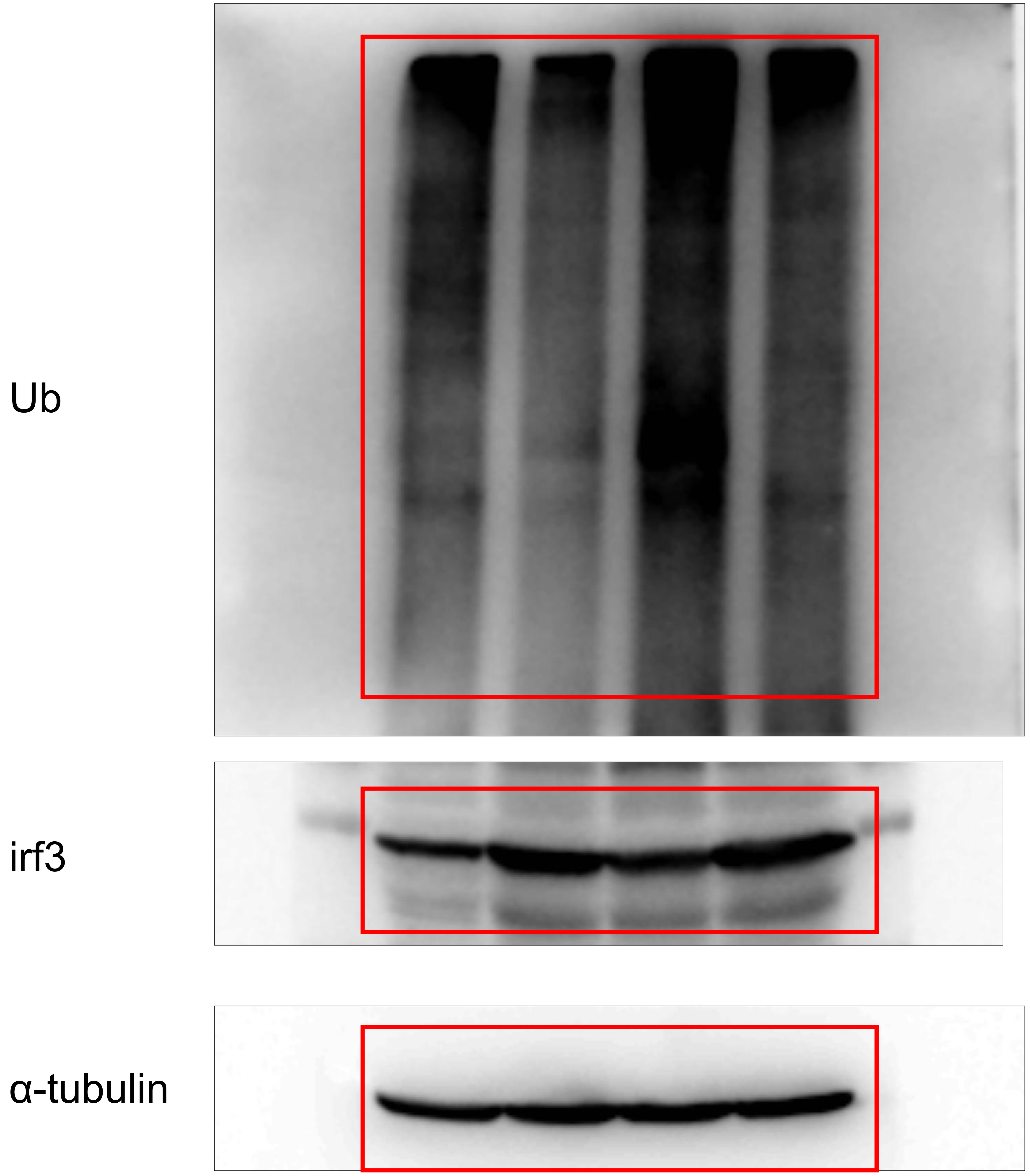

Figure 5G

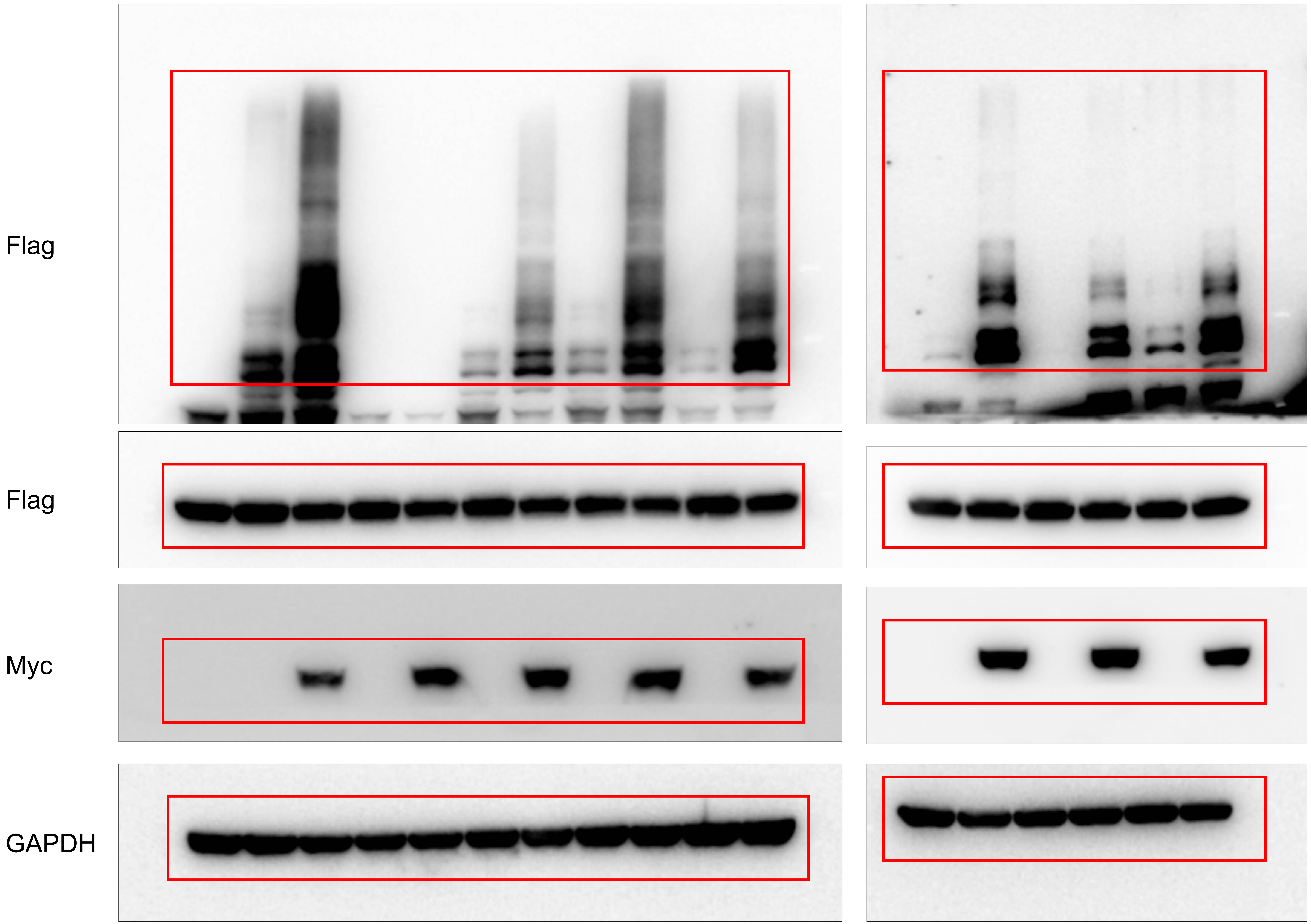

Figure 5H

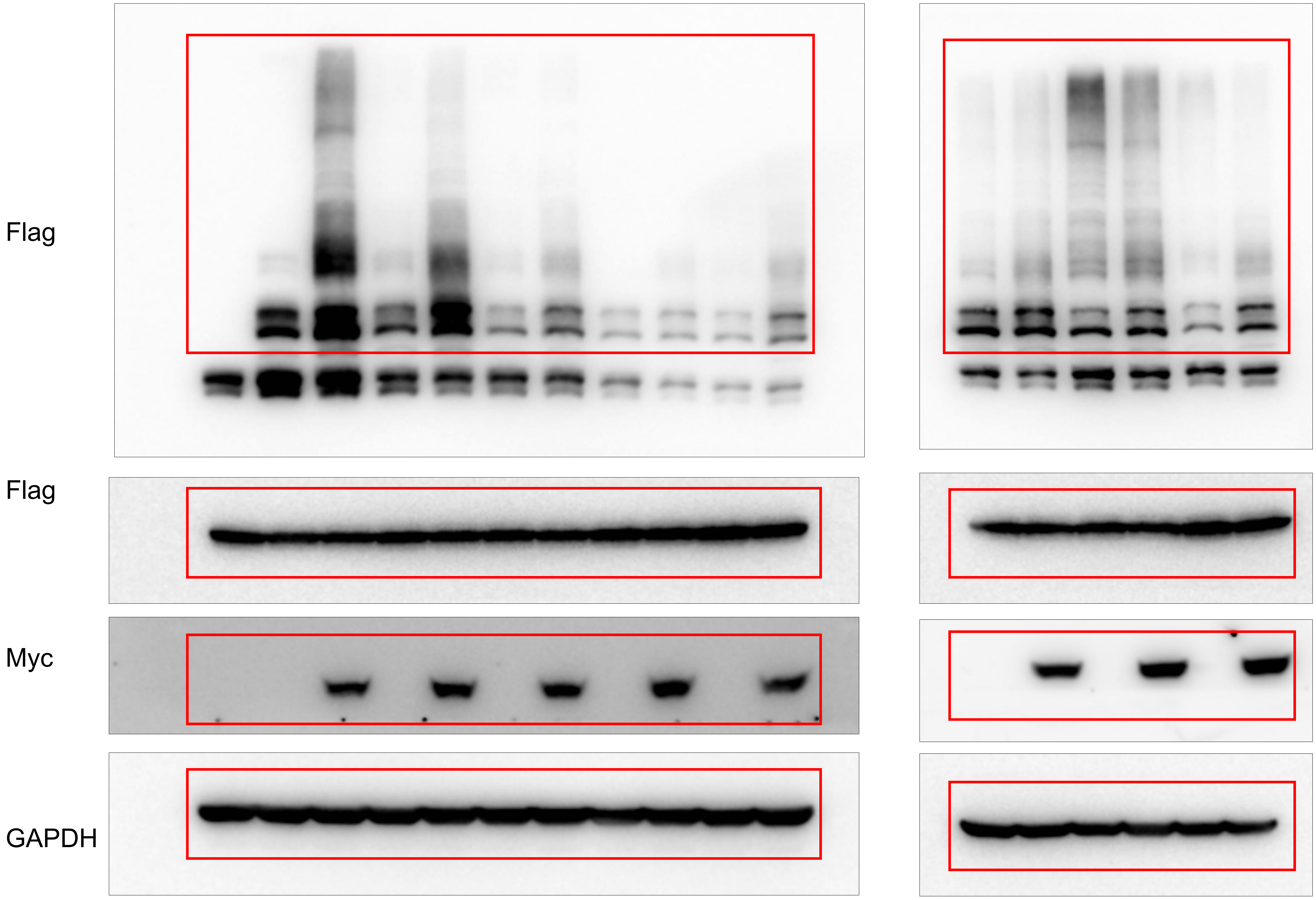

Figure 5I

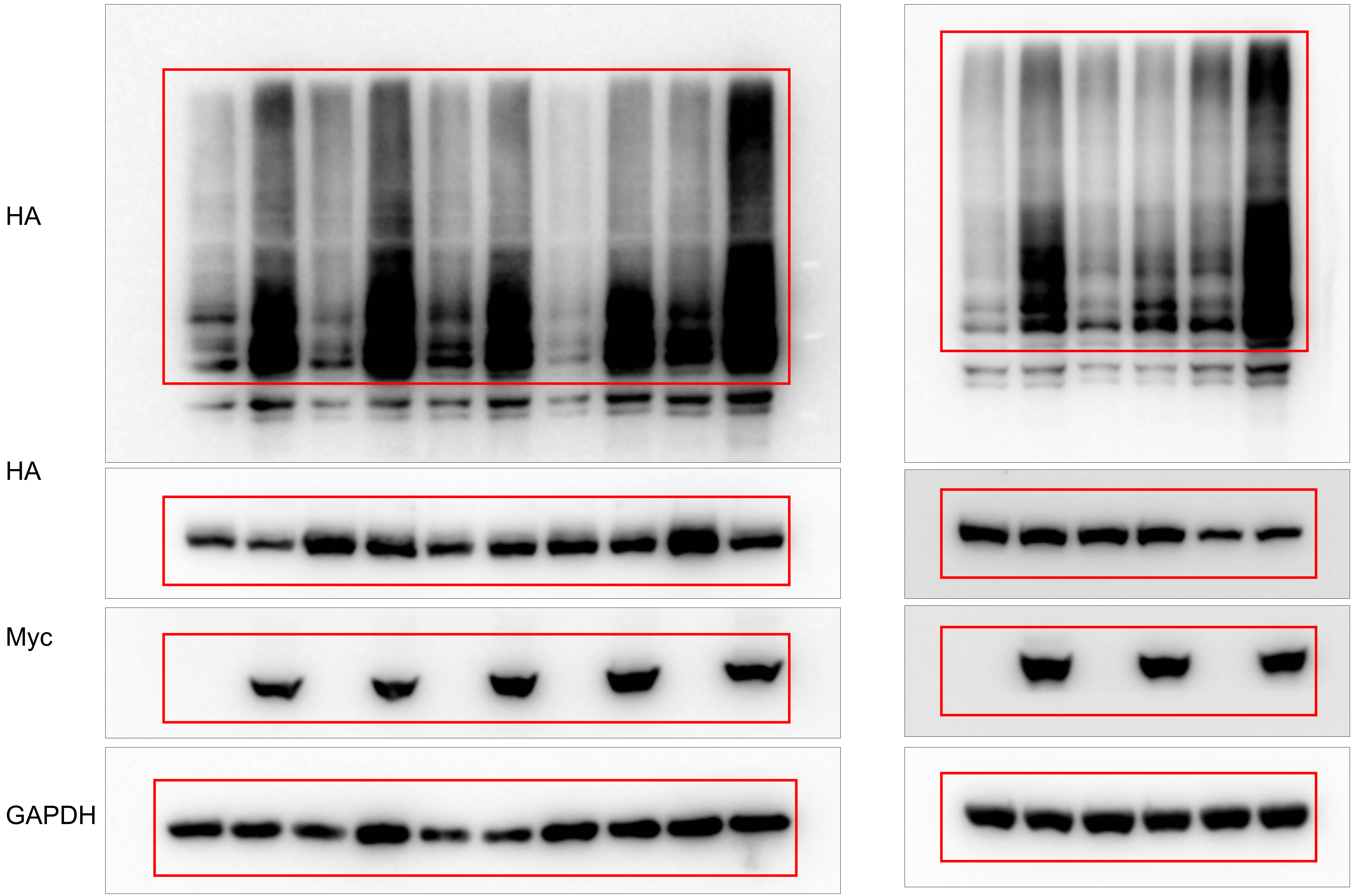

Figure 5K

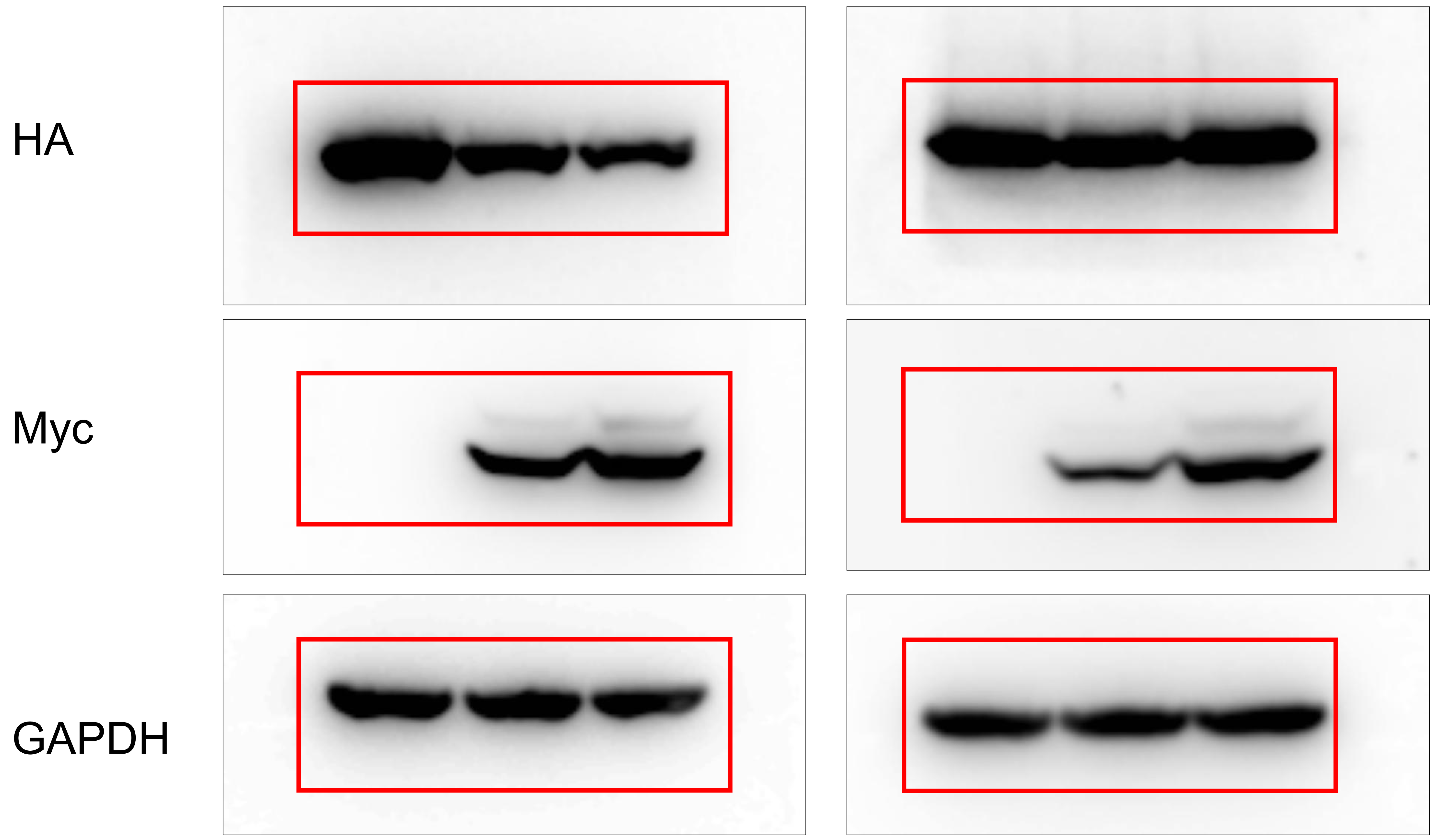

Figure 6A

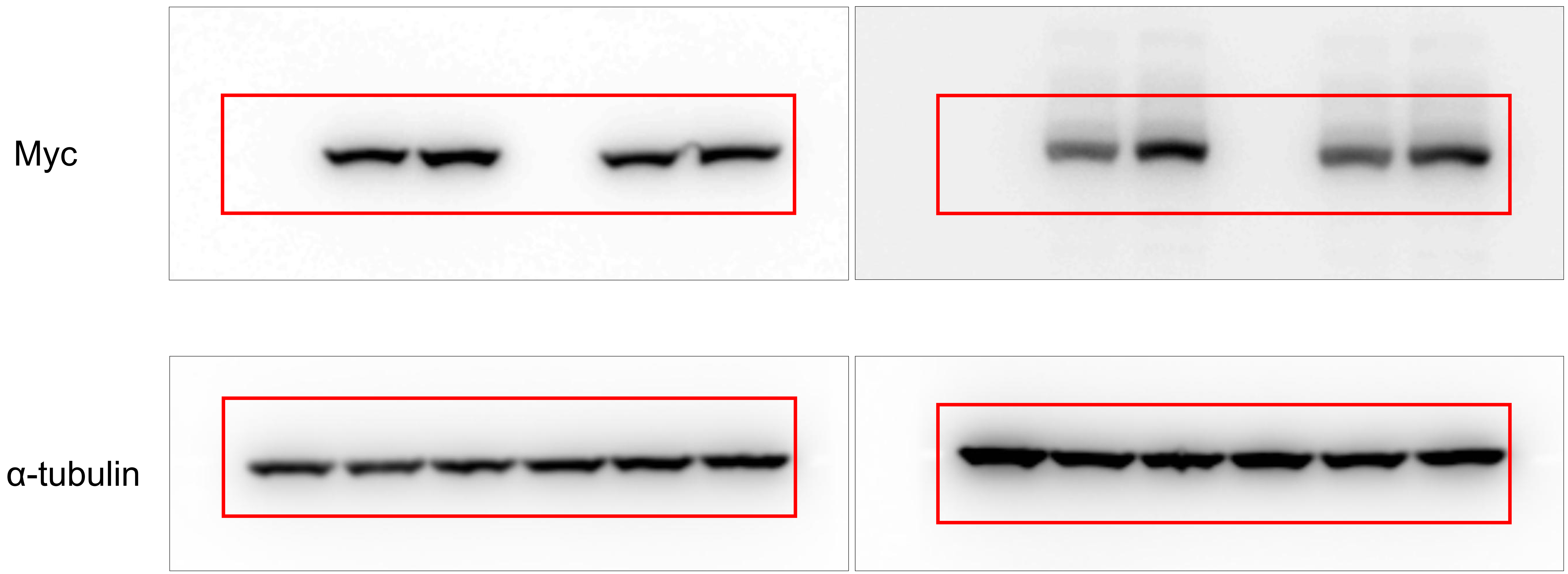

Figure 6B

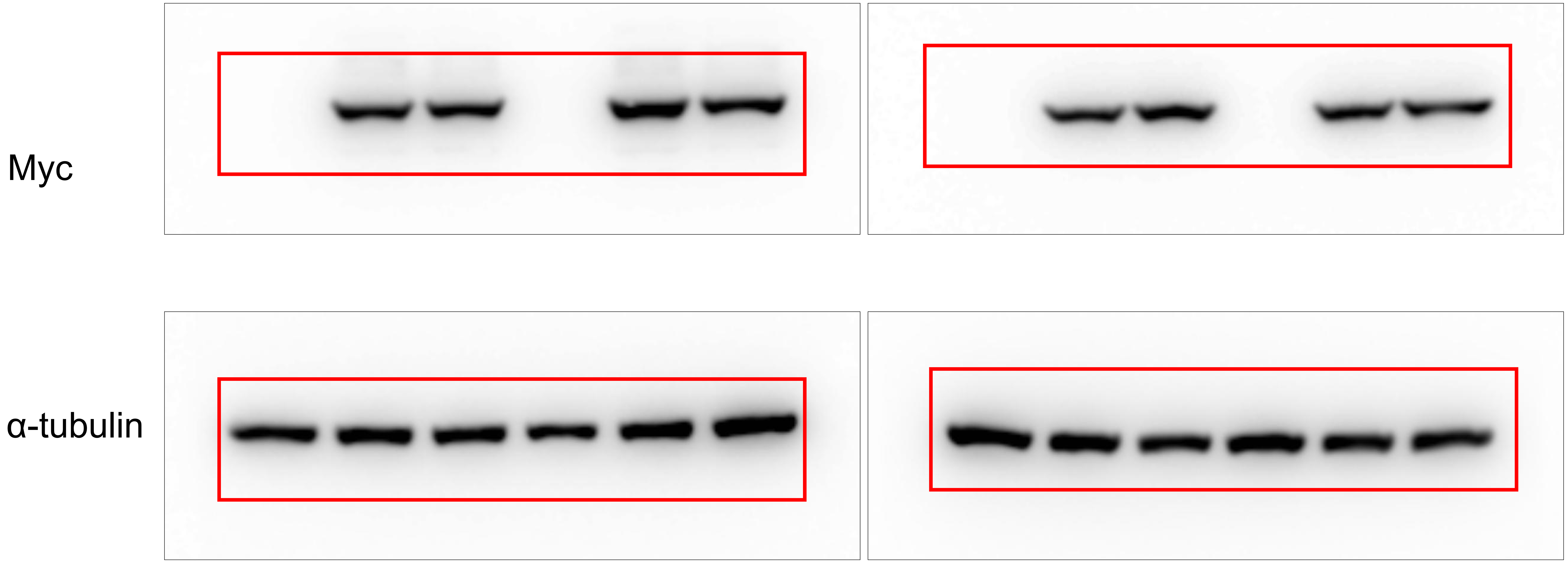

Figure 6C

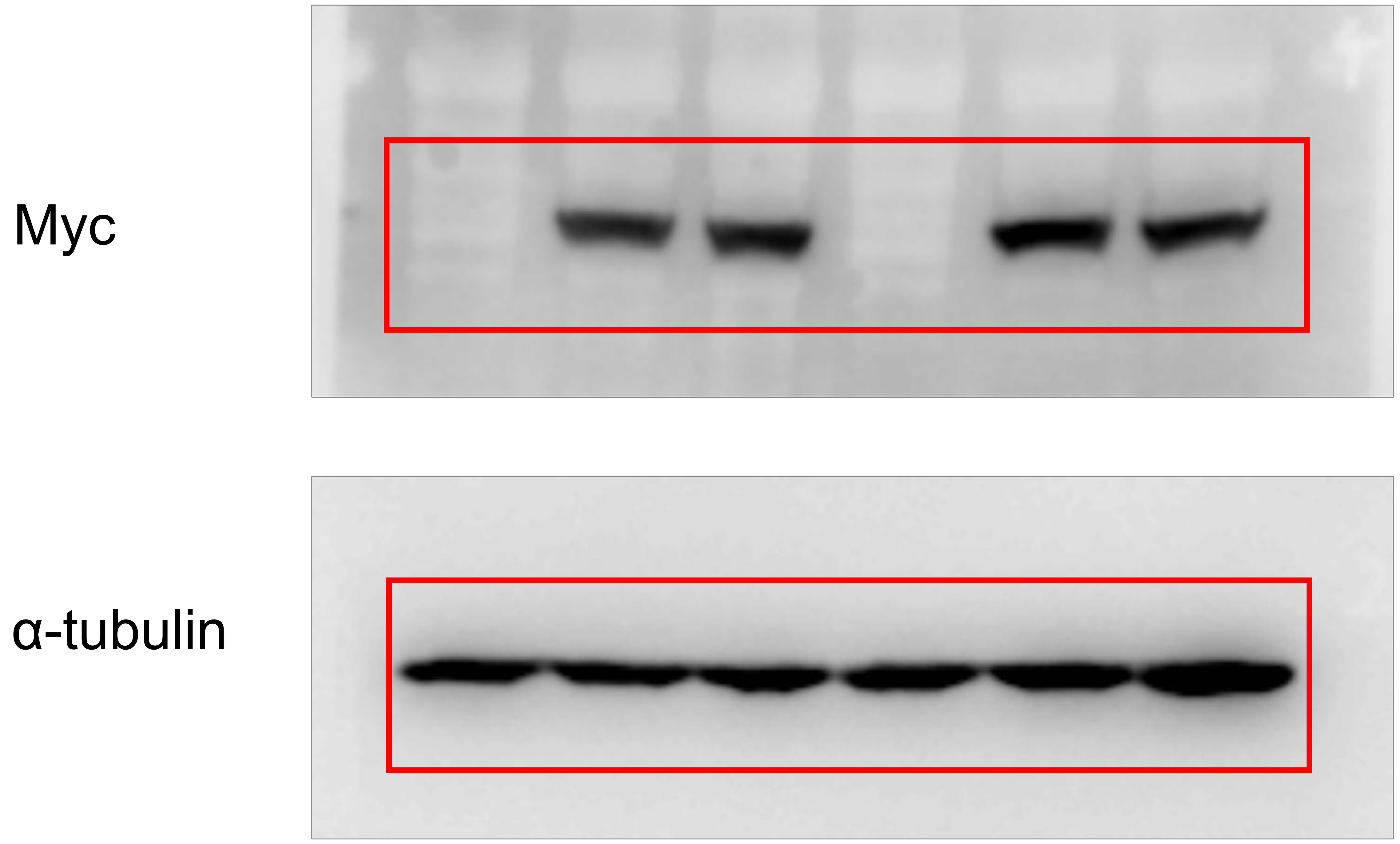

Figure 6D

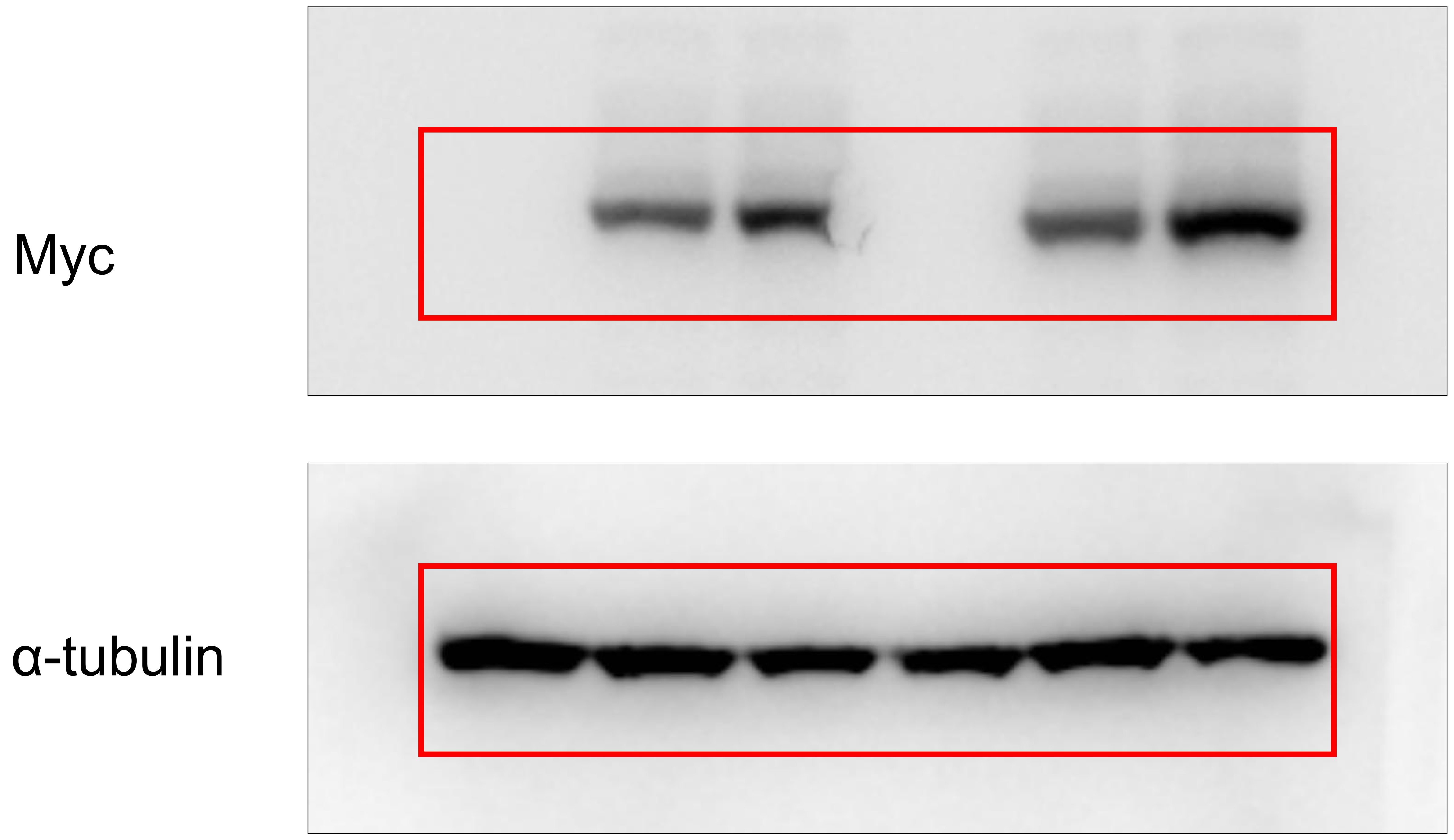

Figure 6G

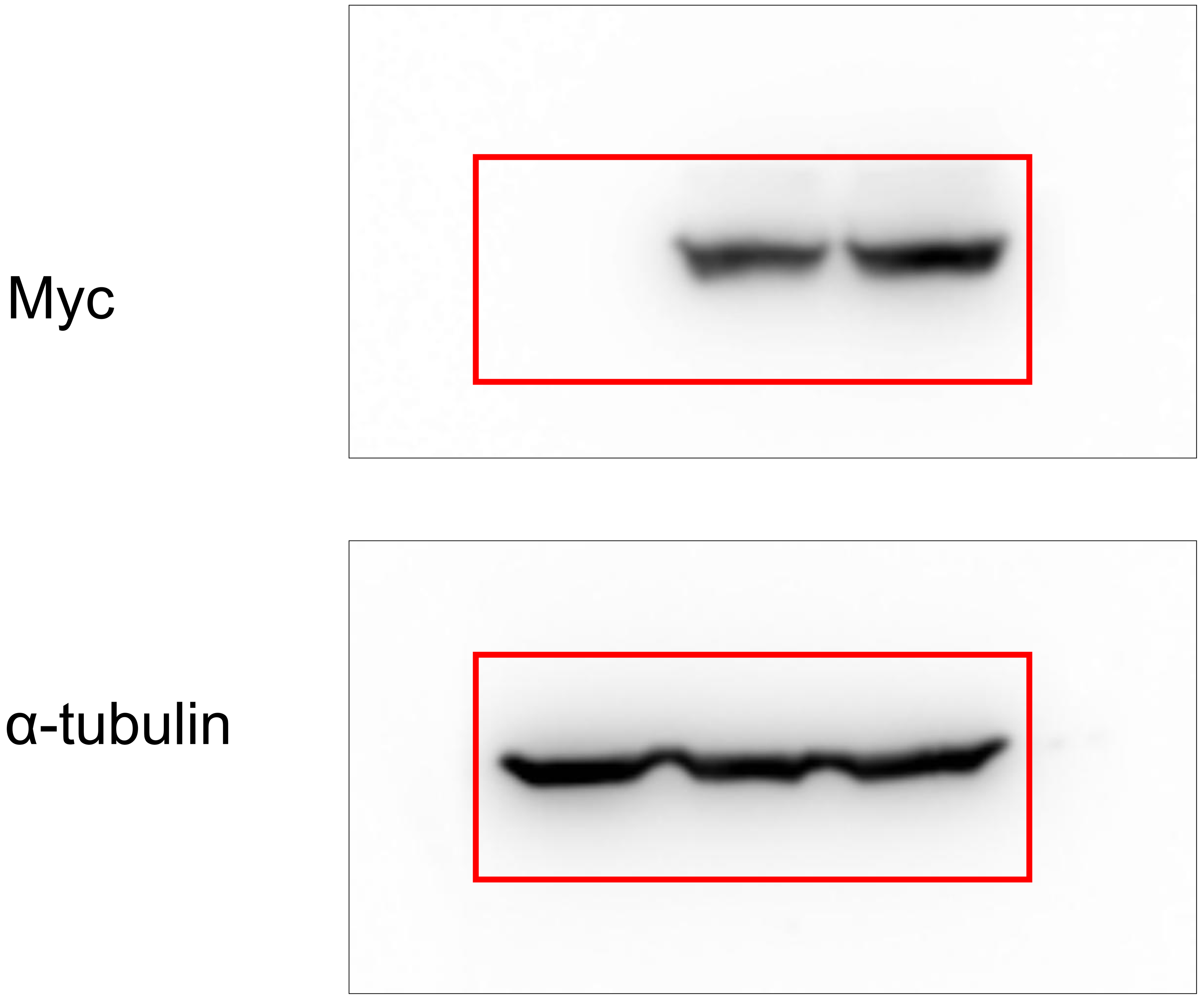

Figure 7A

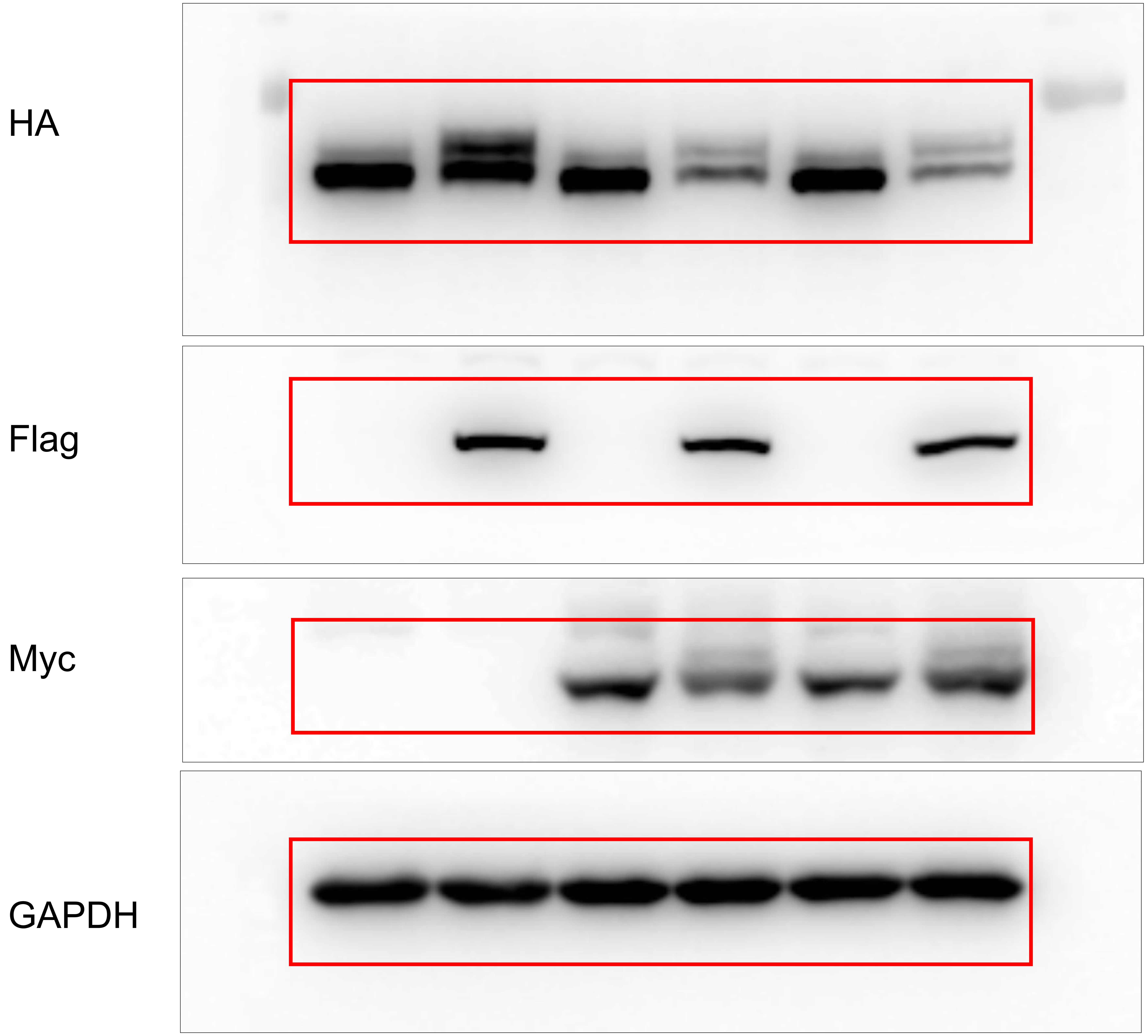

Figure 7B

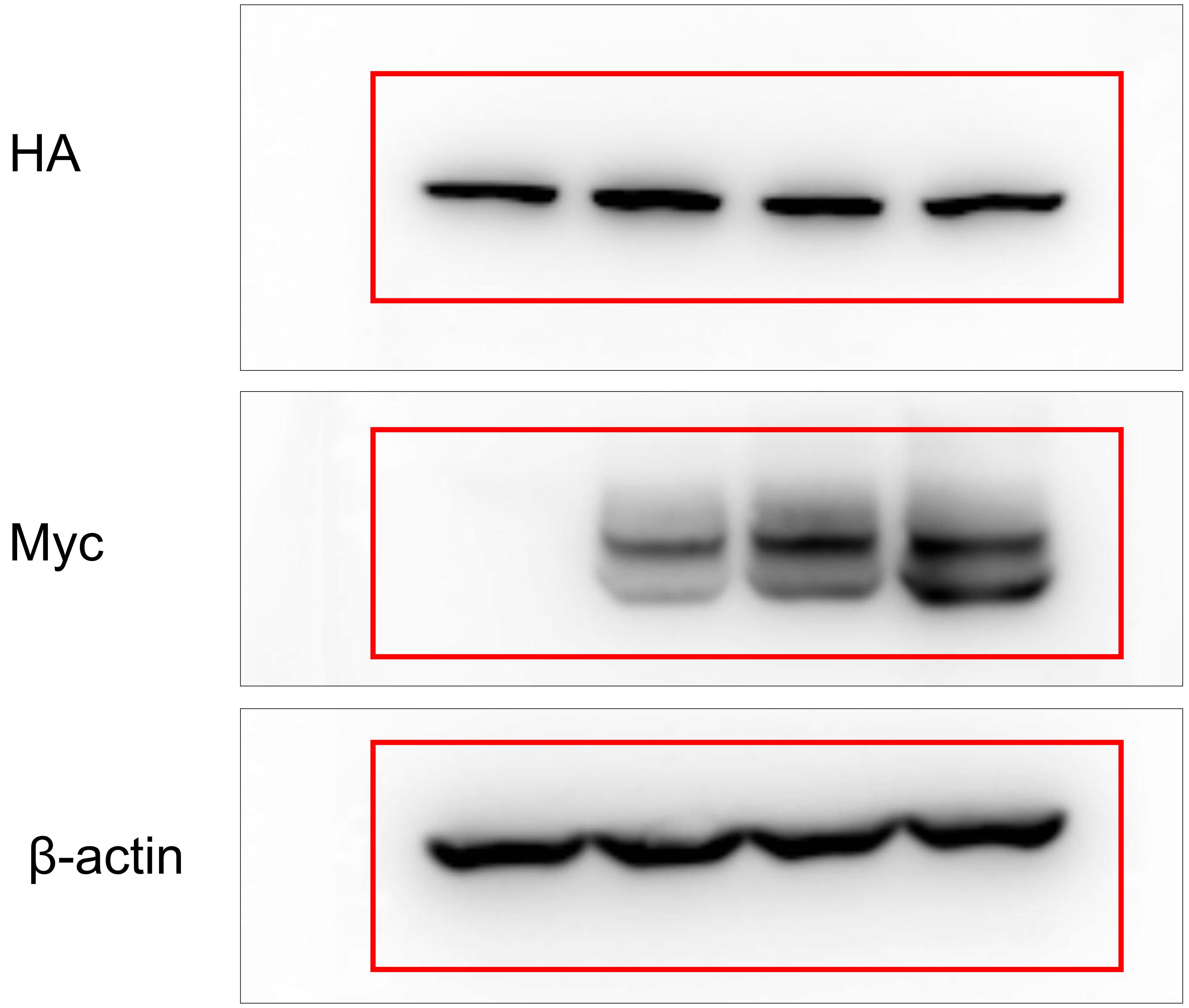

Figure 7C

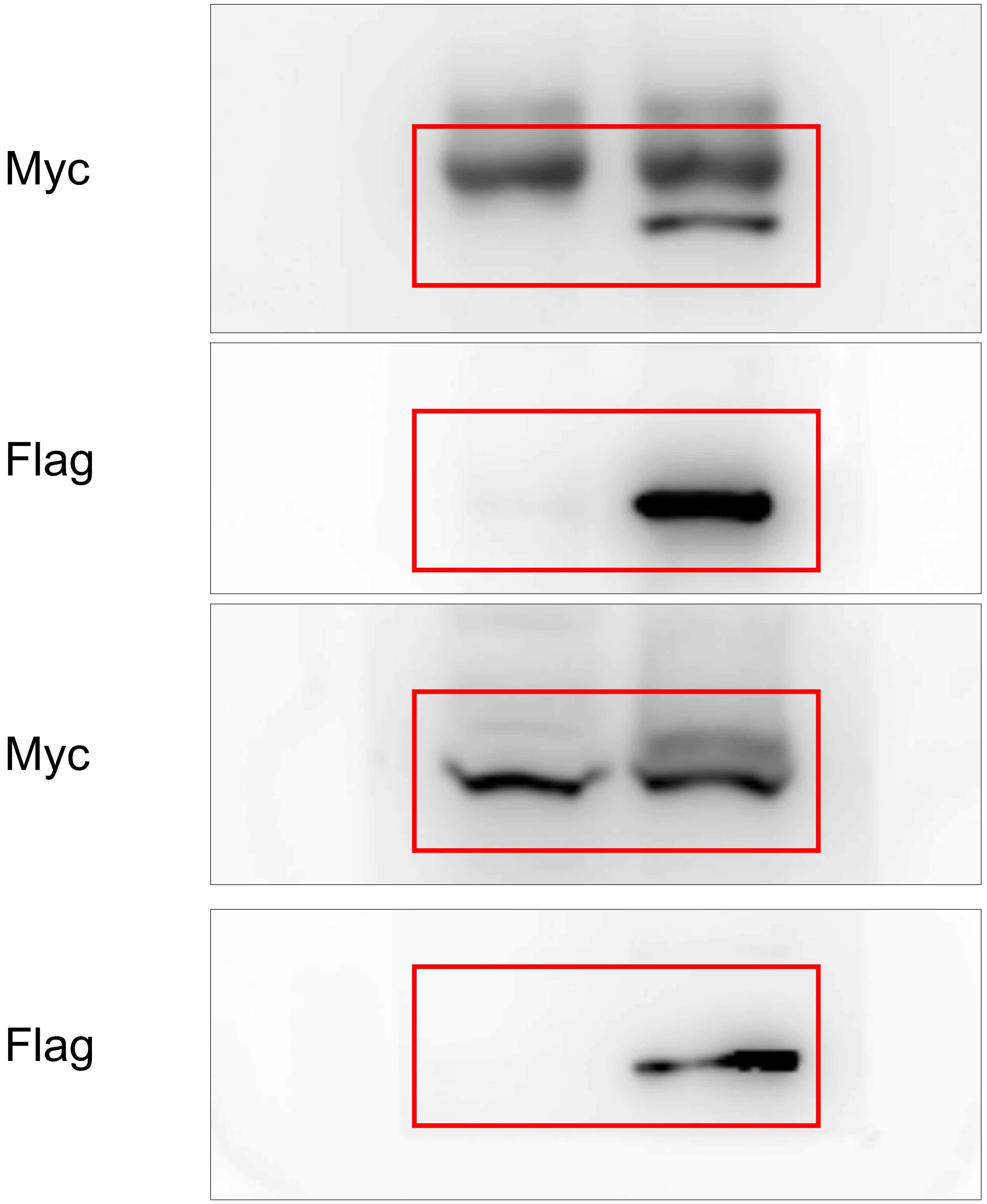

Figure 7D

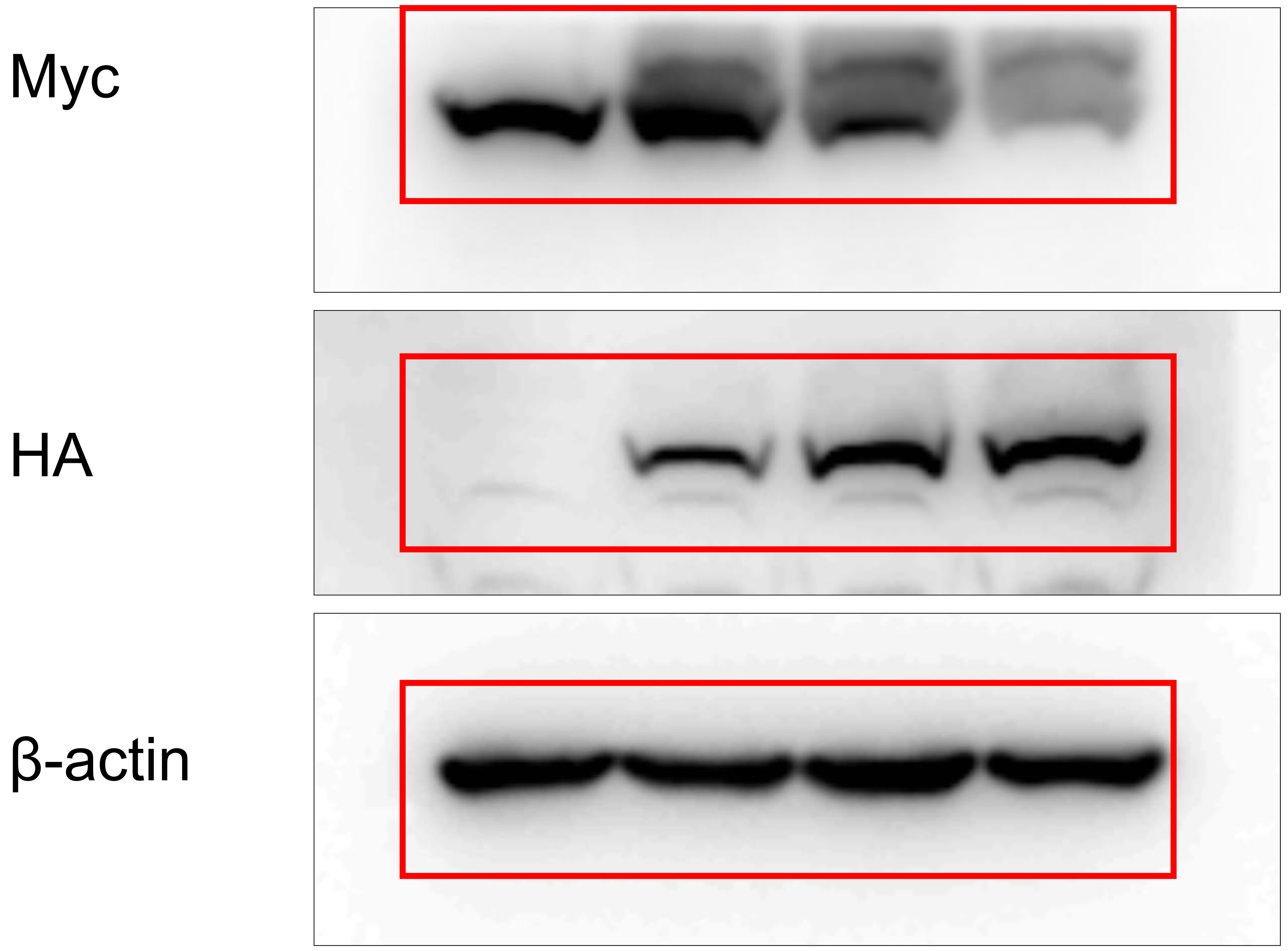

Figure 7E

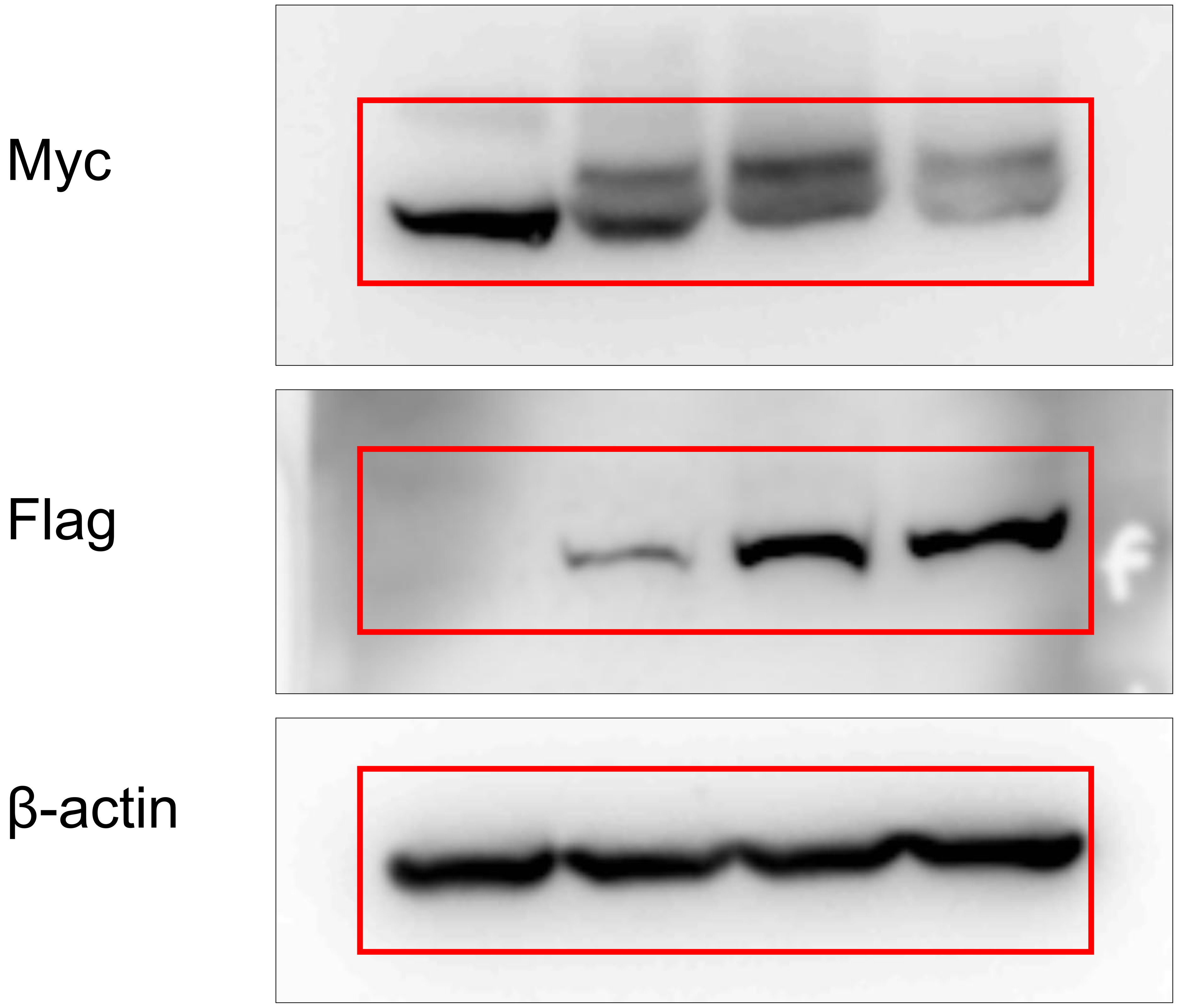

Figure 7F

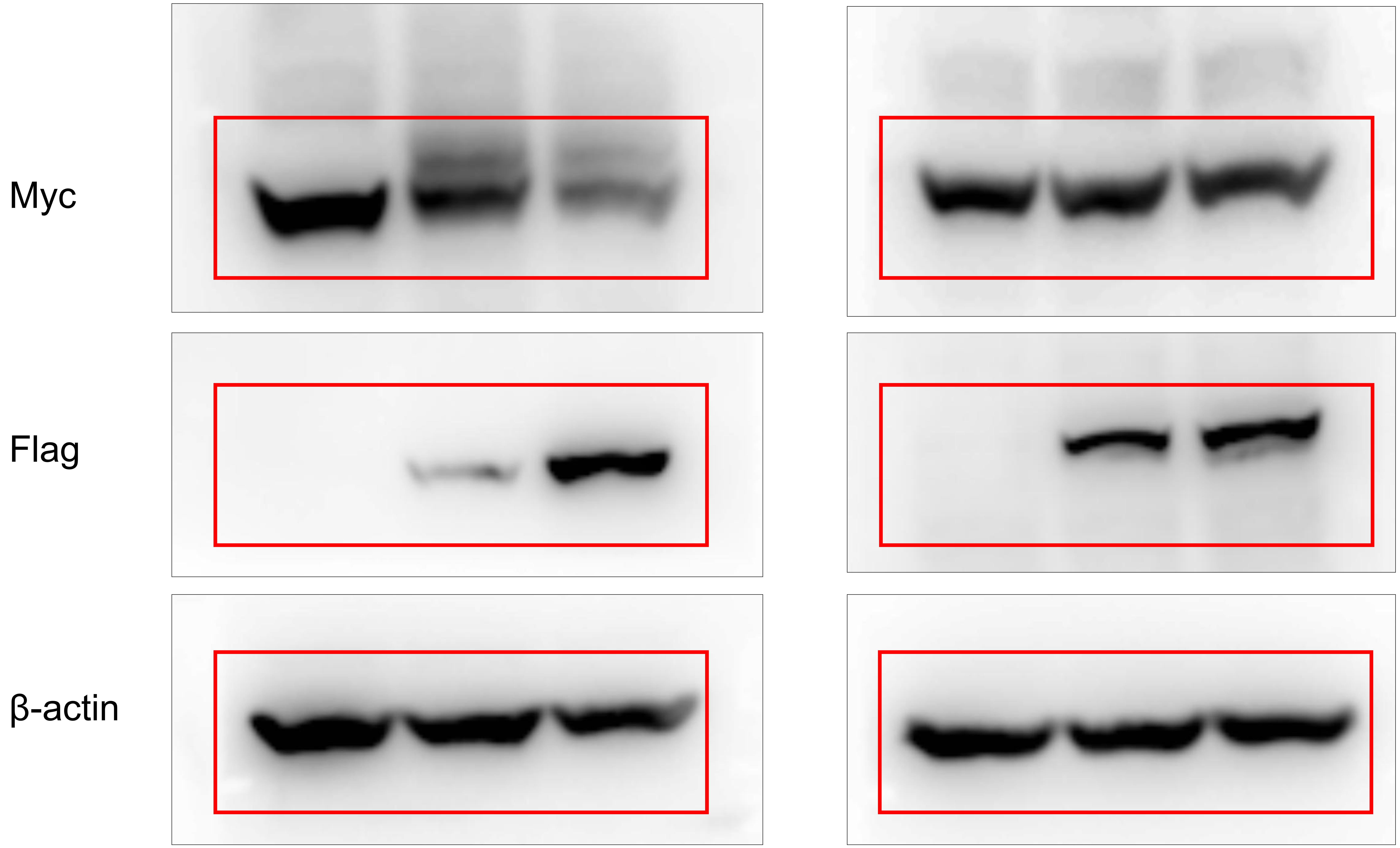

Figure 7G

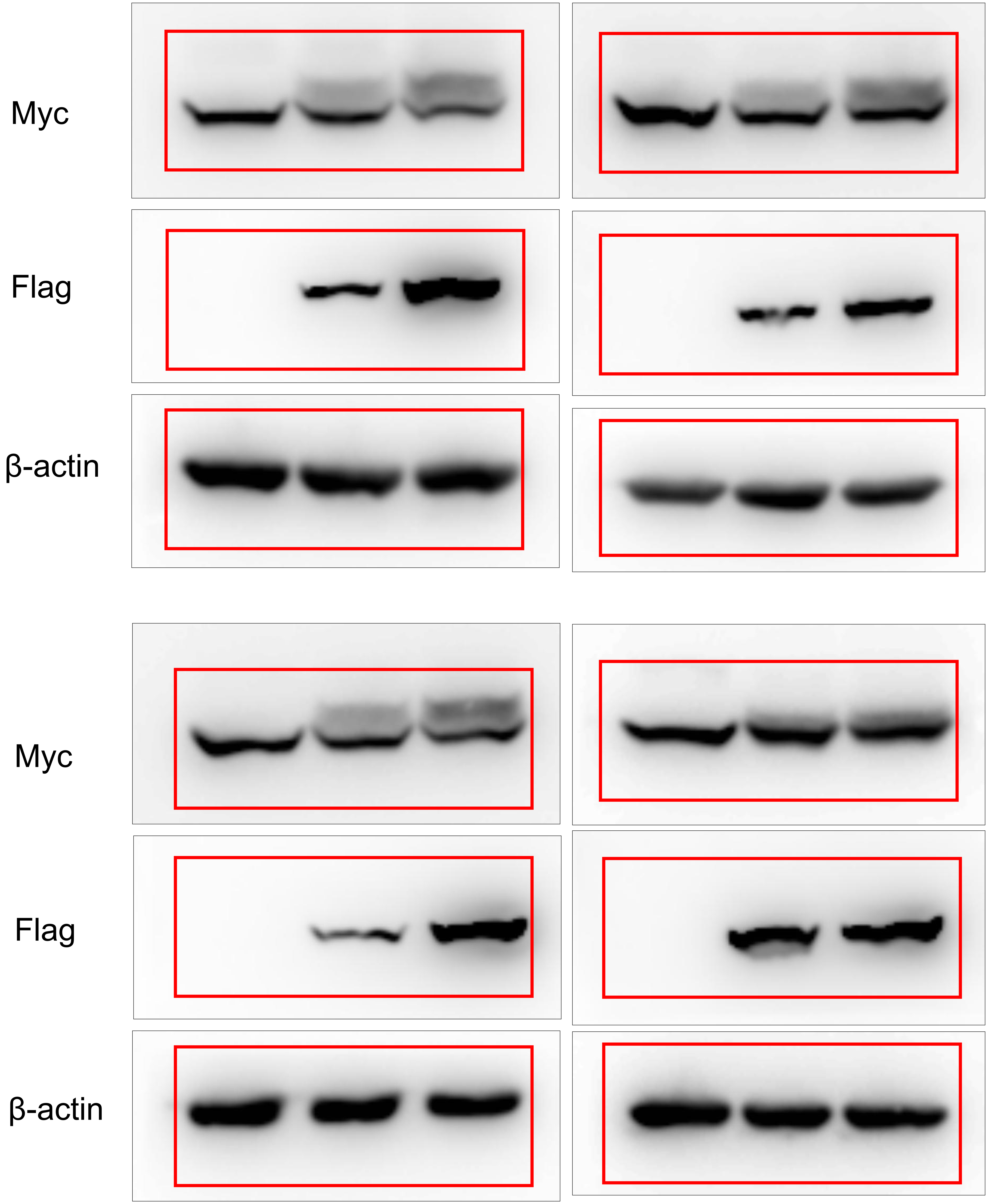

Figure 7H

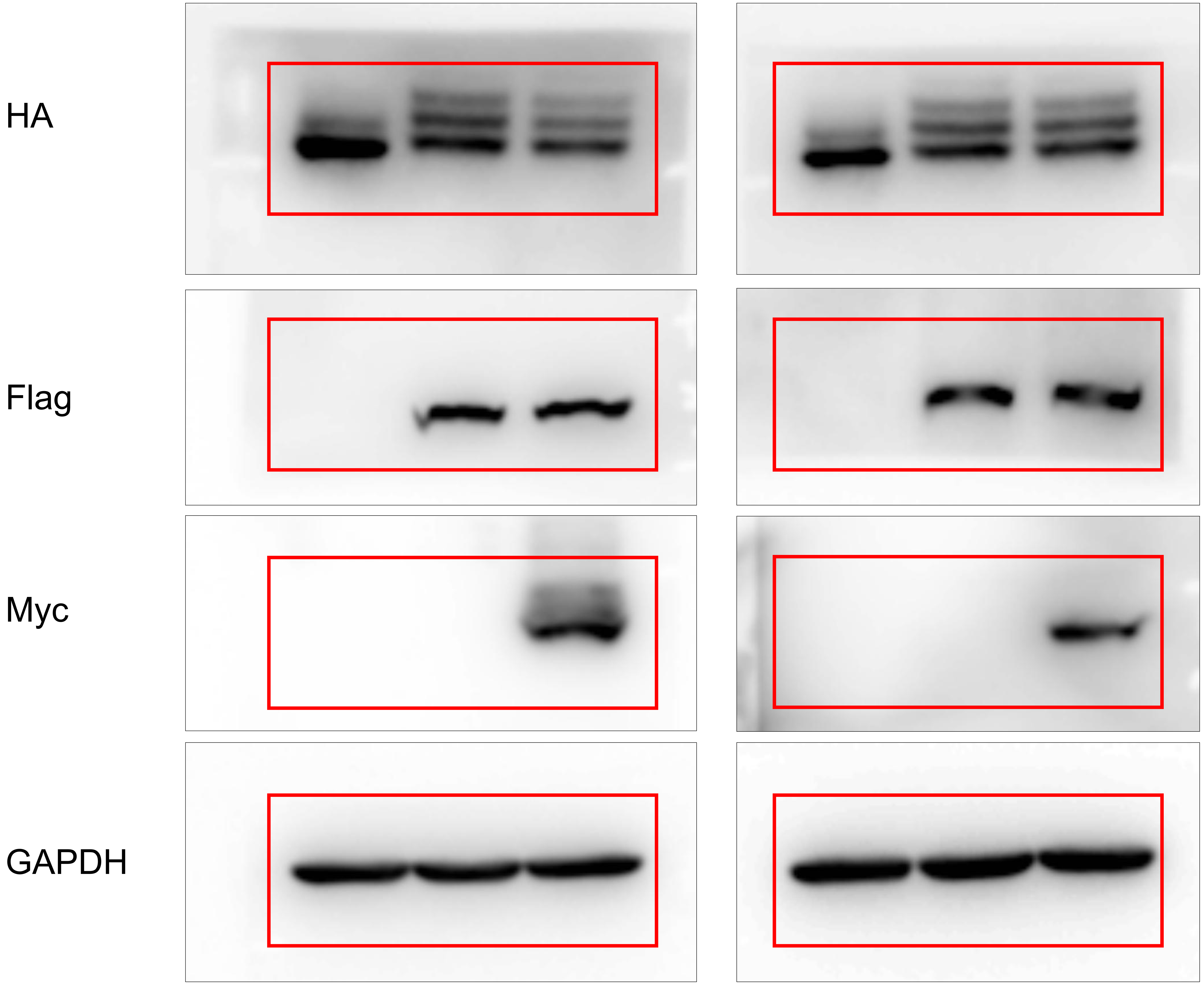

Figure 7K

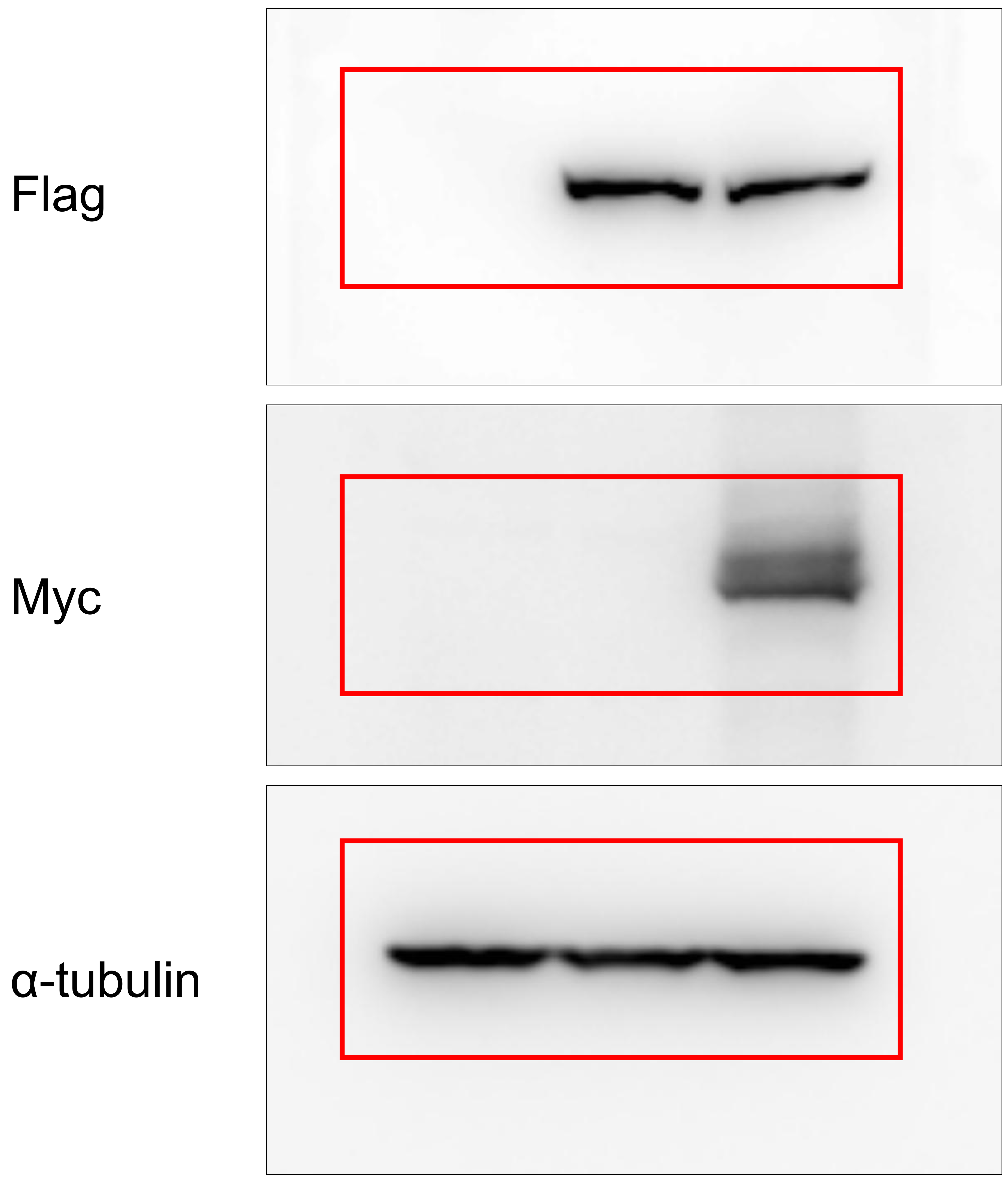

Figure 7L

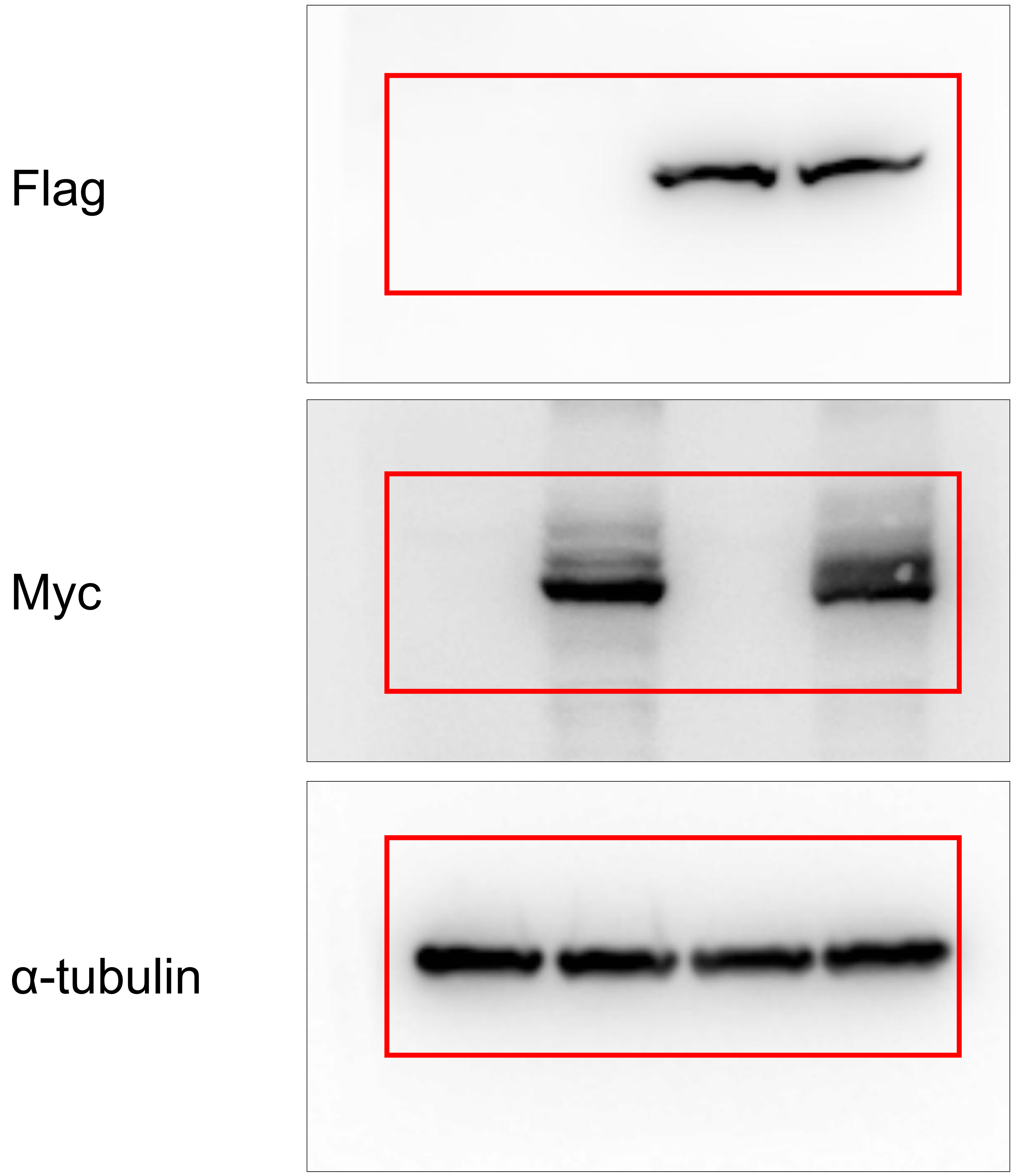

Figure S3A

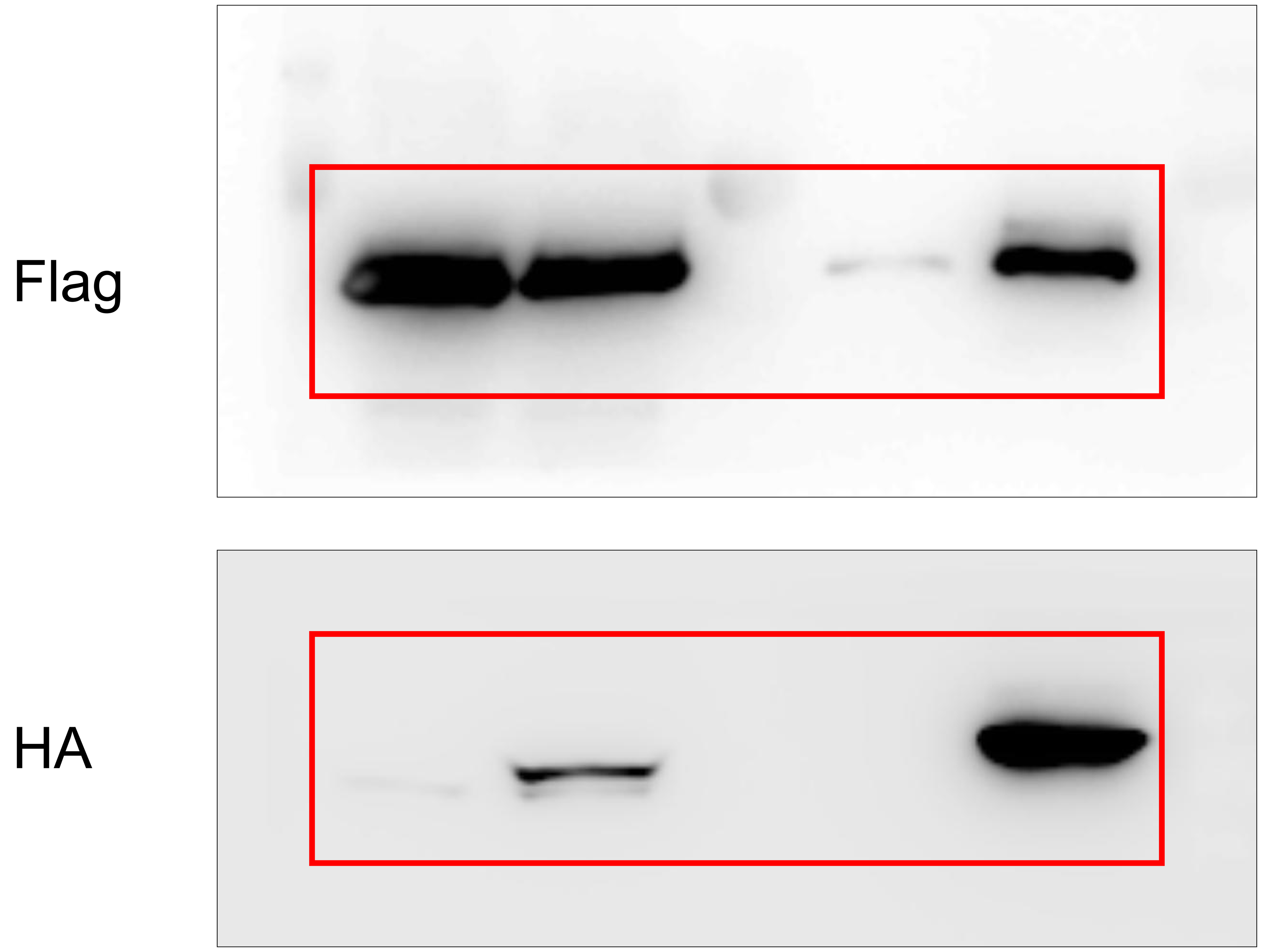

Figure S3B

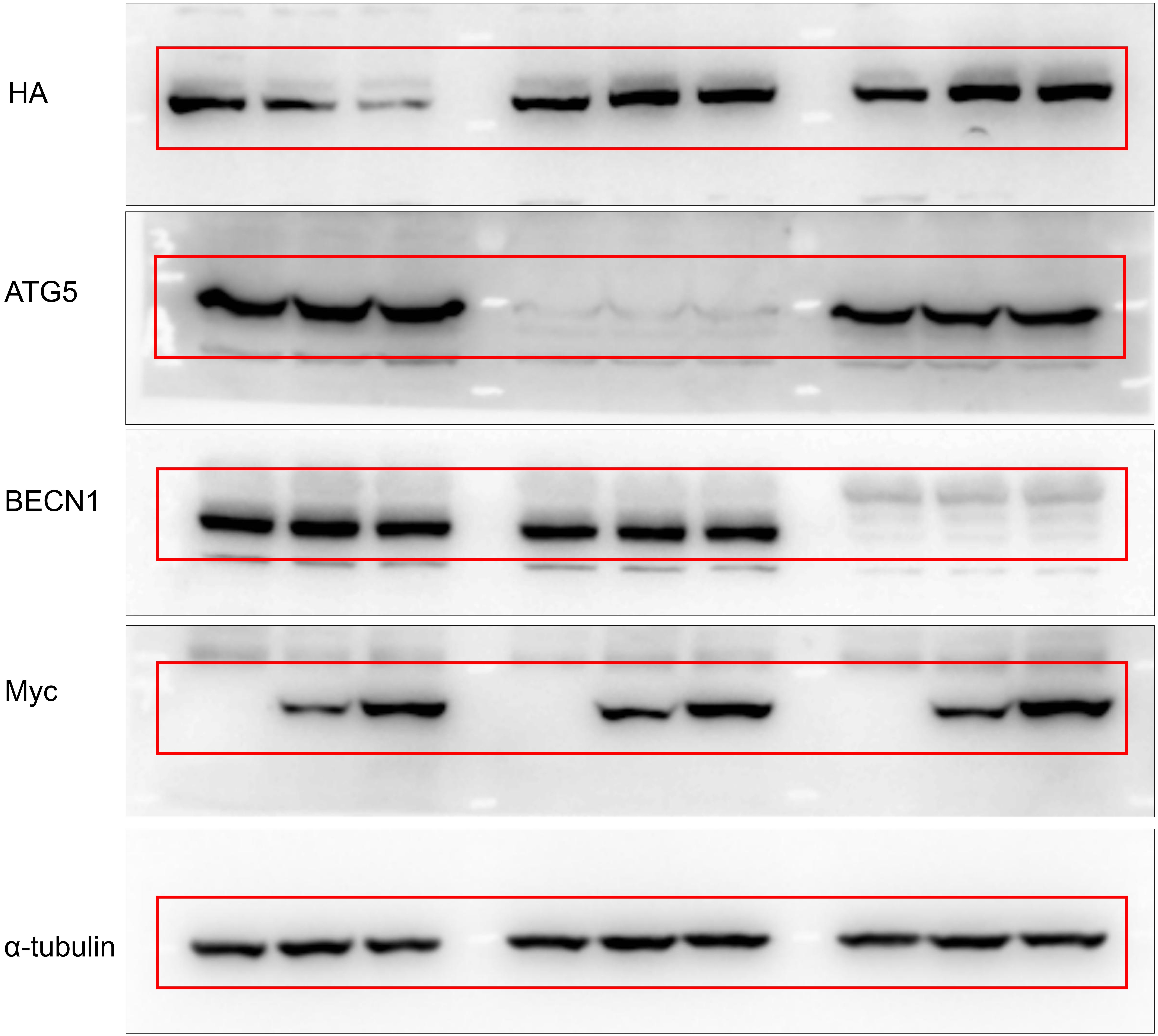

Figure S3C

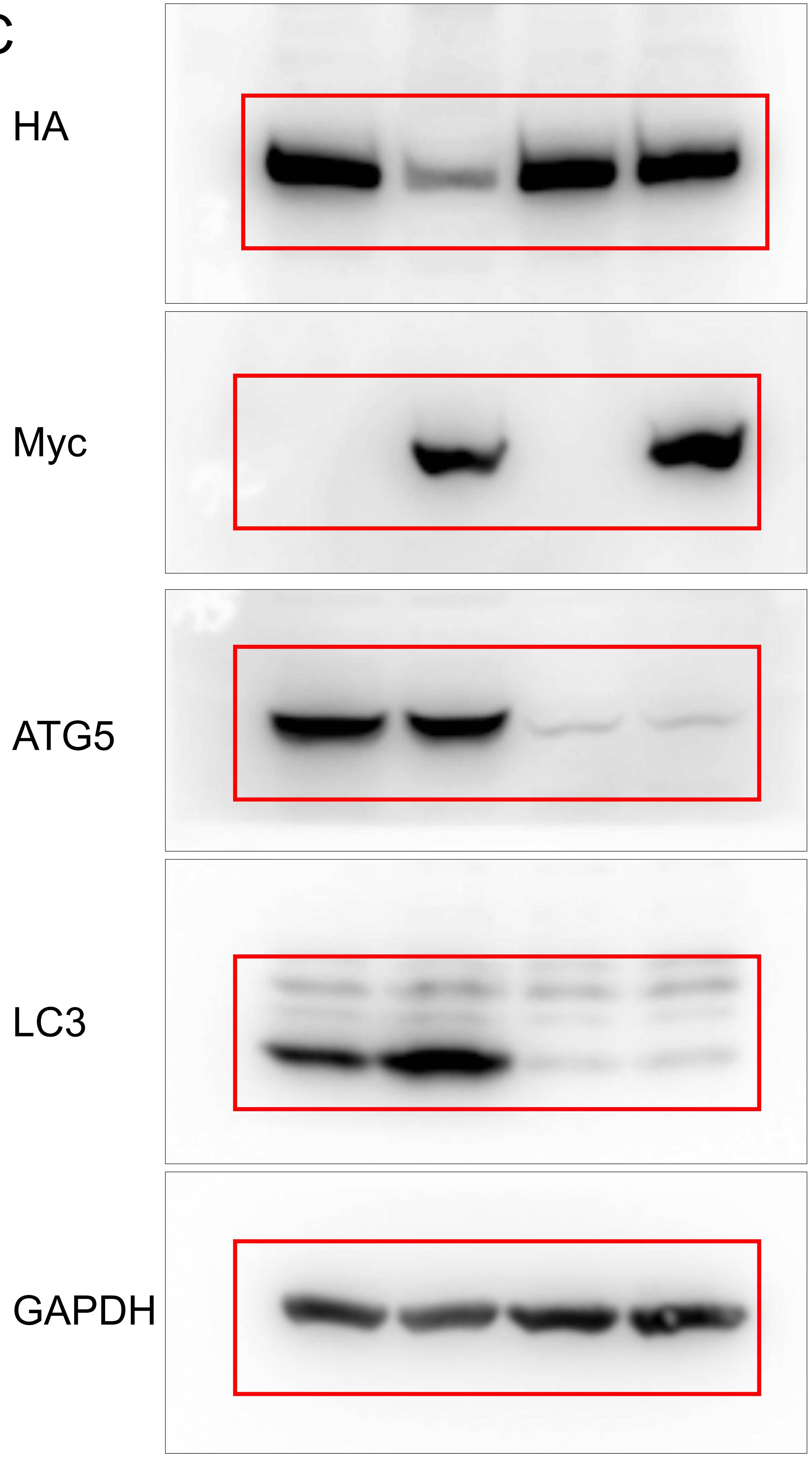

Figure S5A

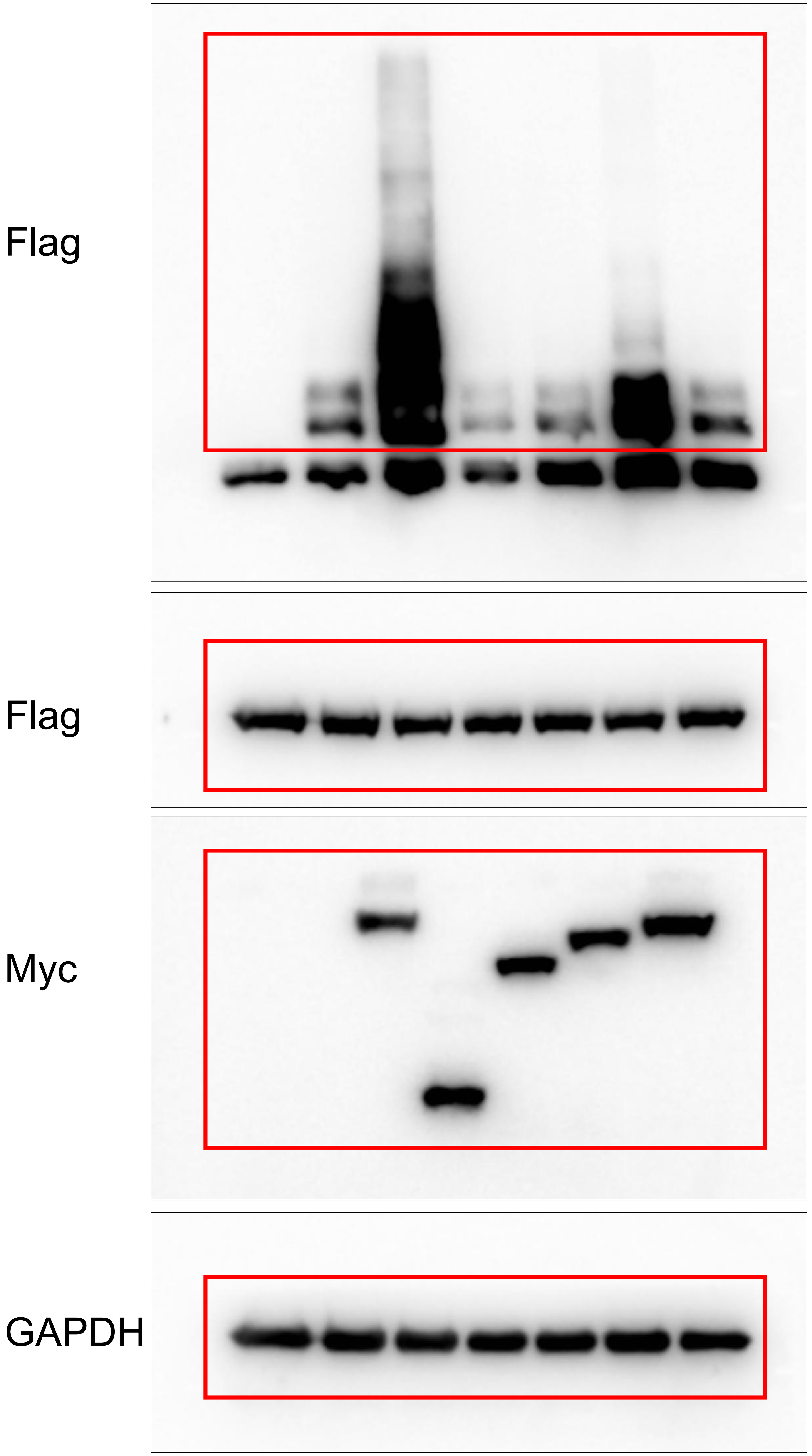

Figure S5B

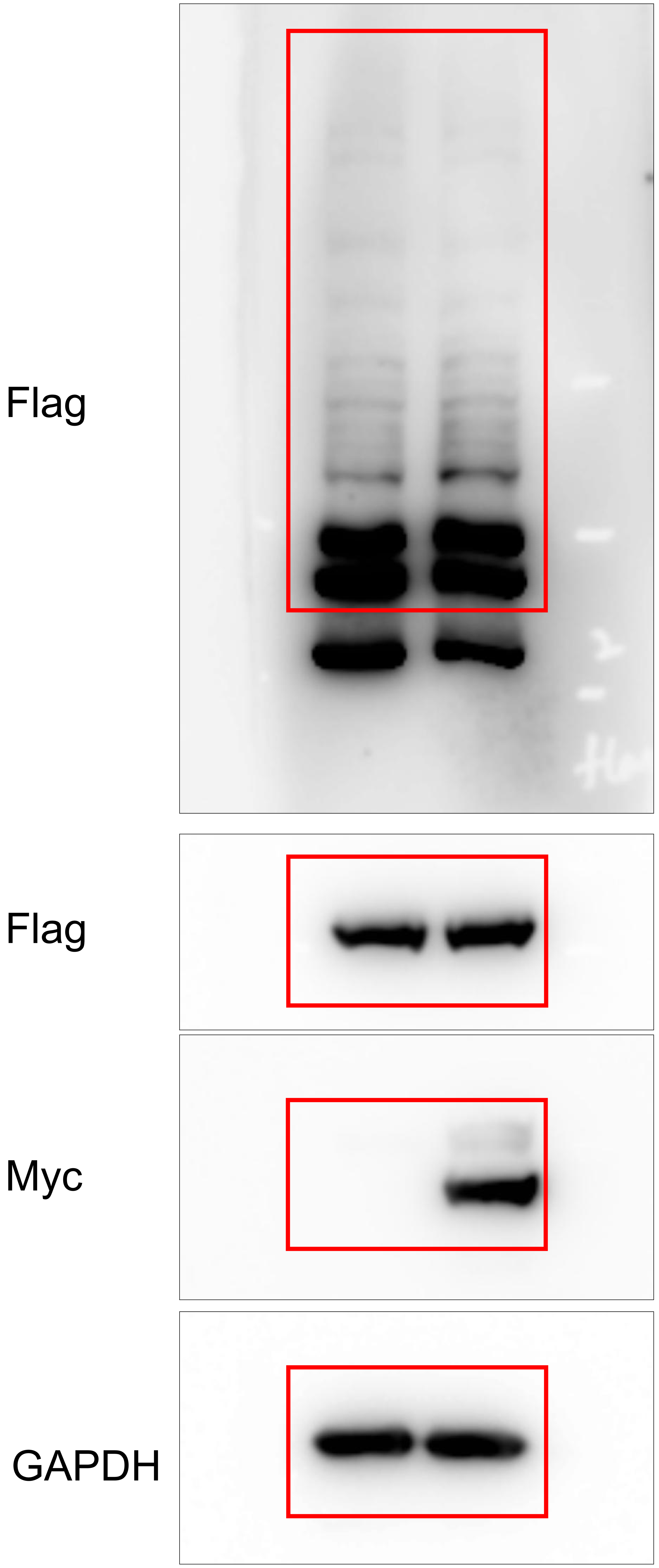

Figure S6B

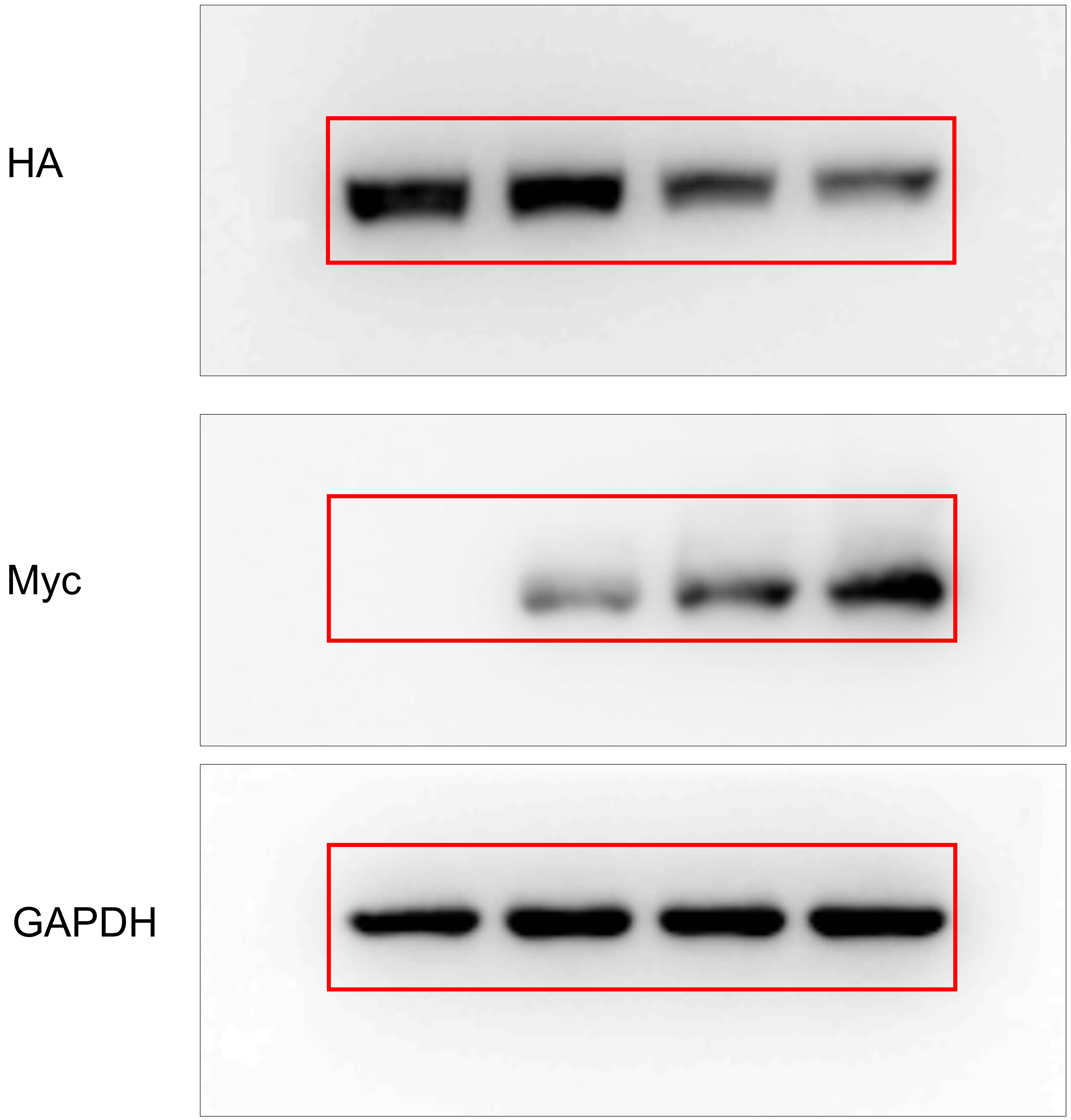

Figure S7A

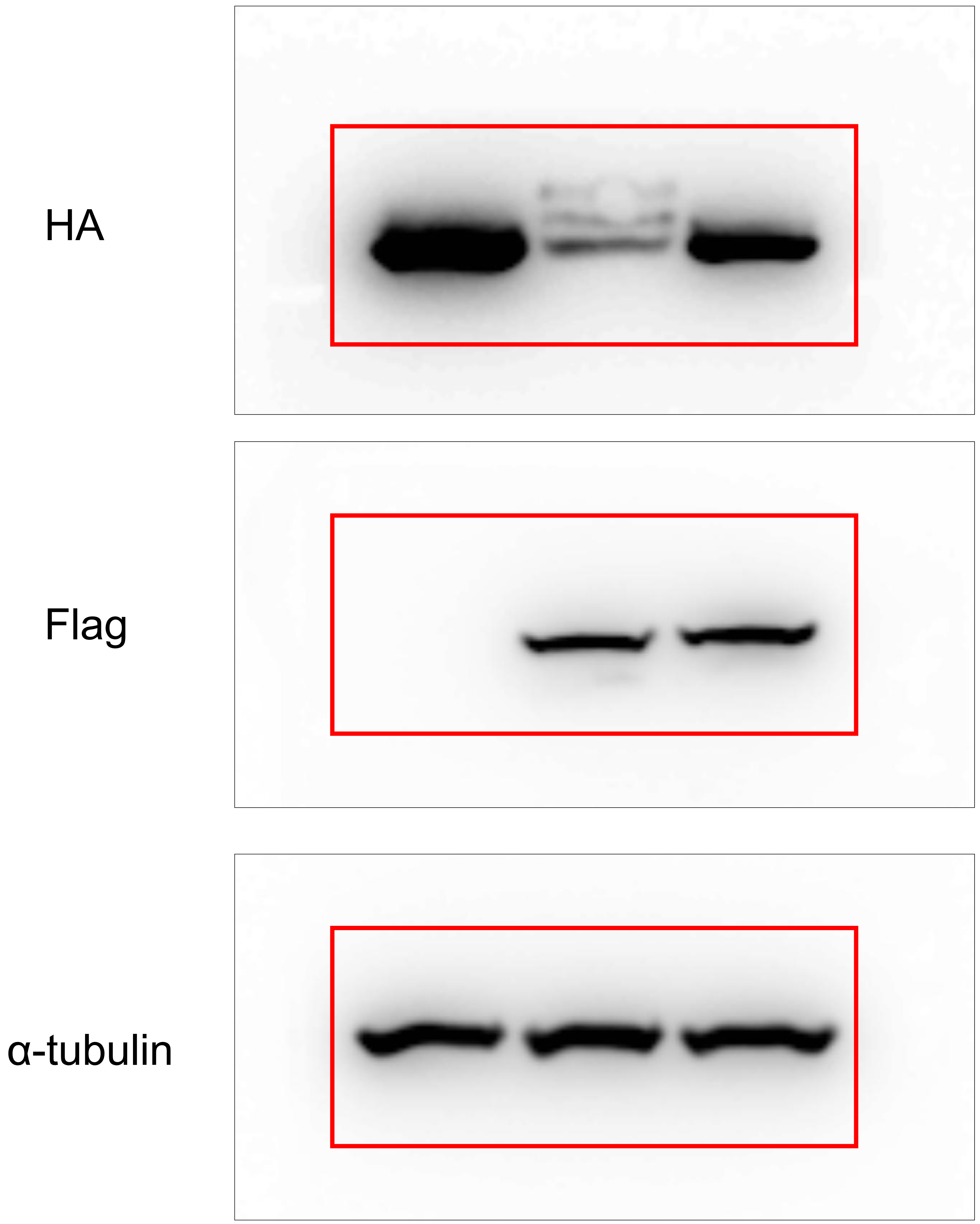

Figure S7B

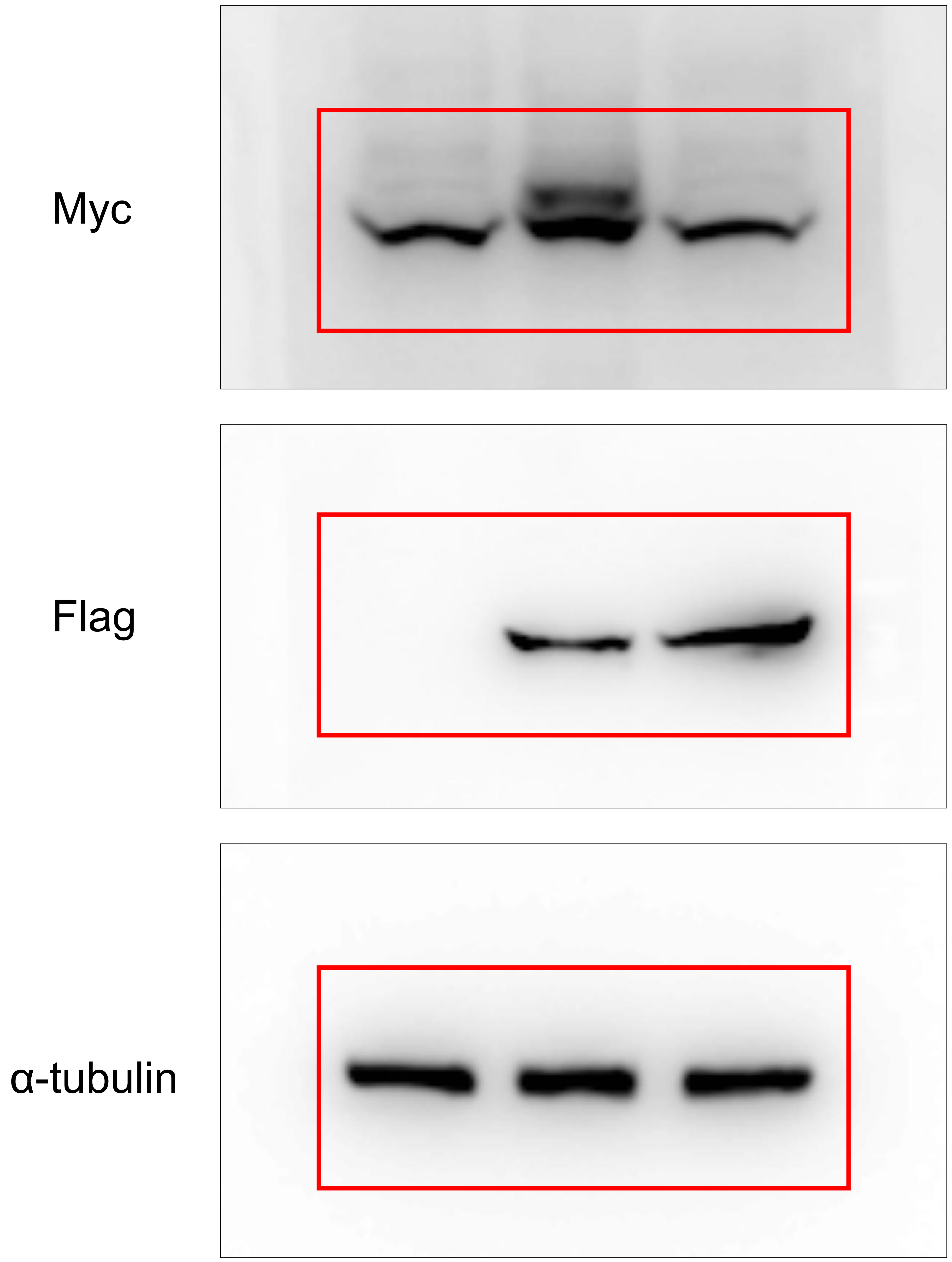

Figure S9B

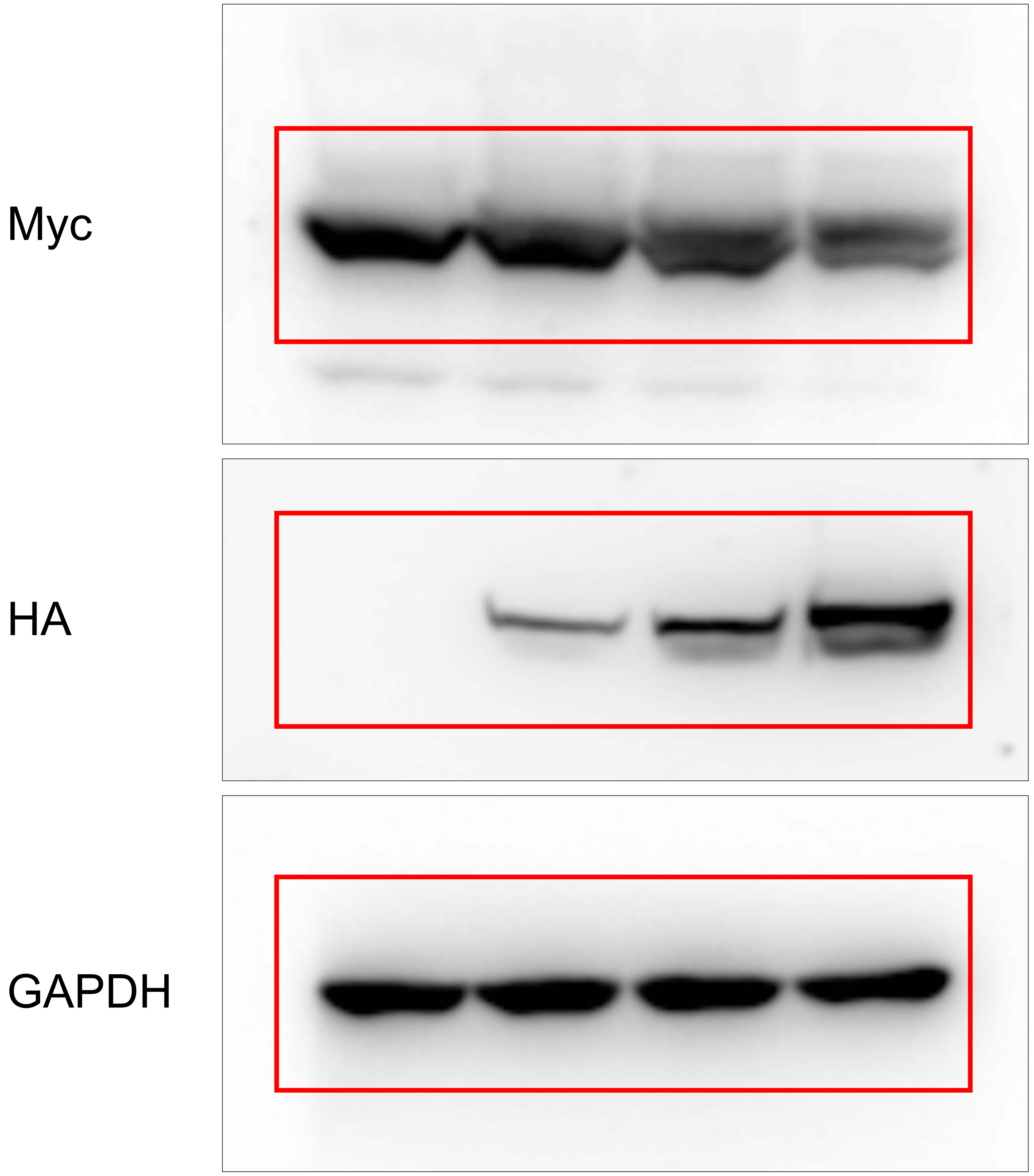

Figure S10A

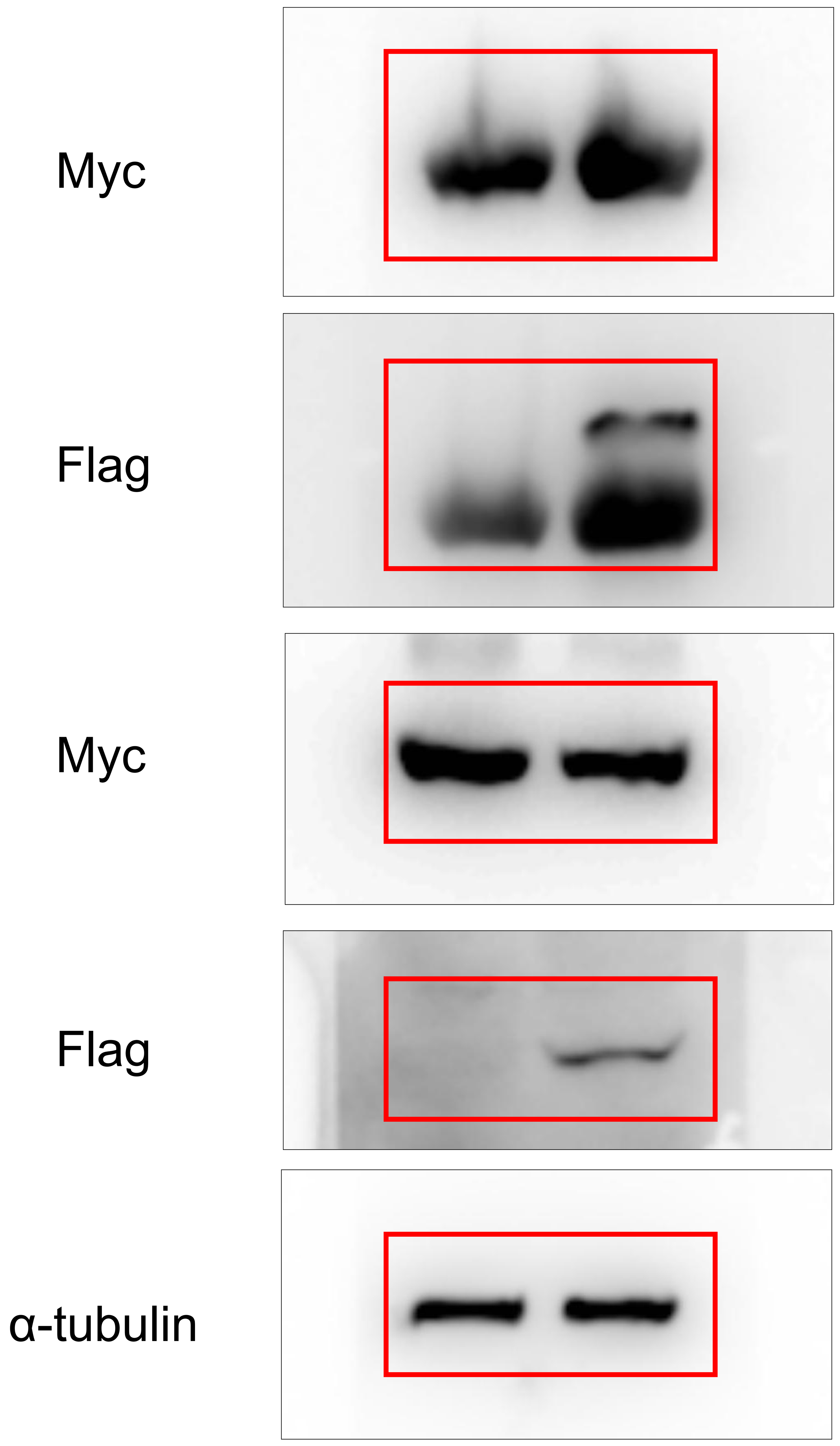

Figure S10B

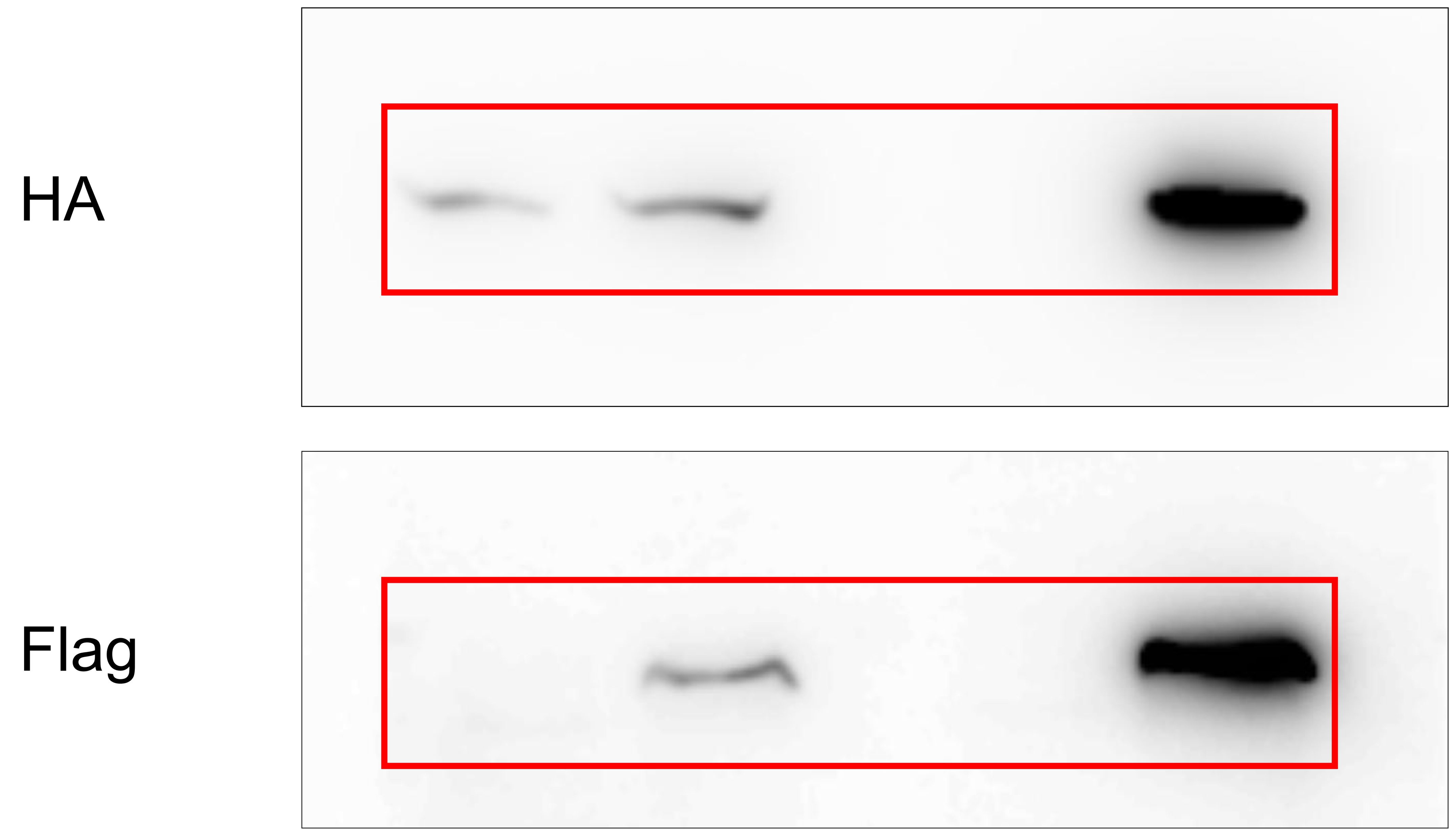

Figure S10C

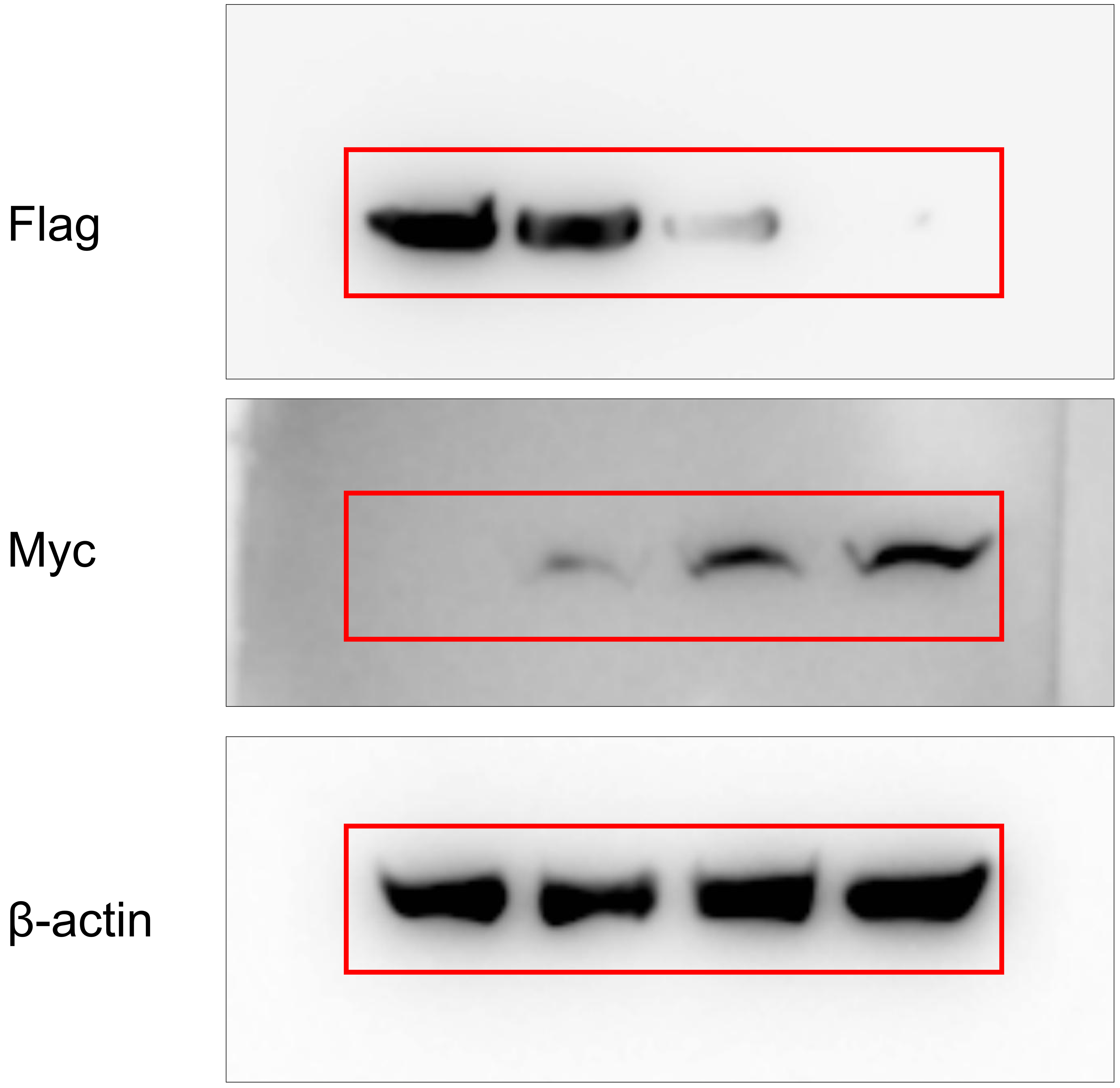

Figure S10D

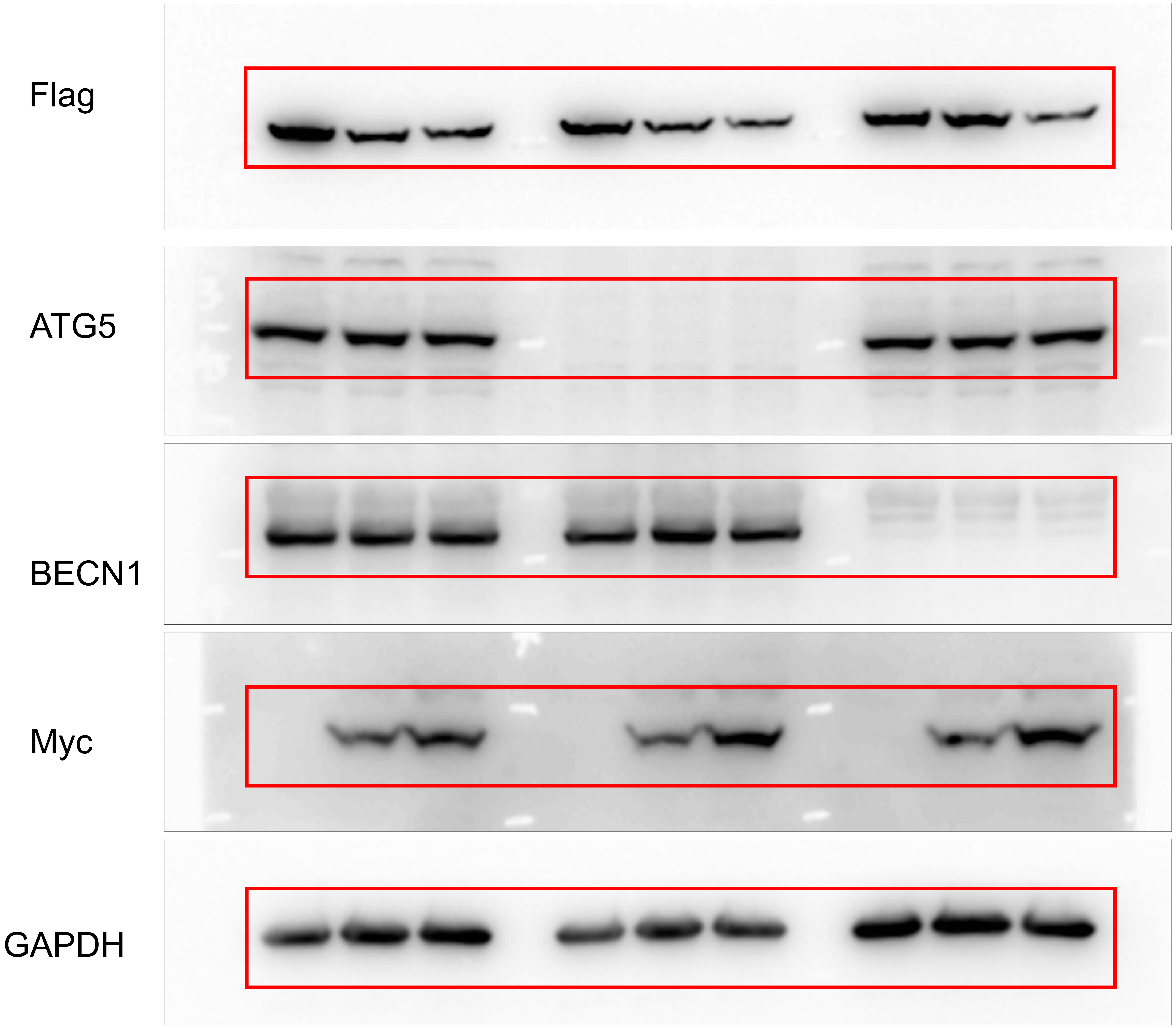

Figure S10E

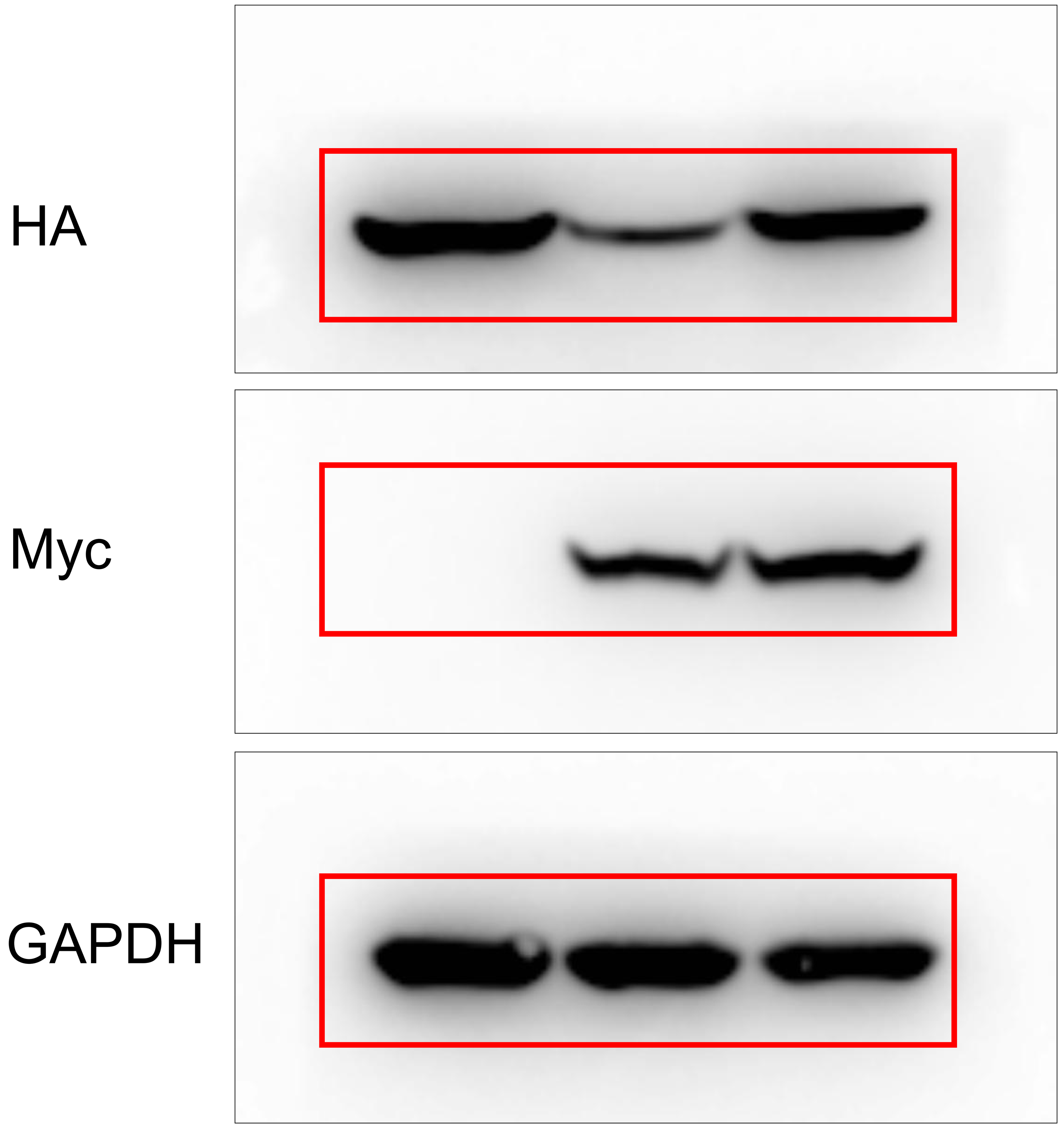

Supplement: S2 Data — (PDF) [file ppat.1012227.s013.pdf]
