# Supplementary material for: Zebrafish mylipb attenuates antiviral innate immunity through two synergistic mechanisms targeting transcription factor irf3
Source: PLoS Pathog. 2024 May 13;20(5):e1012227. doi: 10.1371/journal.ppat.1012227 (PMC11115282; doi:10.1371/journal.ppat.1012227)

Figure 2A

*mylipb^+/+^ -*SVCV


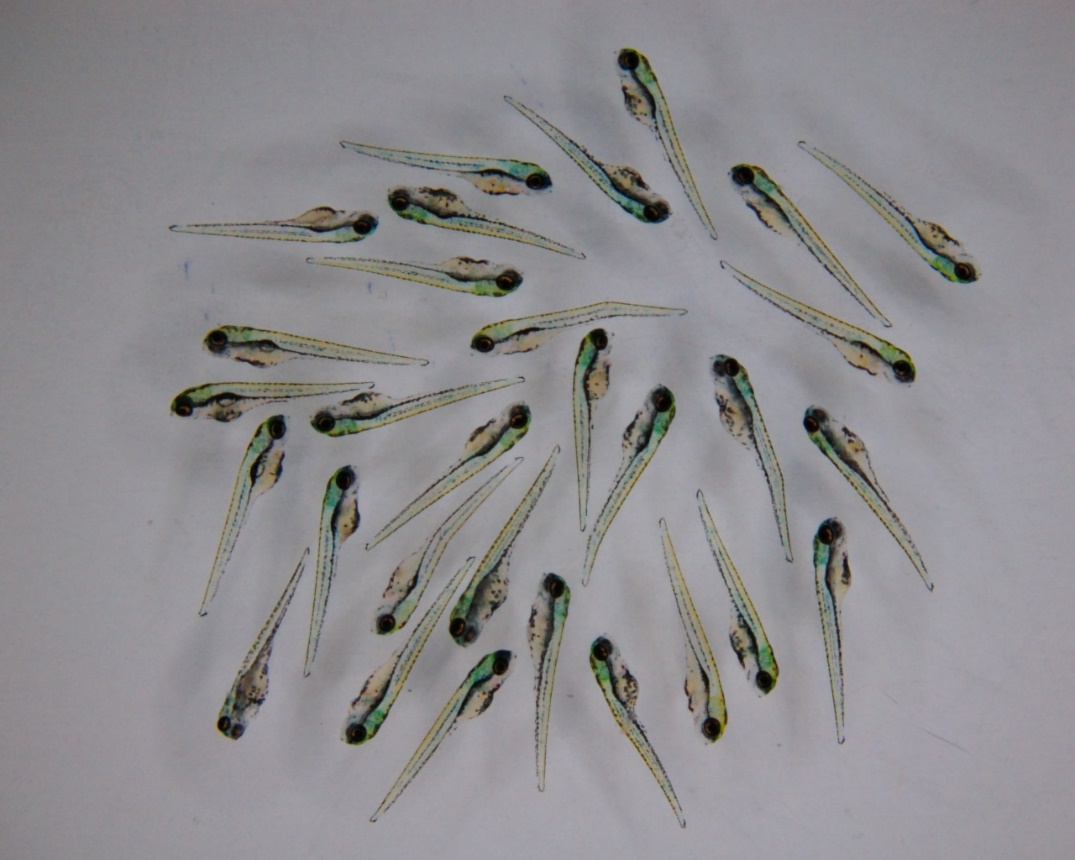


*mylipb^-/-^ -*SVCV


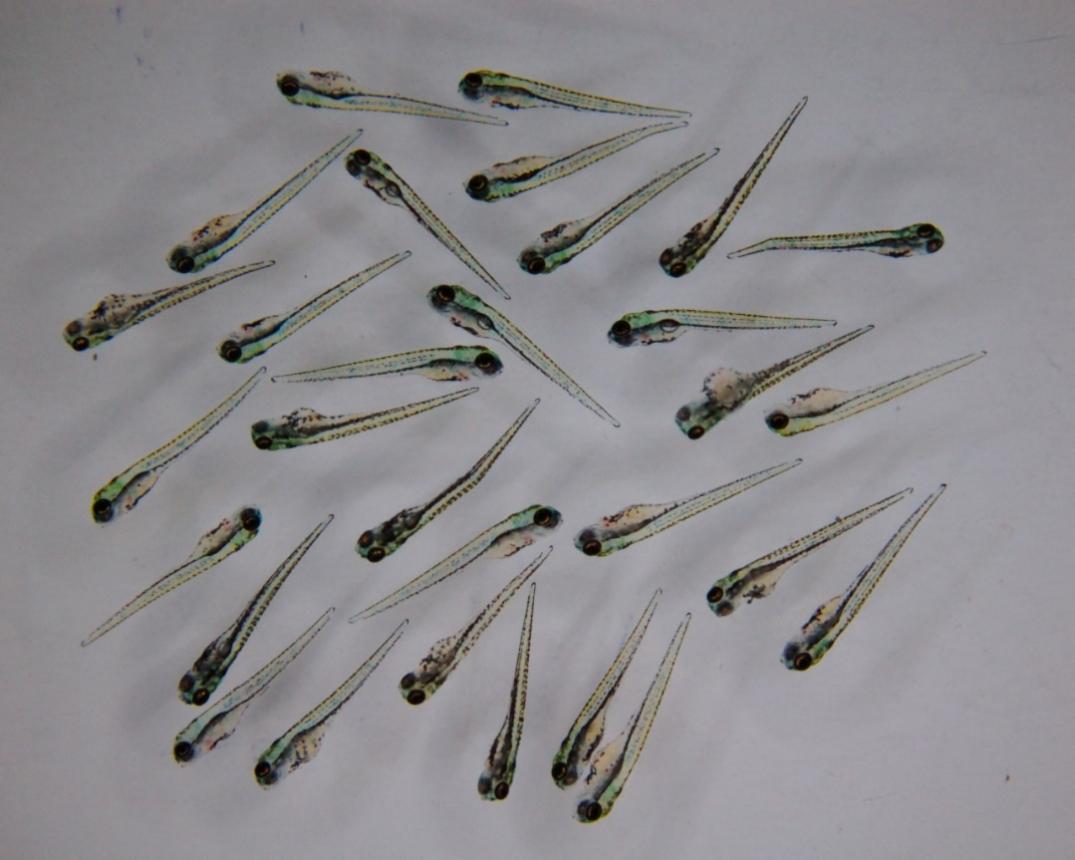


*mylipb^+/+^ +*SVCV


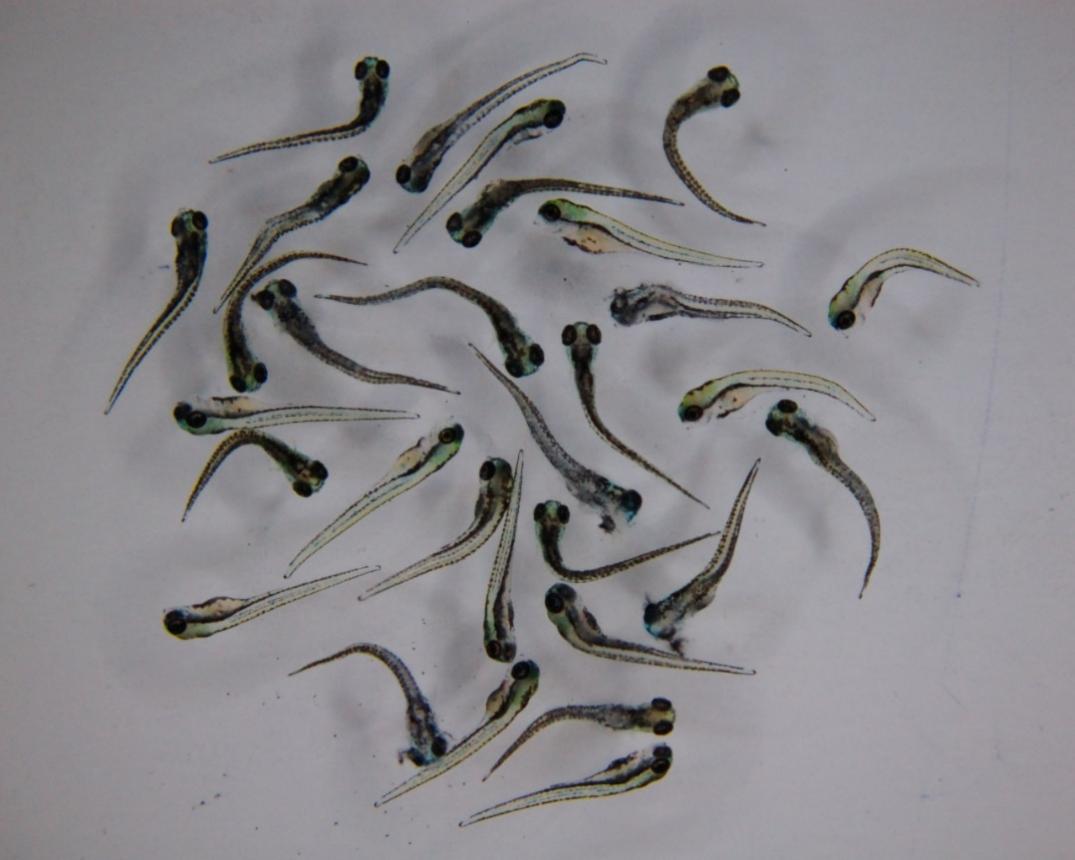


*mylipb^-/-^ +*SVCV


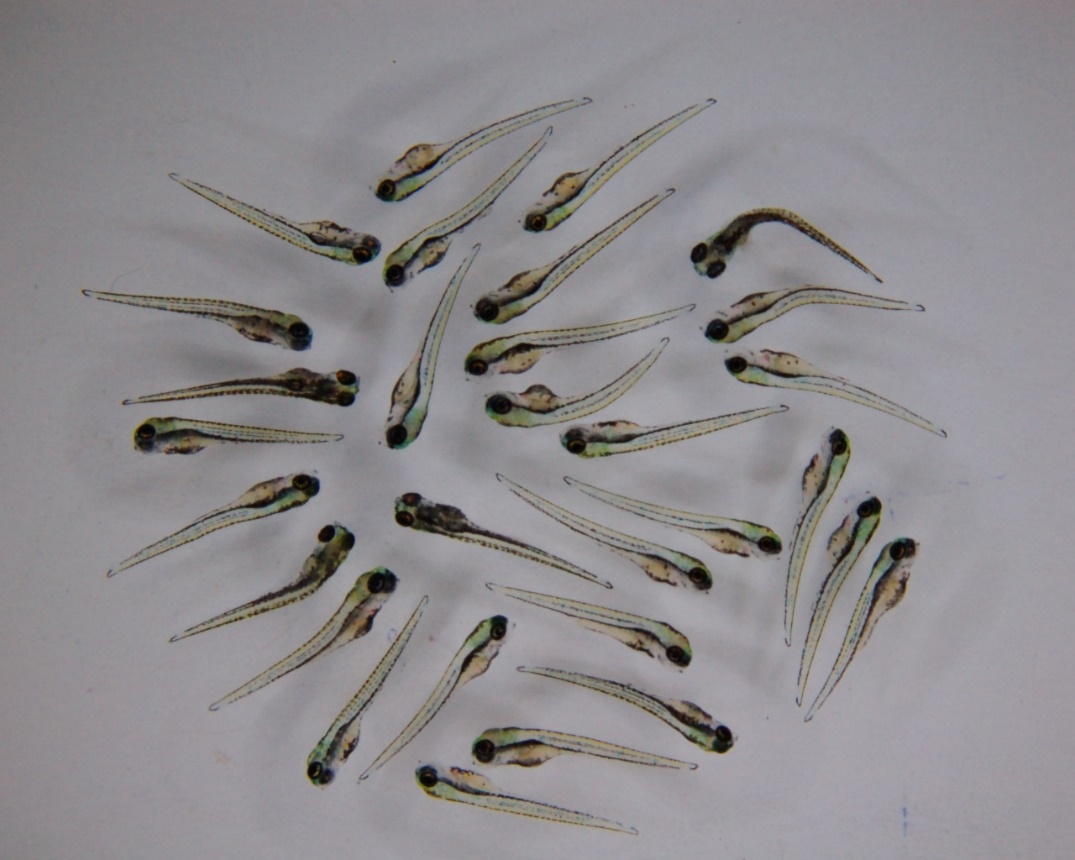


Figure 2C

*mylipb^+/+^ -*SVCV


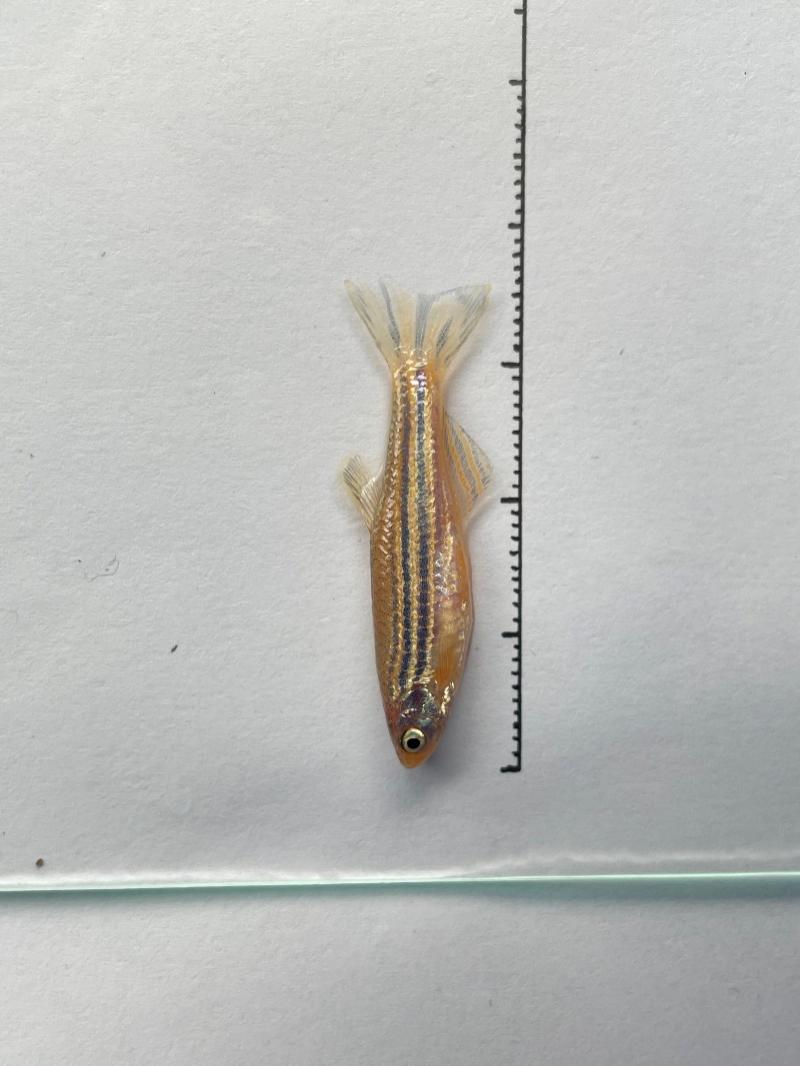


*mylipb^-/-^ -*SVCV


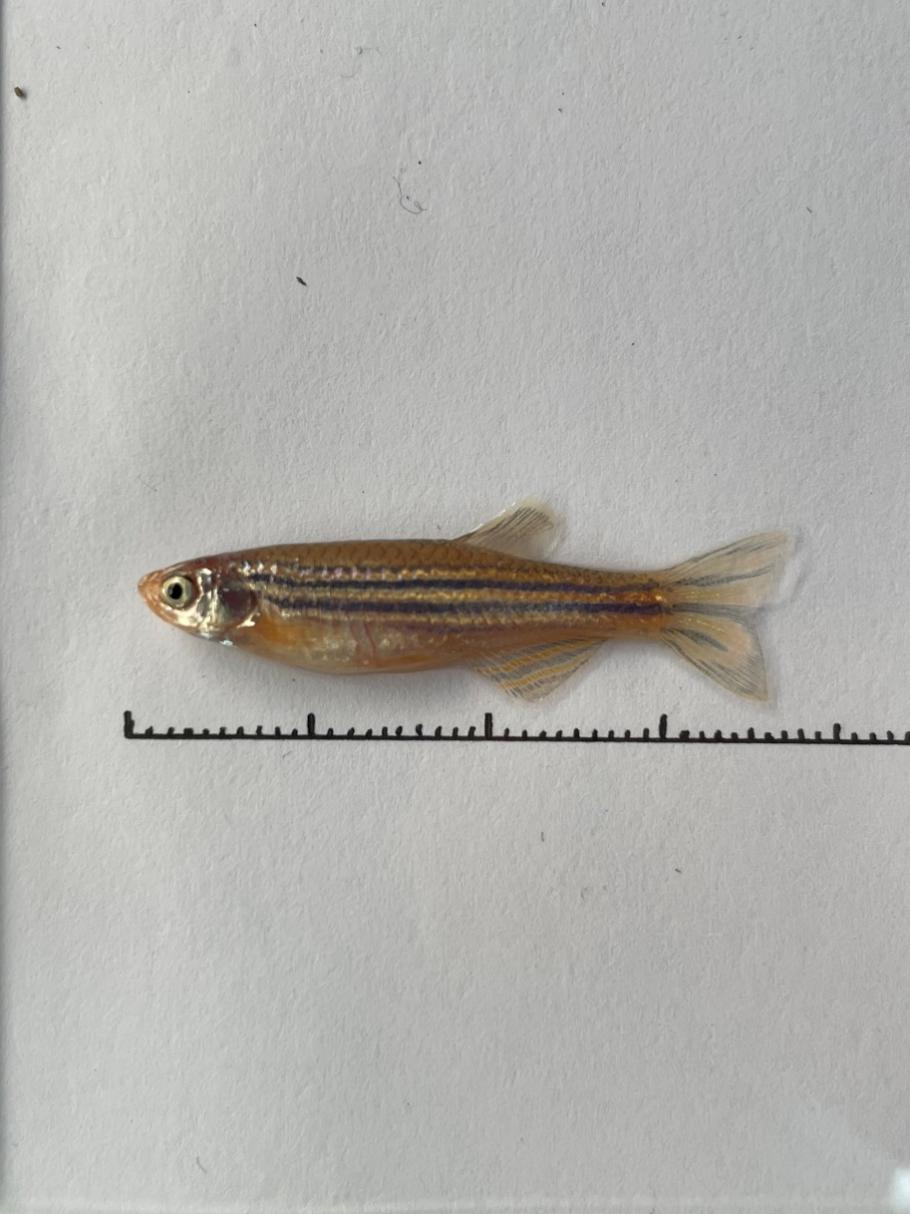


*mylipb^+/+^ +*SVCV


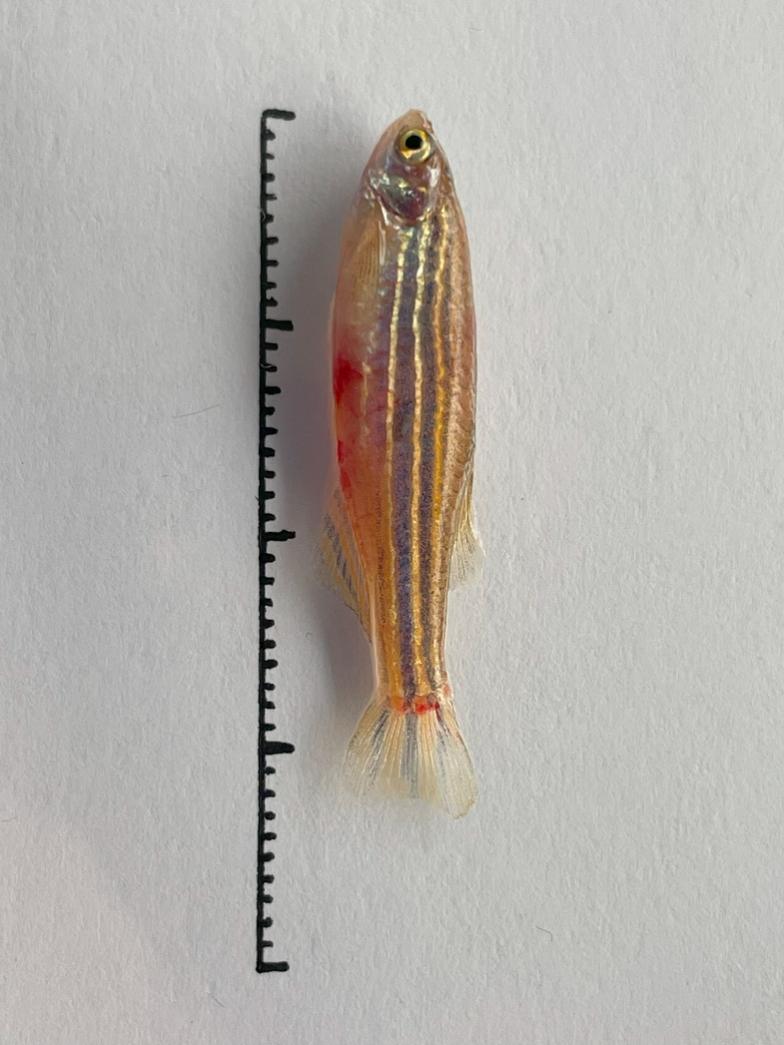


*mylipb^-/-^ +*SVCV


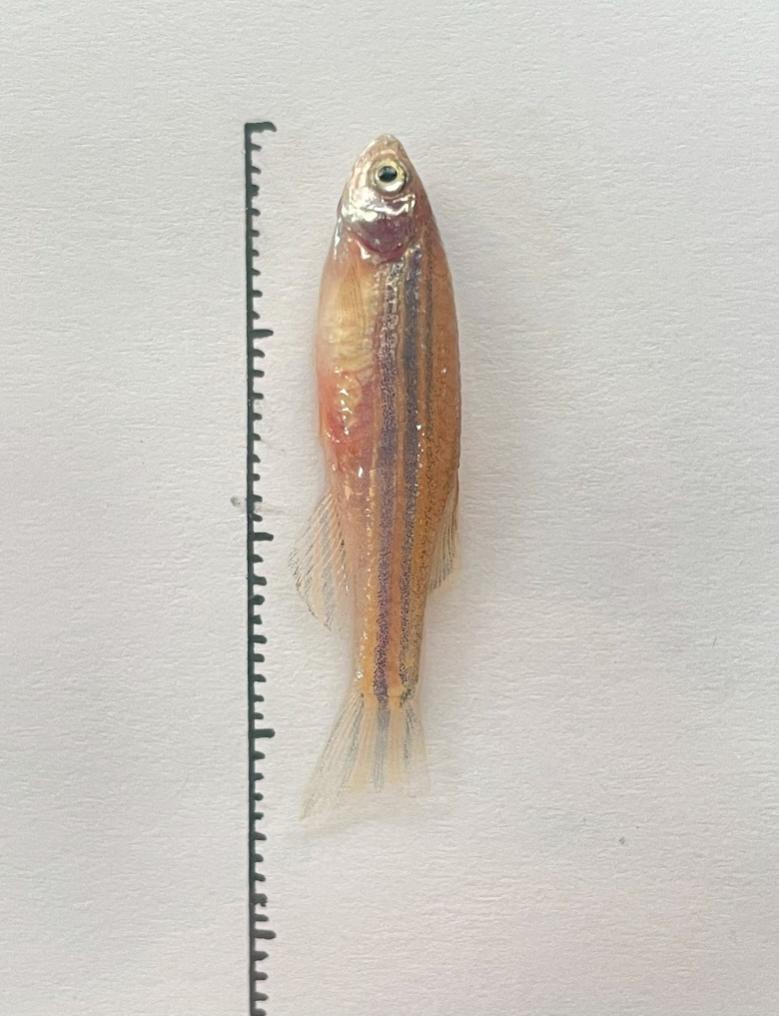

Supplement: S3 Data — (DOCX) [file ppat.1012227.s014.docx]
