# Supplementary material for: Zebrafish mylipb attenuates antiviral innate immunity through two synergistic mechanisms targeting transcription factor irf3
Source: PLoS Pathog. 2024 May 13;20(5):e1012227. doi: 10.1371/journal.ppat.1012227 (PMC11115282; doi:10.1371/journal.ppat.1012227)
Supplement: Attachment — Submitted filename: Response to Reviewers.docx [file ppat.1012227.s015.docx]

Dear reviewers：

Here are point-by-point responses to the reviewer's comments.

**Reviewer's Responses to Questions**

**Part I - Summary**

Reviewer #1: These authors report zebrafish mylipb negatively regulates IRF3-mediated antiviral innate immunity through two synergistic mechanisms. 1) Mylipb promotes K6-linked polyubiquitination and autophagic degradation of IRF3. 2) Mylipb also acts as a decoy substrate for the phosphokinase tbk1 to attenuate irf3. Although the in vivo and in vitro experiments with overexpression and KO system convincingly support the view that zebrafish mylipb negatively regulates IFN-I signaling. However, the results that refer to the proof of regulatory mechanism are superficial (Fig 4, 5, 7), and some results are inconsistent. Furthermore the western blot experiments are conducted only with overexpression system.

Reviewer #2: In this manuscript, the authors report a role of mylipb in inhibiting irf3 functions in zebrafish. Using overexpression and under-expression strategies, the results suggest that mylipb inhibits virus-induced type-I IFN production via two synergistic mechanisms - induction of autophagic degradation of irf3 and inhibition of irf3 phosphorylation. mylipb interacts with irf3 and targets Lys352 for K6-linked polyubiquitination leading to autophagy-mediated degradation. Furthermore, mylipb gets phosphorylated by tbk1 and functions as a competitive inhibitor of irf3, which also works as a substrate for tbk1. Using this mechanism mylipb acts as a decoy substrate for tbk1, thereby attenuating phosphorylation of irf3 and cellular antiviral responses. Finally, they have demonstrated the physiological role of mylipb in vivo; knockout zebrafish are more resistant to SCVC infection due to increased viral replication and antiviral gene expression levels. Overall, there are several strengths in this study, including complementary mechanistic and physiological approaches and strong biochemical analyses. However, there are some weaknesses that need to be addressed.

**Part II – Major Issues: Key Experiments Required for Acceptance**

**Reviewer #1:**

**1**. It is necessary to study the phosphorylation, stability, ubiquitination of IRF3 in mylipb-KO cells, together with the proof of the endogenous IRF3.

**Response:** Thank you for suggesting the addition of experimental data. In this revision, we have performed the experiments as suggested by the reviewer and prepared Fig 4F and Fig 5F . From Figure 4F, you can see that upon SVCV infection, irf3 protein level and phosphorylation of irf3 were increased in mylipb-null zebrafish liver (*mylipb*^-/-^) compared with WT zebrafish (*mylipb*^+/+^). From Figure 5F, ubiquitination of endogenous irf3 was decreased in *mylipb*-null zebrafish (*mylipb*^-/-^) compared with WT zebrafish (*mylipb*^+/+^). These data are in consistent with overexpression data.

**2.** From Fig 4G to 4K. The author proved that Mylipb induces autophagic degradation of IRF3 with WB and IF experiments. Several questions arise from this:

1) In fig 4K, the IF result indicated that overexpression of mylipb induce the LC3 puncta formation. LC3 puncta formation is a sign of autophagosome flux, indicating more LC3-I have been cleaved into the LC3-II. However, in fig 4J, the amount of lc3-II did not distinctly increase when overexpressing mylipb, indicating that mylipb did not induce the autophagosome flux. So the IF and IB results are inconsistent.

2) It is skeptical that the green signal of LC3 is undetected in the control group in fig 4K. In fig 4H, the bands of LC3-I and LC3-II between control group and mylipb-transfected group are without any difference. Whether the result is representative.

**Response:** We fully understand the concerns of the reviewer. We redesigned and performed the experiments for these inconsistent results. As shown in Fig 4J and 4K, overexpression of *mylipb* dose-dependently decreased the expression irf3 and increased the levels of LC3-II (Fig 4J). Confocal microscopy analysis revealed that GFP-LC3 puncta were significantly accumulated in cells overexpressing mylipb and irf3(Fig 4K). To further strengthen this conclusion, HEK293T knockout cells for the essential autophagy genes ATG5 and BECN1 were used, and we found that the irf3 degradation induced by mylipb was detectable in wild-type (WT) cells but not in either ATG5 KO or BECN1 KO cells (Fig S3C). Overexpression of *mylipb* reduced the expression irf3 and increased the levels of LC3-II in wild-type (WT) cells but not in ATG5 KO cells (Fig S3D) .

**Reviewer #2:**

1. The specificity of mylipb for irf3 is not solid. They should test if mylipb also targets irf7 and nf-kb proteins.

**Response:** Thank you for suggesting the addition of experimental data. In this revision, we have performed the experiments as suggested by the reviewer and prepared Fig S10. Co-immunoprecipitation assays demonstrated that mylipb pulled down irf7 but not NF-κB–p65 in HEK293T cells (Fig S10A-B). Overexpression of mylipb resulted in a dose-dependent reduction of overexpressed irf7 protein (Fig S10C). However, we found that the irf7 degradation induced by mylipb was detectable both in wild-type (WT) cells and ATG5 KO or BECN1 KO cells (Fig S10D). As shown in Fig S10E , mylipb induced the degradation of irf7 dependent of its ubiquitin ligase activity (Fig S10E). Using promoter assays, we found that overexpression of mylipb-WT suppressed the ISRE and IFN reporter induced by irf7(Fig S10F-G). These results suggest that mylipb also targets irf7, but is mechanistically distinct from irf3. We plan to investigate this further at a later stage.

**Fig S10**

2. Inflammatory genes also contribute to the viral pathogenesis and induction of some of these genes should be tested in the whole fish study.

**Response:** Yes, we agree with the reviewer. In this revision, we have performed the experiments as suggested by the reviewer and prepared Fig S2. We compared the mRNA expression of inflammation factor, including *il1β, il8, tnfa*, between *mylipb*^-/-^ and WT larvae following SVCV infection. qRT-PCR assay showed that the transcription of these genes induced by SVCV was significantly decreased in *mylipb*^-/-^ larvae compared to WT larvae (Fig S2A). In addition, overexpression of mylipb up-regulated the expression of inflammatory genes *il1β*, *il8*, *tnfa* in EPC cells upon SVCV stimulation(Fig S2B). Previous reports suggest that the IRF3 interacts with NF-κB–p65, thereby inhibiting virus-induced inflammatory gene induction (Sonam Popli, *PNAS*, 2022. https://doi.org/10.1073/pnas.2121385119). We speculate that the increased expression of inflammatory factors by mylipb may also be irf3 mediated.

3. The species with slower mobilities in Fig 7 for irf3 and tbk1 were considered as phosphoproteins. However, some more specific studies, e.g., either phospho-specific antibodies or phosphatase should be used to come to a reasonable conclusion.

**Response:** Yes, we agree with the reviewer. In this revision, we have performed the experiments as suggested by the reviewer and prepared Fig S7. The amount of tbk1-phosphorylated irf3(A) and mylipb(B) were reduced by CIP treatment.

4. In Fig 5E, wt mylipb did not degrade irf3 protein, as expected. There was no explanation for this and also the mutant mylipb was used in this expt and for functional studies in Fig 6. mylipb mutant should be tested in the ubiquitination studies as well.

**Response:** Thanks. In previous Fig 5E old, wt mylipb could degrade irf3 protein according to ImageJ relative quantification. However, to avoid further confusion, we performed this experiment once again and prepared Fig 5E new.

We also tested mylipb mutant in the ubiquitination studies. In the presence of mylipb-WT, irf3 ubiquitination was dramatically increased (Fig 5C). However, substitution of the cysteine at position 381 with serine (C381S) in mylipb abrogated irf3 polyubiquitination (Fig 5C)

**5**. Autophagy-mediated degradation of irf3 has only been tested using inhibitors, which have off-target effects; genetic approaches (e.g., knockdown of autophagy components) should be used for validation.

**Response:** Thanks, that is a very good suggestion. In this revision, we have performed the experiments as suggested by the reviewer and prepared Fig S3C. HEK293T knockout cells for the essential autophagy genes ATG5 and BECN1 were used, and we found that the irf3 degradation induced by mylipb was detectable in wild-type (WT) cells but not in either ATG5 KO or BECN1 KO cells (Fig S3C)

**Part III – Minor Issues: Editorial and Data Presentation Modifications**

**Reviewer #1:**

**1**. In fig 4A, the ISRE sequence is cloned from which species sample, because you have already labelled the IFN-luc with EPC or Dr. What are its sources?

**Response:** ISRE is not derived from a specific species.The full name of ISRE is IFN stimulated response element. It is conserved in mammals and vertebrates, containing consensus sequence(A/GNGAAANNGAAACT). The ISRE-luc reporter construct containing five ISRE motifs in series was purchased from Stratagene (Shun Li, *The Journal of Immunology*, 2014. https://pubmed.ncbi.nlm.nih.gov/24958903/).

EPC-IFN-luc can be used to detect changes in the IFN promoter in EPC (Epithelioma papulosum cyprini) cells. EPC-IFN-luc in the pGL3-Basic vector (Promega) were constructed as described in this article (Ste´phane Biacchesi, *Plos One*, 2012. https://pubmed.ncbi.nlm.nih.gov/23091644/).

Dr-IFN-luc can be used to detect changes of zebrafish IFN promoter. Dr-IFN-luc in the pGL3-Basic vector were constructed as described in this article (Fan Sun, *The Journal of Immunology*, 2011. https://pubmed.ncbi.nlm.nih.gov/21795596/).

2. In fig 5D, we can see that only mylipb-wt and 1-416 can induce the degradation of IRF3, while in fig 5B, the result is undetected in TCL.

**Response:** Thanks to the reviewer's careful review. In fig 5B, to avoid significant inhibition of irf3 protein levels by mylipb wild type or some mutants, we treated transfected cells with 3-MA for 8 hours before cell collection. In this revision, We have made correction.

**3**. For the ubiquitination assay result, three questions arise from this:

1) In fig 5C, IRF3 ubiquitination in △380-416 and C381S groups are still increased compared with the control group. So the result that E3 ubiquitin ligase activity was required for mylipb-mediated irf3 ubiquitination is skeptical. The ubiquitination assay of mylipb (1-280), (1-380), (1-416) should also conducted.

**Response:** Yes, we agree with the reviewer. In this revision, we first corrected the incorrect number of amino acids annotation and re-performed the experiments as suggested by the reviewer and prepared Fig S5C and Fig S5A. In the presence of mylipb-WT or mylipb-1-416aa, irf3 ubiquitination was dramatically or partly increased (Fig 5C, Fig S5A). However, substitution of the cysteine at position 381 with serine (C381S), deletion of the ZF domain (ΔZF) in mylipb or mylipb-1-279aa, mylipb-1-380aa abrogated irf3 polyubiquitination (Fig 5C, Fig S5A).

2) In fig 5F, the ubiquitination band is undetected in the K6R group, indicating that only K6-linked polyubiquitination chains can modify IRF3. However, in fig 5G, irf3 can be modified by any types of polyubiquitination chains. These results are contradictory.

**Response:** We fully understand the concerns of the reviewer. In fig 5F, the reason of ubiquitination band was undetected in the K6R group is that mylipb unable to increase K6R-linked ubiquitination of irf3, so the protein signal is masked. We performed the corresponding experiment alone, as shown in Fig S5B, K6R linked ubiquitination of irf3 can be detected but cannot be increased by mylipb.

3)It is suggested that 3-MA should appeared in ubiquitination assay results of fig 5.

**Response:** We revised accordingly.

**4.** In fig 7A, 7H, when transfected with TBK1, The HA-IRF3 has three bands, I want to know which band is unphosphorylated IRF3. If the three bands represent the whole protein level of IRF3, why both mylipb-wt and mylipb-C381S can induce the reduction of IRF3 protein level, which is contradictory with previous results.

**Response:** We fully understand the concerns of the reviewer. We consider that the shifted band must be p-irf3, and the lower band may contain the non-phosphorylated and phosphorylated irf3. For better illustration, we improved this experiment and presented as in Fig 7A. The addition of 3-MA blocked the degradation of irf3 by mylipb in the absence of tbk1, but in the presence of tbk1, with the addition of 3-MA, both mylipb wild-type and mutant reduced total irf3. We speculate that for mylpb, in addition to depending on its enzyme activity, mylipb acts as a decoy substrate for the phosphokinase tbk1 and p-mylpb may also promote p-irf3 degradation and inhibit p-irf3 levels independently of its enzyme activity. In addition, there may be other complex processes, and similar results are presented in other articles, such as Fig 9 in “A novel role of Zebrafish TMEM33 in negative regulation of interferon production by two distinct mechanisms”(Longfeng Lu, *PLOS Pathogens*, 2021. https://doi.org/10.1371/journal.ppat.1009317[)](https://doi.org/10.1371/journal.ppat.1009317)) and Fig 8G in “Zebrafish MARCH7 negatively regulates IFN antiviral response by degrading TBK1”(Shu-Ting Xiong, *International Journal of Biological Macromolecules,* 2023. https://doi.org/10.1016/j.ijbiomac.2023.124384)

5. In fig 7C, why the p-mylipb bands disappeared in TCL and IP sample.

**Response:** Thanks to the reviewer's careful review. We re-performed the immunoblot experiments，p-mylipb band appeared in TCL. Phosphorylation modification is a rapidly occurring process that cleaves the substrate once the phosphokinase has fully modified the substrate, so this may be responsible for the disappearance of the p-mylipb band in the IP sample.

6. The author declared that TBK1 phosphorylates mylipb. However, In fig 7D and E, the p-mylipb did not increase gradually with the overexpression of TBK1.

**Response:** We fully understand the concerns of the reviewer. In fig 7D and E. overexpression of tbk1 reduced the global protein level of mylipb, and we hypothesized that excessive phosphorylation would decrease mylipb protein stability.

**Reviewer #2:**

1. In Fig 1, the levels of mylipb should be provided for the overexpression and knockout studies.

**Response:** I think what you mean is In Fig 1-7, the levels of mylipb should be provided for the overexpression and knockout studies. This is a very good suggestion. Because the commercial hu-MYLIP and prepared zf-mylipb antibodies did not work well (Fig Antibody verification), we could not test the mylipb protein in the knockout studies. We have performed the experiments to test overexpressed mylipb and prepared Fig 2K, Fig 3, Fig 6, Fig 7K, Fig 7L.

**2.** In Fig 2A, dose is not mentioned; also, the rationale for choosing 18h is not mentioned.

**Response:** At 18 h after SVCV treatment, WT siblings (*mylip*b^+/+^) survival was below 50%, at which point few mylipb-null zebrafish larvae (*mylipb*^-/-^) died, so 18h was chosen for representative images. Dose of SVCV was added in Fig legend.

**3.** In Fig 3H-J the MOI of SCVC in EPC cell is missing.

**Response:** We revised accordingly.

4. In Figure 1B the titer of SCVC is mentioned but the MOI is missing.

**Response:** We revised accordingly.

5. In Figure 3D, line no 852, “After 24h, we infected (instead of transfected) the cells with SVCV”. The MOI is also not mentioned.

**Response:** We revised accordingly.

**6.** In Figure 6B and Figure 6D, MOI is missing.

**Response:** We revised accordingly.

Please do not hesitate to contact us directly if any further information is required.

Yours sincerely

Wuhan Xiao & Jing Wang
